# Convergent transcriptomic and connectomic controllers of information integration and its anaesthetic breakdown across mammalian brains

**DOI:** 10.1038/s41562-025-02381-5

**Published:** 2026-01-28

**Authors:** Andrea I. Luppi, Lynn Uhrig, Jordy Tasserie, Pedro A. M. Mediano, Fernando E. Rosas, S. Parker Singleton, Daniel Gutierrez-Barragan, Silvia Gini, Pablo Castro, Camilo M. Signorelli, Daniel Golkowski, Andreas Ranft, Rüdiger Ilg, Denis Jordan, Kanako Muta, Junichi Hata, Hideyuki Okano, Zhen-Qi Liu, Yohan Yee, Alain Destexhe, Rodrigo Cofre, David K. Menon, Alessandro Gozzi, Bechir Jarraya, Emmanuel A. Stamatakis

**Affiliations:** 1 Division of Anaesthesia and Department of Clinical Neurosciences, University of Cambridge, Cambridge, UK; 2 St John’s College, Cambridge, UK; 3 Centre for Eudaimonia and Human Flourishing, Department of Psychiatry, University of Oxford, Oxford, UK; 4 McGill University, Montreal, Quebec Canada; 5 Cognitive Neuroimaging Unit, CEA, INSERM, Université Paris-Saclay, NeuroSpin Center, Gif-sur-Yvette, France; 6 Department of Anesthesiology and Critical Care, Necker Hospital, AP-HP, Université de Paris Cité, Paris, France; 7 Center for Brain Circuit Therapeutics, Department of Neurology, Brigham and Women’s Hospital, Harvard Medical School, Boston, MA USA; 8 Department of Computing, Imperial College London, London, UK; 9 Informatics Department, University of Sussex, Brighton, UK; 10 Centre for Complexity Science, Imperial College London, London, UK; 11 Department of Radiology, Weill Cornell Medicine, New York, NY USA; 12 Centre for Neuroscience and Cognitive Systems, Italian Institute of Technology, Rovereto, Italy; 13 Center for Mind and Brain Sciences, University of Trento, Rovereto, Italy; 14 Department of Computer Science, University of Oxford, Oxford, UK; 15 Center for Philosophy of Artificial Intelligence, University of Copenhagen, Copenhagen, Denmark; 16 Laboratory of Neurophysiology and Movement Biomechanics, Université Libre de Bruxelles, Brussels, Belgium; 17 Department of Neurology, Klinikum rechts der Isar, Technical University Munich, Munich, Germany; 18 Department of Anesthesiology and Intensive Care, School of Medicine and Health, Klinikum rechts der Isar, Technical University of Munich, Munich, Germany; 19 Department of Neurology, Asklepios Clinic, Bad Tolz, Germany; 20 Graduate School of Human Health Sciences, Tokyo Metropolitan University, Arakawa, Tokyo, Japan; 21 Laboratory for Marmoset Models of Brain Diseases, RIKEN Center for Brain Science, Wako, Saitama Japan; 22 Regenerative Medicine Research Center, Keio University, Kanagawa, Japan; 23 Department of Radiology, University of Calgary, Calgary, Canada; 24 Institute of Neuroscience (NeuroPSI), Paris-Saclay University, Centre National de la Recherche Scientifique (CNRS), Gif-sur-Yvette, France; 25 INRIA Centre, Universite Cote d’Azur, Nice, France; 26 Department of Neurology, Hopital Foch, Suresnes, France

**Keywords:** Network models, Dynamical systems, Consciousness

## Abstract

The mammalian brain orchestrates the processing and integration of information to guide behaviour. Here, to characterize mammalian information-processing architecture, we combine functional neuroimaging and anaesthesia in humans, macaques, marmosets and mice. We show that breakdown of information integration is a convergent effect of diverse anaesthetics across mammalian species. As the system disintegrates, brain dynamics become more difficult to control. Both effects are reversed upon re-awakening induced by thalamic deep-brain stimulation in the macaque. Regional breakdown of integrated information coincides with the species-specific spatial topography of *PVALB/Pvalb* gene expression. To provide mechanistic insight beyond correlation, we develop computational models for humans, macaques and mice that integrate species-specific connectivity and transcriptomic gradients, demonstrating their respective roles for controlling brain dynamics and information integration. We reveal evolutionarily conserved controllers of information integration in the mammalian brain.

## Abstract (web summary)

Luppi et al. identify transcriptomic and connectomic controllers of information integration and its breakdown induced by anaesthesia in humans, macaques, marmosets and mice.

## Main

To coordinate behaviour in response to a complex environment, the central nervous system of humans and other animals must combine the information provided by diverse sensory signals^1,2^. Prominent theoretical accounts of neural computation, cognition and even consciousness converge in attributing a fundamental role to integrative processes in the brain^3–8^. However, understanding how the brain orchestrates the integration of information remains a formidable open challenge and a focus of intense investigation in neuroscience^2,9^. Addressing this challenge requires a combination of theoretical advances and suitable experimental approaches.

On the theoretical front, there is growing recognition that a full understanding of neural information processing requires disentangling qualitatively different kinds of information that can co-exist in any distributed system, including the brain^10–16^. In particular, the framework of information decomposition has revealed the existence of synergy: information that is present in the system as a whole but not in any of its individual components^2,14–18^. Here we capitalize on these recent theoretical developments to obtain a rigorous quantification of ‘integrated information’ as the information that is present in the whole system, over and above the sum of the parts.

On the experimental front, the combination of neuroimaging and general anaesthesia provides a powerful avenue to identify neurobiological controllers of the brain’s informational architecture^19–22^. Without altering brain anatomy, anaesthesia induces a drastic breakdown of information processing in the brain, as indicated by the suppression of both sensory and motor interactions between organism and environment. Unlike lesions, anaesthesia is fully reversible, making it suitable for investigating brain function in healthy humans rather than being restricted to patients. Although anaesthetic drugs vary in terms of microscale molecular targets, the macroscale effects of anaesthesia are highly conserved across evolution, reliably suppressing behavioural responsiveness across a wide variety of species including humans, non-human primates and rodents^19,21,23^. Indeed, the effects of anaesthesia on brain dynamics are similar both across species, and between anaesthesia and patients with disorders of consciousness^21,23–32^. Thus, studying brain activity under anaesthesia provides a powerful opportunity for translational discovery, by comparing how the same phenomenon manifests in the brains of humans and in other species that are more experimentally accessible^19,21,23^. Here we capitalize on each of these advantages.

**Fig. 1:**
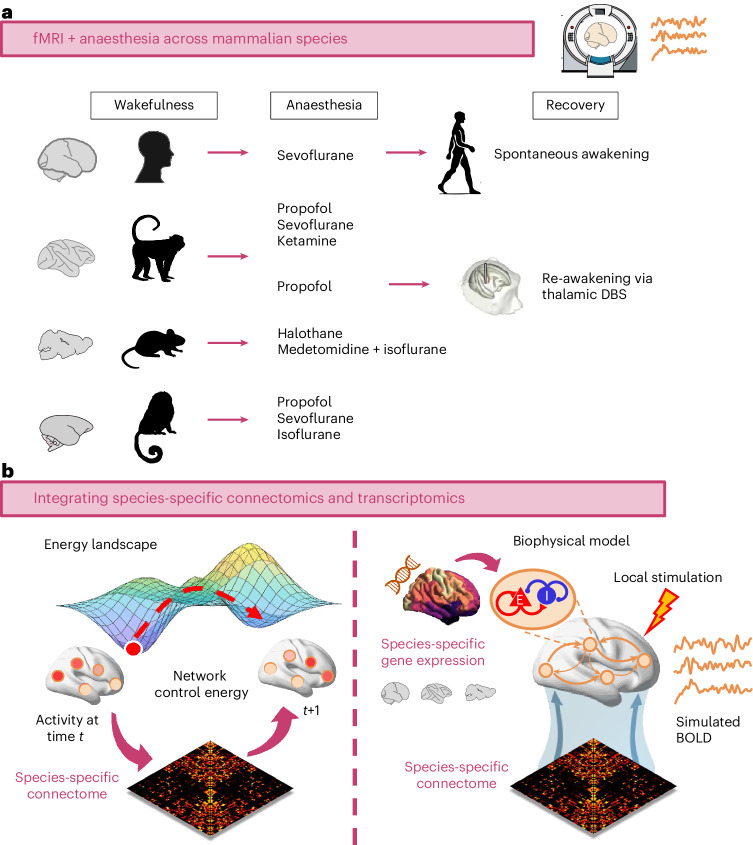
Integrating neuroimaging and pharmacology with computational modelling across species. **a**, Across four mammalian species (human, macaque, mouse and marmoset), we consider fMRI data acquired during wakefulness and under a variety of anaesthetic regimes (sevoflurane, propofol, ketamine, isoflurane, halothane and isoflurane–medetomidine). We also investigate spontaneous recovery of consciousness (in humans) and re-awakening induced by thalamic deep-brain stimulation (DBS) during continuous anaesthetic infusion in the macaque. **b**, We then use network control theory and biophysical computational modelling to provide mechanistic insights by integrating species-specific structural connectivity and species-specific gene expression in human, macaque and mouse. Credits for **a**: human head icon, walking human silhouette and marmoset icon from pixabay.com; macaque and mouse icons adapted from ref. ^133^, published under a CC-BY licence; macaque icon originally designed by Freepik.com; mouse icon originally designed by CraftStarters.com; brain icons adapted from ref. ^38^, published under a CC-BY license, and originally from SciDraw.io; MRI icon adapted from ref. ^134^, published under a CC-BY license; macaque DBS illustration adapted from ref. ^30^, published under a CC-BY licence.

The consistency of anaesthesia’s behavioural effects across species suggests the presence of an evolutionarily conserved architecture for the integration of information in the mammalian brain, representing a common target of diverse anaesthetics. Here we seek to uncover this shared architecture, by identifying changes in neural activity that are consistently induced by different anaesthetics and in different species, upon anaesthetic-induced breakdown of interaction with the environment. To this end, we systematically compare functional MRI (fMRI) activity during wakefulness and anaesthesia across four mammalian species: human (*Homo sapiens*); rhesus macaque (*Macaca mulatta*), a gyrencephalic primate; common marmoset (*Callithrix jacchus*), a lissencephalic primate; and mouse (*Mus musculus*)^29,31,33,34^ (Fig. 1a). Our strategy is threefold. First, we ask whether anaesthetic-induced breakdown of the capacity to interact with the environment corresponds to a breakdown of information integration in the brain, and whether this effect is shared across species, similar to the behavioural manifestation of anaesthesia. Second, to establish a bidirectional causal link, we combine the specificity of deep-brain stimulation with fMRI’s coverage of the entire cortex in the macaque^30^. Leveraging the experimental accessibility of non-human animals, we show that neural integration of information is restored upon re-awakening from anaesthesia induced by electrical stimulation of the macaque central thalamus, thereby demonstrating local control over global information processing across brain and behaviour.

Third, we seek to identify shared neurobiological underpinnings that control neural integration of information across species. Specifically, we focus on (1) the anatomical connectivity between brain regions, which shapes their ability to interact and exchange information^35–37^; and (2) the genetic make-up of each region’s cyto- and chemo-architecture, on which anaesthetics act at the microscale to influence local circuit dynamics. To this end, we capitalize on the recent availability of species-specific connectomics^38^ and transcriptomic databases for human^39^, macaque^40^ and mouse^41^. To provide insight beyond correlation, we develop in silico computational models that integrate species-specific brain activity, structural connectivity and gene expression^42,43^. We show that across species, the regional expression of the *PVALB*/*Pvalb* gene (a cell-type marker for inhibitory interneurons) is especially suitable for controlling brain dynamics and modulating the integration of information via regionally heterogeneous inhibition. In contrast, our model indicates that the structural connectivity of the macaque central thalamus makes it especially suitable as a focal stimulation target for restoring integration of information, replicating our in vivo deep-brain stimulation (DBS) results and providing mechanistic insight about their connectomic origin. Overall, our multimodal, multispecies approach reveals evolutionarily conserved local and global controllers of information integration in the mammalian brain.

## Results

### Integrated information from information dynamics

In a system such as the brain, the spontaneous evolution of regional dynamics is not random but is rather partly dependent on its previous state. This means that past states hold information about future states^2^. This ‘intrinsic’ information (We also note that Shannon’s well-known definition is not the only way to operationalize information; in particular, a stronger notion of what it means for information to be ‘intrinsic’ has been recently formalized, leading to alternative interpretations of integrated information that are beyond the scope of classic information theory. See Supplementary Discussion for alternative development of integrated information beyond IIT 2.0.) contained in the system’s spontaneous dynamics can be characterized using information theory in the absence of any explicit tasks, by means of the time-delayed mutual information (TDMI): mutual information between the past and future timeseries of regions *X* and *Y*, reflecting the amount of information flowing from the system’s past to its future. This approach is fully analogous to how the commonly used functional connectivity (statistical correlation between the timeseries of regions *X* and *Y*) is used to study the brain’s ‘intrinsic connectivity networks’.

**Fig. 2:**
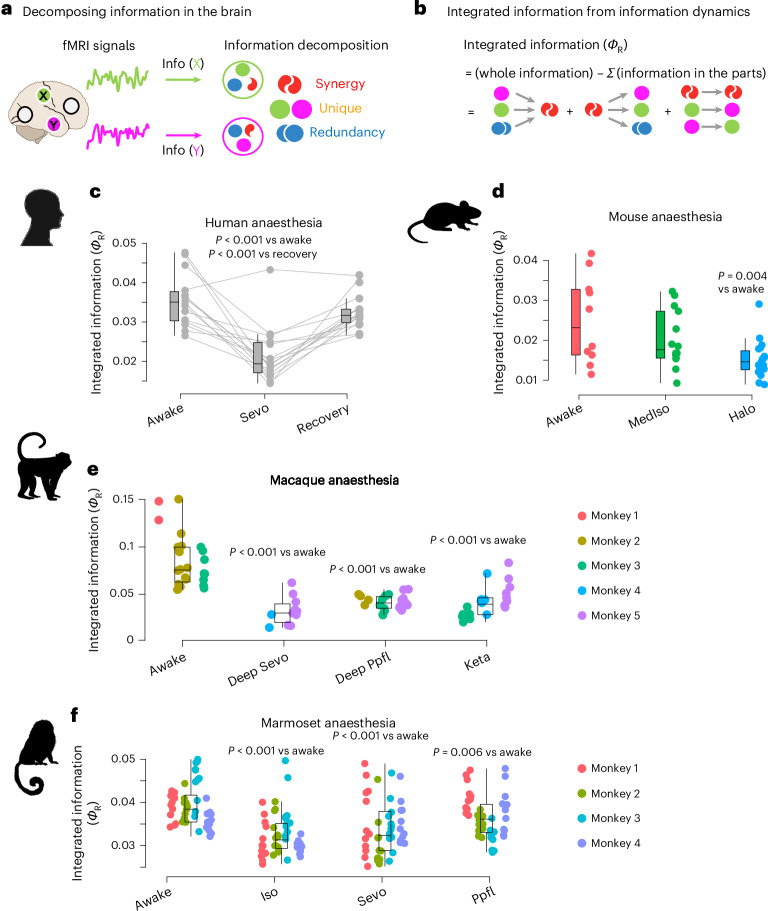
Anaesthesia disintegrates the mammalian brain. **a**, The total information jointly carried by two variables *X* and *Y* (for example, two brain regions) can be exhaustively decomposed into information that is redundantly carried by both variables (blue); or uniquely by each (green and orange); or synergistically by considering the two variables together (red). Various information dynamics phenomena can then take place as the different types of information evolve over time from past to future. **b**, We can obtain ‘integrated information’ as the sum of all information-dynamic phenomena that reflect interactions between the system’s components^16^. Equivalently, we can obtain integrated information as the difference between information in the whole system and information in the sum of the system’s parts considered in isolation (that is, any information that remains in the same variable over time). This measure of integrated information, termed *Φ*_R_, overcomes the limitations of an influential early proposal (*Φ*_2008_) which was shown to double count the redundant information contained in the parts, leading to negative values and other paradoxical results^16^ (see Methods and Extended Data Fig. 1 for an explanation of this double counting and how this issue is resolved by the use of information decomposition). Intuitively, integrated information will be low if there is little differentiation among elements of the system, such that they are just redundant copies and considering them together provides no additional advantage; or if there is low integration, such that the parts behave independently without influencing each other. Supplementary Figs. 1–3 provide examples of different systems and their integrated information. For each pair of brain regions, we quantify their integrated information by applying information decomposition to their fMRI timeseries and summing the values of the corresponding information dynamics. A whole-brain value is then obtained by averaging across all pairs of brain regions. **c**, Human sevoflurane anaesthesia (Sevo) versus wakefulness (two-sided paired-samples *t*-test, FDR-corrected for multiple comparisons) and versus post-anaesthetic recovery (two-sided paired-samples *t*-test, FDR-corrected for multiple comparisons); *n* = 15. **d**, Mouse wakefulness (*n* = 10) versus medetomidine–isoflurane (MedIso; *n* = 14) and halothane (Halo; *n* = 19) anaesthesia data. *P* values are from two-sided independent-samples *t*-test, FDR-corrected for multiple comparisons against awake condition. **e**, Macaque wakefulness versus anaesthesia with Ppfl, Sevo and Keta. *N* = 24 runs from 3 animals for awake; 11 runs from 2 animals for sevoflurane; 23 runs from 3 animals for propofol; and 22 runs from 3 animals for ketamine anaesthesia. *P* values are from linear mixed-effects models (two-sided), FDR-corrected for multiple comparisons against the awake condition (see Methods). Datapoints with the same colour indicate the same animal. **f**, Marmoset wakefulness versus anaesthesia with isoflurane (Iso), Sevo and Ppfl. *N* = 48 runs from 4 animals for each condition. *P* values are from linear mixed-effects models (two-sided), FDR-corrected for multiple comparisons against the awake condition (see Methods). In all boxplots: central line, median; box limits, upper and lower quartiles; whiskers, 1.5× interquartile range. Datapoints with the same colour indicate the same animal. See Source data for full statistical reporting. Credits: brain icon in **a** from SciDraw.io. Human head icon in **c** from pixabay.com. Mouse icon in **d** designed by CraftStarters.com. Macaque icon in **e** designed by Freepik.com. Marmoset icon in **f** from pixabay.com. Source data

**Extended Data Fig. 1:**
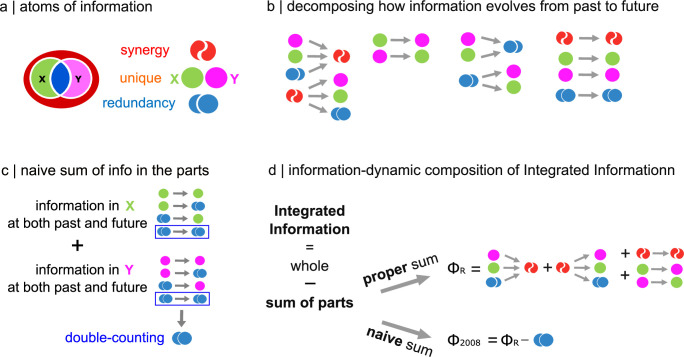
Integrated information from information decomposition. (**a**) Partial information decomposition distinguishes information that is uniquely provided by variable *X* or by variable *Y*; redundant information that is equally available from either variable; and synergistic information, which is only available when considering both variables jointly, but not either of them in isolation. (**b**) Information decomposition enables tracking how information from the system’s past to its future is carried by its constituent elements, corresponding to each of the 4×4 = 16 combinations of redundancy, *X*-unique information, *Y*-unique information, and synergy. (**c**) Naïve sum of the parts. Some information can be obtained by considering element *X* alone, without reference to any other parts of the system. This information corresponds to the 4 possible combinations of redundant information and *X*-unique information across past and future. Likewise, some information can be obtained by considering element *Y* alone, given by the combinations of redundancy and *Y*-unique information. However, if we simply sum the information that can be found in *X* without reference to *Y*, and the information that can be found in *Y* without reference to *X*, then this ‘naïve sum of the parts’ will double-count the information that is redundantly present in both *X* and *Y* across past and future (persistent redundancy). (**d**) Integrated information is the information that is present in the system as a whole, over and above the sum of the information provided by each of the parts. However, attempting to quantify this ‘whole minus sum’ by subtracting the naïve sum of the parts from the total information flowing between past and future of the system, yields the original measure of integrated information from Balduzzi and Tononi (*Φ*_2008_), which has well-known conceptual difficulties including negative values for redundancy-dominated systems (Mediano et al., 2025). If instead the proper sum of the parts is used (that is, without double-counting the persistent redundancy), we obtain the revised measure of integrated information from (Mediano et al., 2025), *Φ*_R_. In turn, this means that we can re-express *Φ*_*2008*_ as the balance (difference) between integrated information (*Φ*_*R*_) and the persistent redundancy.

Crucially, recent advances in the theory of information decomposition have demonstrated that brain dynamics carry multiple qualitatively different kinds of information, going beyond traditional measures of functional connectivity^2,17,18,44–46^. In particular, two variables, such as two brain regions, can carry information about a target in three fundamentally distinct ways (Fig. 2a): (1) if each variable provides the same information as the other, this is redundant information; (2) unique information refers to information that only one of the variables carries and the other does not; and (3), information can be carried synergistically, if it is available only when considering both variables together but not when considering either of them in isolation^15,16^ (Fig. 2a). For example, much of human depth perception is conveyed synergistically by the eyes, since closing either eye will greatly impair depth perception^2^. When viewed as the information that the past of the system provides about its future, the time-delayed mutual information can be exhaustively decomposed into a formal taxonomy of ‘information dynamics’: distinct combinations of synergistic, unique and redundant information between *X* and *Y* (Extended Data Fig. 1)^14,16^. For example, if there is unique information in region *X* in the past about unique information in region *Y* at a later point in time, this can be described as reflecting ‘information transfer’ from *X* to *Y*.

This taxonomy of information dynamics enables a formal characterization of ‘integrated information’ in distributed systems. The key insight is that if the elements of a system form a coherent whole, then considering the system as a whole should provide additional information about its future behaviour, beyond the information that is already provided by the individual parts about their own future behaviour. This whole-minus-sum information is the ‘difference that makes a difference’: information that arises from how the parts of the system interact with each other^47^. However, an initial attempt to quantify this whole-minus-sum information (here termed *Φ*_2008_) was later found to involve subtracting the information that is persistently redundant between *X* and *Y* (ref. ^16^). Thus, the lens of information dynamics explained why this apparently intuitive measure produced such counter-intuitive results, including negative values (which we now know would occur whenever the system is redundancy dominated)^48^. An effective solution was therefore proposed in the form of the revised measure of integrated information, *Φ*_R_, by simply adding back the redundancy term^16^.

Here we demonstrate that *Φ*_R_ is not just a pragmatic solution to the shortcomings of *Φ*_2008_: in fact, *Φ*_R_ represents a conceptually more appropriate measure of whole-minus-sum integrated information. This is because the subtraction of persistent redundancy involved in *Φ*_2008_ arises from a naïve way of quantifying the sum of the parts. Specifically, redundant information (which by definition is present in both *X* and *Y*) is included both when computing the information in *X* and when computing the information in *Y*, and therefore it ends up being double counted in the subtraction from the total information when computing *Φ*_2008_. In contrast, *Φ*_R_ corresponds to the total information minus the ‘proper’ sum of the parts (that is, without mistakenly double counting the persistent redundancy as being independently contributed by both *X* and *Y*; see Methods for the mathematical formalism and Extended Data Fig. 1 for a visual illustration). Thus, the framework of information dynamics reveals *Φ*_R_ as a principled quantification of integrated information (Fig. 2b).

### Breakdown of integrated information is a convergent effect of diverse anaesthetics across mammalian species

Equipped with *Φ*_R_ as a rigorous quantification of integrated information, we systematically investigate integrated information between each pair of brain regions in humans, macaques, marmosets and mice, on the basis of their haemodynamic fMRI timeseries. This approach is fully analogous to pairwise correlation between regional timeseries that is commonly used to quantify ‘functional connectivity’ but reflecting a more nuanced set of dynamical phenomena in the system^14,16–18^. Specifically, our datasets comprise *N* = 15 human volunteers undergoing fMRI scanning while awake and during deep anaesthesia with sevoflurane, as well as spontaneous recovery of responsiveness^34^; *N* = 5 macaques undergoing repeated scanning while awake and during anaesthesia with sevoflurane, propofol or ketamine^29,49^; *N* = 4 marmoset monkeys each scanned 12 times during wakefulness or anaesthesia with sevoflurane, propofol or isoflurane^33^; and *N* = 43 mice scanned either during wakefulness or during anaesthesia with halothane or combined medetomidine–isoflurane^31^.

We find that anaesthetic-induced disconnection from the environment significantly reduces the brain’s mean value of integrated information across all pairs of regions. This result is consistently observed across species and anaesthetics (except for medetomidine–isoflurane in the mouse) (Fig. 2c–f). Thus, anaesthesia consistently reduces the mammalian brain’s capacity to integrate information at the macroscale. However, is integrated information also restored upon awakening from anaesthesia? Indeed, we find that this is the case: integrated information in the human brain increases significantly upon spontaneous recovery of responsiveness following discontinuation of the anaesthetic (Fig. 2c).

### Integration of information is restored upon re-awakening induced by central thalamic DBS

In addition to suppressing responsiveness and presumably consciousness, each anaesthetic is also likely to induce drug-specific side effects on physiology, such as changes in breathing, heart rate and vascular tone. By identifying changes in neural activity that are consistently induced by diverse drugs in different species, we can narrow down how anaesthetics act on the brain to induce the same behavioural outcome across species (breakdown of interaction with the environment) and exclude side effects that are drug or species specific. The combination of fMRI and dual causal manipulation with anaesthesia and deep-brain stimulation provides a rare opportunity for additional nuance. We can single out aspects of brain function that specifically co-vary with the anaesthetic’s consciousness-suppressing effects, being not only perturbed by anaesthesia but also restored when DBS induces recovery of behavioural responsiveness.

**Fig. 3:**
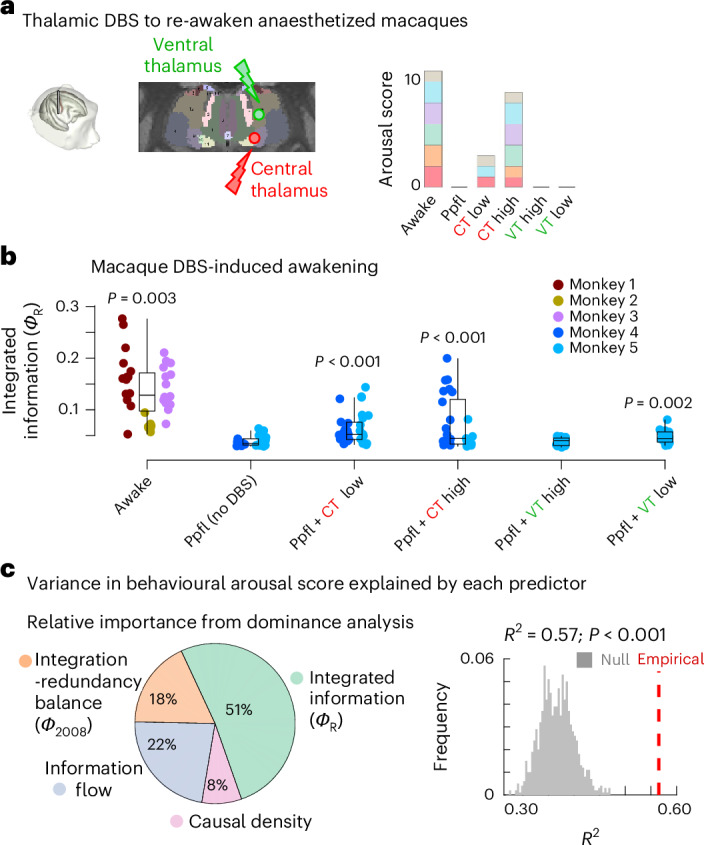
Integrated information is restored upon DBS-induced recovery of consciousness and tracks behavioural arousal better than alternative information-dynamic measures. **a**, Tasserie and colleagues^30^ delivered deep-brain stimulation to the centro-median thalamus (CT) or ventrolateral thalamus (VT) during fMRI scanning in *N* = 2 anaesthetized macaques at either high intensity (5 V) or low intensity (3 V). CT stimulation consistently restored behavioural arousal. Panel adapted from ref. ^30^, published under a CC-BY licence. **b**, Integrated information for macaque across wakefulness and propofol anaesthesia with and without deep-brain stimulation of different thalamic sites and intensities. *P* values are from linear mixed-effects models (two-sided), FDR-corrected for multiple comparisons against propofol anaesthesia without DBS (‘No-DBS’ condition; see Methods). *N* = 36 runs from 3 animals for awake; 28 runs from 2 animals for anaesthesia (DBS-off); 31 runs from 2 animals for low-amplitude centro-median thalamic DBS; 25 runs from 2 animals for high-amplitude centro-median thalamic DBS; 18 runs from 1 animal for low-amplitude ventrolateral thalamic DBS; 18 runs from 1 animal for high-amplitude ventrolateral thalamic DBS. Datapoints with the same colour indicate the same animal. Boxplots: central line, median; box limits, upper and lower quartiles; whiskers, 1.5× interquartile range. See Source data for full statistical reporting. **c**, Dominance analysis determines the relative contribution of each independent variable to the overall fit (adjusted *R*^2^) of a multiple linear regression model^54^, partitioning the total variance explained in the target (here, behavioural arousal score) accounted for by each predictor. Here, our predictors are the integrated information (*Φ*_R_) from ref. ^16^; the measure of integration-redundancy balance (*Φ*_2008_) from ref. ^47^; causal density^50,131^; and net information flow (see Methods for details of each). Regression target is the behavioural arousal score from each animal in the DBS macaque dataset^29,30^. Percentage of relative importance is represented as a pie chart, revealing that integrated information is the predictor with highest relative importance, accounting for 51% of the total variance explained. We establish the statistical significance of the multiple linear regression model accounting for arousal score as a function of our fMRI information-dynamic measures model using a non-parametric permutation test (one-sided), by comparing the empirical variance explained against a null distribution of *R*^2^ obtained from repeating the multiple regression with randomly reassigned arousal scores. The empirical variance explained is significantly greater than chance (*R*^2^ = 0.57; *P* < 0.001). Source data

We take this approach by considering an independent dataset of macaque fMRI acquired during propofol anaesthesia and during restoration of behavioural responsiveness induced by deep-brain stimulation of the central thalamus (Fig. 3a)^30^. We first use this independent dataset to replicate our observation that propofol anaesthesia reduces integrated information in the macaque (Fig. 3b). Crucially, we next show that integrated information in the macaque brain is significantly increased compared with propofol anaesthesia, upon in-scanner electrical deep-brain stimulation of the central thalamus (CT) at high (5 V) and even at low (3 V) intensity (Fig. 3b). These results go beyond the mere injection of current: the same stimulation protocols were also applied to a control site in the ventrolateral thalamus (VT), eliciting no behavioural effect^30^. Likewise, there is no statistical evidence of integrated information being affected by high-intensity stimulation of the ventrolateral thalamus, although a smaller but significant increase occurs for low-intensity stimulation (Fig. 3b). Notably, the greatest restoration of integrated information is observed upon CT stimulation at high intensity, which also has the greatest effect on behavioural arousal. Overall, breakdown of integrated information only occurs when the anaesthetic induces breakdown of interaction with the environment, but not when this effect is countered by central thalamic stimulation. In other words, integrated information co-varies with the consciousness-suppressing effect of anaesthetics, not with their mere presence in the system.

To further interrogate the link between integrated information and the behavioural effect of anaesthesia, we consider the ability of integrated information to track behavioural arousal and its restoration by thalamic DBS across all animals and conditions together. Specifically, we use dominance analysis, a multivariate technique that distributes the fit of a statistical model across predictors, such that the contribution of each predictor can be assessed and compared to that of other predictors, reflecting the proportion of the explained variance that can be attributed to each predictor. Here, our model’s target is the behavioural score on the preclinical arousal scale (see Methods and Supplementary Table 1), which ranges from 0 (complete unresponsiveness and lack of arousal) to 11 (fully awake and alert). In addition to integrated information, we also consider prominent information-dynamic quantities that can be obtained from information decomposition. Specifically, we consider (1) the original (that is, uncorrected) proposed measure of integrated information, *Φ*_2008_, which was put forward as a measure of conscious level in the brain^47,48,50^. As shown in Extended Data Fig. 1 (see also Methods), *Φ*_2008_ can be more properly understood as quantifying the balance between integrated information (*Φ*_R_) and the persistent redundancy in the system. We also consider (2) causal density, another measure that seeks to quantify the ‘relevant complexity’ in a system in terms of overall (statistical) causal interactivity between its elements, and was likewise proposed as a candidate measure of conscious level in the brain^50^. Finally, we consider the overall net flow of information, which quantifies the prevalence of imbalanced information transfer in the system. Measures of information transfer such as transfer entropy can exhibit changes across anaesthesia and wakefulness, including due to thalamic stimulation^51^. In addition, presence of a net flow of information indicates an asymmetry in the system, and therefore information flow as defined here is mathematically related to the notion of ‘temporal irreversibility’, another measure of brain organization that is often reduced in the unresponsive (and presumably unconscious) brain^52,53^.

Using dominance analysis^54^ to disentangle the relative importance of each predictor, we show that integrated information (*Φ*_R_) emerges as the predictor with highest relative importance for tracking behavioural arousal in the macaque DBS dataset, alone accounting for >50% of the total variance explained (Fig. 3c). This result is also replicated when considering both macaque datasets together (DBS and multi-anaesthesia), which used the same scale for measuring behavioural arousal and are therefore comparable (Supplementary Fig. 4). Altogether, even though each of the other three candidates has been associated with consciousness on either theoretical or empirical grounds, we find that integrated information is the measure of neural information dynamics that most closely tracks the behavioural effects of anaesthesia.

### Compromised controllability of brain dynamics in the disintegrated mammalian brain

**Fig. 4:**
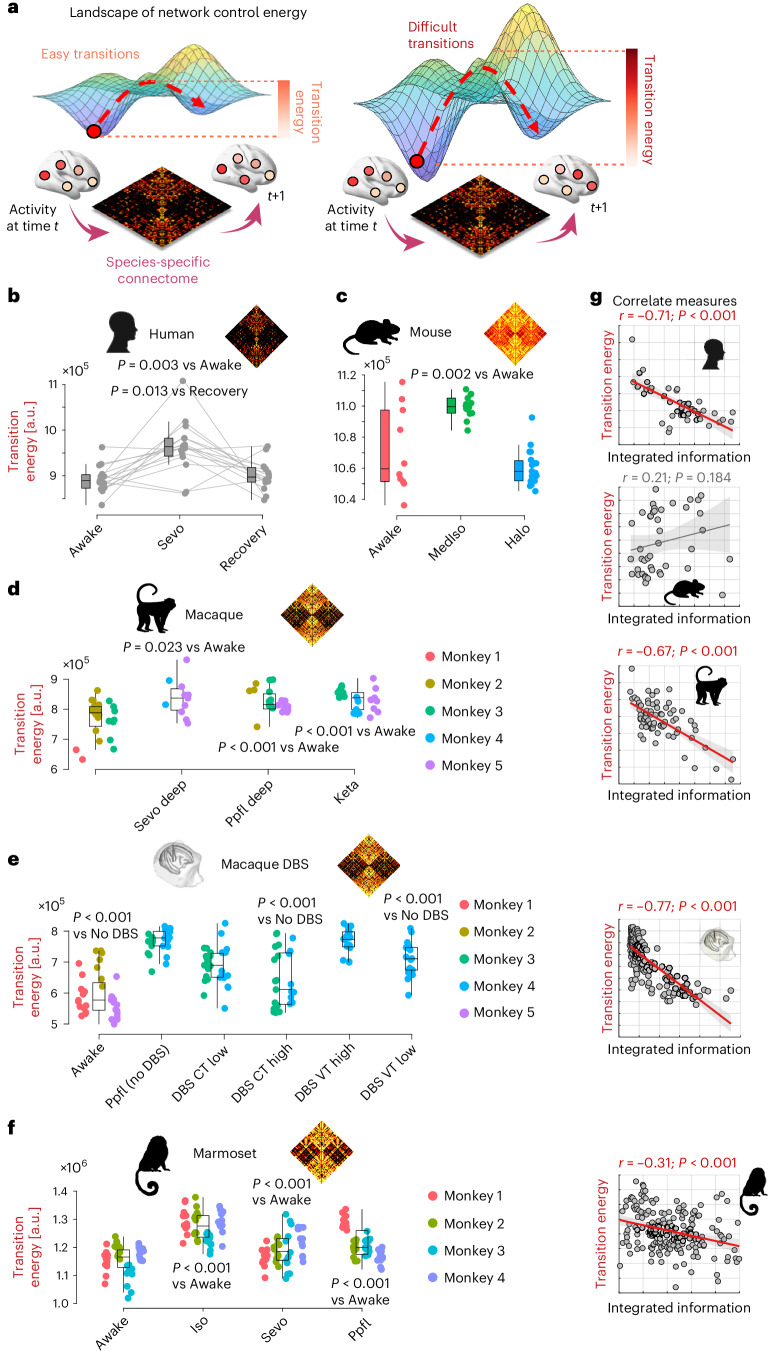
Anaesthesia and thalamic DBS exert opposite effects on the controllability of brain dynamics. **a**, Network control theory quantifies the energy (magnitude of input across time) required to transition between brain states, here defined as successive fMRI signal timepoints. Higher transition energy means that transitions are more difficult, on average, and dynamics are less controllable. **b**, Human sevoflurane data (*n* = 15), including recovery. *P* values are from two-sided paired-samples *t*-test, FDR-corrected for multiple comparisons against the awake condition, and against the recovery condition. Boxplots: central line, median; box limits, upper and lower quartiles; whiskers, 1.5× interquartile range. **c**, Mouse wakefulness (*n* = 10) versus medetomidine-isoflurane (MedIso; *n* = 14) and halothane (Halo; *n* = 19) anaesthesia data. *P* values are from two-sided independent-samples *t*-test, FDR-corrected for multiple comparisons against the awake condition. **d**, Macaque wakefulness versus anaesthesia with propofol (Ppfl), sevoflurane (Sevo) and ketamine (Keta). *N* = 24 runs from 3 animals for awake; 11 runs from 2 animals for Sevoflurane; 23 runs from 3 animals for Propofol; 22 runs from 3 animals for ketamine anaesthesia. *P* values are from linear mixed-effects models (two-sided), FDR-corrected for multiple comparisons against awake condition (see Methods). Datapoints with the same colour indicate the same animal. **e**, Macaque DBS data. CT, centro-median thalamus; VT ventrolateral thalamus. *P* values are from linear mixed-effects models (two-sided), FDR-corrected for multiple comparisons against propofol anaesthesia with no DBS (see Methods). *N* = 36 runs from 3 animals for awake; 28 runs from 2 animals for anaesthesia (DBS-off); 31 runs from 2 animals for low-amplitude centro-median thalamic DBS; 25 runs from 2 animals for high-amplitude centro-median thalamic DBS; 18 runs from 1 animal for low-amplitude ventrolateral thalamic DBS; 18 runs from 1 animal for high-amplitude ventrolateral thalamic DBS. Datapoints with the same colour indicate the same animal. **f**, Marmoset wakefulness versus anaesthesia with propofol (Ppfl), sevoflurane (Sevo) and isoflurane (Iso). *N* = 48 runs from 4 animals for each condition. *P* values are from linear mixed-effects models (two-sided), FDR-corrected for multiple comparisons against the awake condition (see Methods). In all boxplots: central line, median; box limits, upper and lower quartiles; whiskers, 1.5× interquartile range. Datapoints with the same colour indicate the same animal. See Source data for full statistical reporting. **g**, Pearson correlation (two-sided) between transition energy and integrated information across all datapoints within each dataset. Shading indicates 95% confidence intervals (CIs). Credits: human head icon (**b**,**g**) from pixabay.com; mouse icon (**c**,**g**) designed by CraftStarters.com; macaque icon (**d**,**g**) designed by Freepik.com; DBS icon (**e**,**g**) adapted from ref. ^30^, published under a CC-BY licence; marmoset icon (**f**,**g**) from pixabay.com. Source data

How can we understand the anaesthetic-induced reduction in integrated information from the brain’s past to its future? Mechanistically, the transition from past to future states of brain activity unfolds over the network of physical connections between regions: the structural connectome^35–37^. We therefore turn to ‘network control theory’, which provides a framework to understand how the network architecture of a system shapes its ability to transition between different activation states: here, patterns of regional fMRI signal^55–58^. Specifically, we can use linear control theory to determine the controllability of brain dynamics in terms of the ‘control energy’ that would be required to achieve a transition from one activation pattern to another (Fig. 4a). When it is easy to steer the system between different functional configurations, the resulting ‘energy landscape’ will be relatively flat. In contrast, a steeper landscape indicates that transitions are more effortful, corresponding to less controllable dynamics (Fig. 4a). This approach was recently used to reveal pharmacologically induced facilitation of brain state transitions^59,60^. Thus, we adopt this framework to ask: does anaesthesia induce systematic changes in the controllability of brain dynamics, that could explain why we observe a drop in integrated information?

To address this question, we capitalize on the availability of species-specific anatomical connectomes: (1) human consensus structural connectome from diffusion-weighted MRI tractography; (2) macaque structural connectivity combining diffusion-weighted MRI tractography and CoCoMac tract tracing^61^; (3) mouse structural connectome from the Allen Institute’s tract-tracing data^62^; and (4) marmoset structural connectome from in vivo diffusion-weighted MRI tractography^63^ (see Methods for details). We use these species-specific connectomes to quantify the control energy (operationalized as the squared input signal, summed across brain regions and integrated across time) required to transition between each pair of successive fMRI activation states^64^ during wakefulness and during anaesthesia.

Across species and across anaesthetics, we show that the control energy required to transition between successive timepoints of brain activity is significantly increased under anaesthesia (except for halothane in the mouse) (Fig. 4b–f). This corresponds to the brain exhibiting a steeper ‘energy landscape’ and less controllable dynamics under anaesthesia. These results from fMRI are consistent with recent modelling of electrodynamics based on the human connectome, which indicated that anaesthesia should induce a steepening of the energy landscape^65,66^.

Crucially, we can again demonstrate that this effect is not a mere by-product of the presence of anaesthetic in the bloodstream but is rather related to anaesthetic-induced suppression of responsiveness to the environment: the average transition energy is lowered again upon awakening from anaesthesia, whether due to discontinuation of the anaesthetic (human data, Fig. 4b), or induced by electrical stimulation of the central thalamus in the macaque (Fig. 4e). However, we note that this effect was to some extent also observed with low-intensity stimulation of the VT, which did not induce a corresponding restoration of behavioural arousal^30^ (we return to this point in the Discussion). Across human, both macaque datasets and marmoset, we also find a significant negative correlation between individuals’ transition energy and the integrated information in their fMRI signals (Fig. 4g). In other words, the brain’s capacity to integrate information is systematically diminished when brain dynamics are less controllable, whereupon the organism’s ability to interact with the environment is also compromised.

### Transcriptomic underpinnings of regional changes in integrated information

The analyses carried out up to this point considered integrated information at the global level, by averaging this quantity across all brain regions in the parcellation. However, to obtain further insight about the neurobiological origin of this overall reduction, we can also interrogate changes in integrated information at the regional level. We use the Network-Based Statistic (NBS)^67^ to identify pairs of regions that are significantly affected by anaesthesia, compared with either baseline wakefulness or recovery. In each species, the NBS reveals widespread significant changes in integrated information (*Φ*_R_), with the majority being reductions (that is, anaesthetic-induced dis-integration), overwhelmingly so in the case of human and macaque where the anaesthetic regimen is deepest (Supplementary Fig. 5). We also find that in each species, the majority of significant cortical changes occur between pairs of regions belonging to different functional systems (Supplementary Fig. 6), highlighting their distributed nature, in line with growing consensus that ‘anaesthesia and consciousness are network-level processes’^21^.

**Fig. 5:**
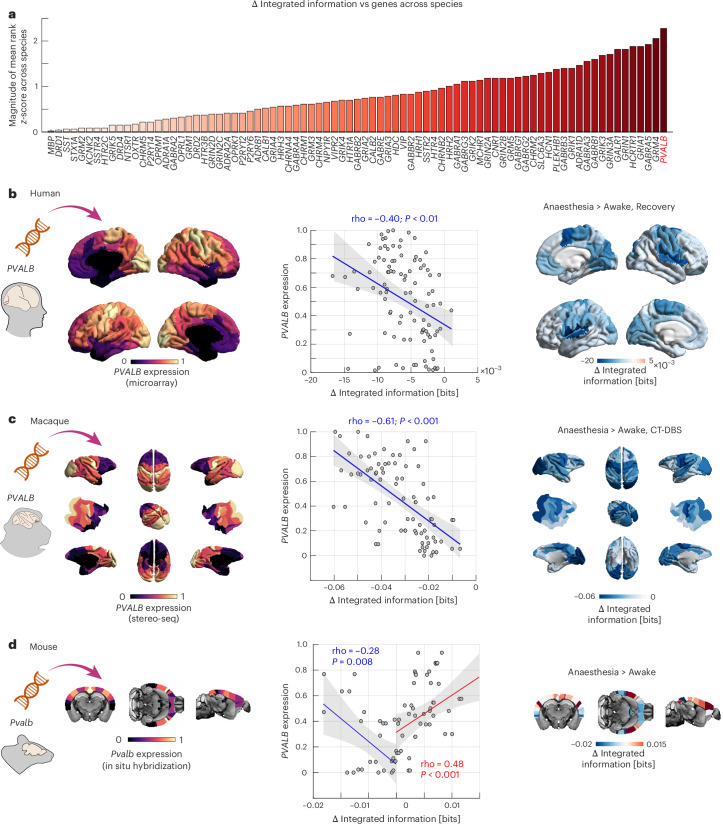
Regional loss of integrated information under anaesthesia correlates with regional *PVALB/Pvalb* gene expression across species. **a**, *PVALB*/*Pvalb* is the gene whose spatial association with regional loss of integrated information is the most consistent across species. Spatial correlations are used to compare the 81 genes within each species, ranking them from most negative to most positive. To aggregate across species, we then average the three species’ ranks. Since both positive and negative correlations are of interest, we *z*-score the ranks’ magnitude such that genes whose correlation is the most extremely ranked (in either direction) will have a higher value, indicated by a darker colour. **b**, Regionally defined *PVALB* gene expression data for the human brain are obtained from the Allen Institute for Brain Science (AIBS) transcriptomics database^39^. Across cortical regions, we observe a negative spatial correlation (Spearman’s rho, two-sided; significant against a spatial autocorrelation-preserving null distribution) between the cortical distribution of *PVALB* gene expression and the mean NBS-derived significant reduction in integrated information from comparing anaesthesia against baseline and recovery. Shading indicates 95% CI. **c**, Regionally defined *PVALB* gene expression data for the macaque brain are provided by the Brain Science Data Center of the Chinese Academy of Sciences^40^ and translated to the macaque Regional Mapping atlas by ref. ^70^. Across cortical regions, we observe a negative spatial correlation (Spearman’s rho, two-sided; significant against a spatial autocorrelation-preserving null distribution) between the cortical distribution of *PVALB* gene expression and the mean NBS-derived significant reduction in integrated information from comparing anaesthesia against baseline and recovery induced by CT deep-brain stimulation. Shading indicates 95% CI. **d**, Regionally defined *Pvalb* gene expression data for the mouse brain are obtained from the AIBS transcriptomics database^41^. Across cortical regions where integrated information is reduced under anaesthesia, we observe a negative spatial correlation (Spearman’s rho, two-sided; significant against a spatial autocorrelation-preserving null distribution) between the cortical distribution of *Pvalb* gene expression and the mean NBS-derived significant reduction in integrated information from comparing anaesthesia against baseline. Across regions where integrated information is increased under anaesthesia, we observe a positive spatial correlation (Spearman’s rho, two-sided; significant against a spatial autocorrelation-preserving null distribution) between the cortical distribution of *Pvalb* gene expression and the mean NBS-derived significant increase in integrated information from comparing anaesthesia against baseline. See Supplementary Fig. 7 for corresponding results with *SST*/*Sst* gene expression, another cell-type marker for a different class of inhibitory interneurons. Shading indicates 95% CI. Credits: silhouettes and brain icons from SciDraw.io.

We then obtain the mean value of each region’s significant changes in integrated information with the rest of the cortex, resulting in species-specific cortical maps of anaesthetic-induced changes in integrated information (Fig. 5). We see that anaesthetic-induced changes in integrated information are widely distributed across the brain, but not uniformly so, instead displaying prominent patterns of regional variation. Specifically, we find that across species, the highest prevalence of significant reductions in integrated information involves unimodal visual and somatomotor cortices (Fig. 5). We emphasize that this finding does not contradict the importance of the default mode network (DMN) and association cortex in supporting consciousness^21^, because changes in integrated information are not confined to unimodal cortices: across species, our NBS analysis also consistently finds significant reductions in integrated information in the association cortex (including anterior and posterior midline cortical regions of the human brain belonging to the DMN; Fig. 5). Likewise, the present results do not contradict early fMRI studies that showed relative preservation of functional connectivity in primary sensory networks under anaesthesia^34,68,69^, because these studies used traditional functional connectivity (that is, correlation) which reflects primarily redundant information^2,17,46^ and is therefore fundamentally different from the measure of integrated information used in the present study. Indeed, the brain-wide but unimodal-dominated spatial topography of dis-integration (reduced *Φ*_R_) observed in the present study is highly consistent with recent findings using another measure intended to quantify the balance of integration and segregation in the brain, termed integration–segregation difference (ISD)^32^. Jiang and colleagues^32^ also found distributed dis-integration throughout the human brain under anaesthesia, but with the most prominent effects in unimodal regions, similar to our own findings with reduced *Φ*_R_ (ref. ^32^). Thus, measures that are sensitive to the balance of integration and segregation (*Φ*_R_, ISD), rather than simple correlation, appear to converge on a consistent topography of anaesthesia.

The question arises: what is the neurobiological underpinning for such a consistent spatial topography? Brain regions exhibit variable cytoarchitecture and chemo-architecture, which is going to shape their susceptibility to the influence of anaesthetic compounds. Ultimately, these microarchitectural properties are shaped by each region’s unique profile of gene expression. Therefore, to identify potential neurobiological underpinnings of regional changes in integrated information, we capitalize on the availability of human and mouse transcriptomic databases from the Allen Institute for Brain Science, which provide gene expression measures across brain regions from microarray probes (human)^39^ and in situ hybridization (mouse)^41^. We complement these human and mouse databases with a third database of gene expression in the macaque cortex from stereo-seq, recently released by the Brain Science Data Center of the Chinese Academy of Sciences^40^.

We start by pursuing a data-driven approach. Specifically, we consider a list of evolutionarily conserved brain-related genes pertaining to neurotransmitter receptors and cell-type markers^70^. These human genes have orthologues in both mouse and macaque, making them comparable across species. From the genes in this list, a total of 81 genes are available and pass our quality control criteria in each of the three species (human, macaque and mouse) (Supplementary Tables 2 and 3). To obtain consistent patterns across species, we focus on cortical gene expression, for which data are available in all three species. After obtaining species-specific spatial correlations between regional loss of integrated information and each gene’s expression pattern, we use these correlations to rank genes across species to identify the most consistent pattern.

Our data-driven approach indicates that the gene with the most consistent spatial association with anaesthetic-induced reductions in integrated information is *PVALB/Pvalb* (Fig. 5a). This is noteworthy because *PVALB* (*Pvalb* in the mouse) is a marker gene that is expressed by inhibitory interneurons, which are targeted by many anaesthetics^71^ and control the onset and duration of cortical ‘down’ states^72,73^. We therefore focus more closely on *PVALB*/*Pvalb*. We show that in each of human, macaque and mouse datasets, regional anaesthetic-induced reductions in integrated information are negatively correlated with the regional density of *PVALB*/*Pvalb* expression (Fig. 5b–d). In other words, the more a region reduces its integrated information under anaesthesia, the more it expresses *PVALB*/*Pvalb*. We further use Moran spectral randomization to implement species-specific null models and confirm that the association between regional loss of integrated information and regional *PVALB*/*Pvalb* gene expression is statistically significant beyond what would be expected from random maps with preserved spatial autocorrelation^74^.

**Extended Data Fig. 2:**
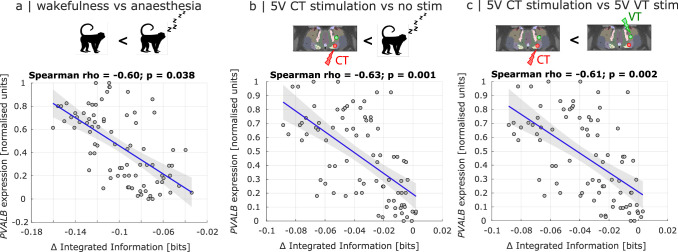
DBS-induced restoration of Integrated Information in the macaque brain mirrors the spatial topography of anaesthetic-induced disintegration and correlates with *PVALB* expression. (**a**) We observe a negative spatial correlation (Spearman’s *rho*, two-sided; significant against a spatial autocorrelation-preserving null distribution) between the cortical distribution of macaque *PVALB* gene expression, and the mean change in integrated information from comparing anaesthesia against baseline wakefulness in the macaque. Shading indicates 95% confidence intervals. (**b**) We observe a negative spatial correlation (Spearman’s *rho*, two-sided; significant against a spatial autocorrelation-preserving null distribution) between the cortical distribution of *PVALB* gene expression, and the mean change in integrated information from comparing anaesthesia against reawakening induced by CT stimulation. Shading indicates 95% confidence intervals. (**c**) We observe a negative spatial correlation (Spearman’s *rho*, two-sided; significant against a spatial autocorrelation-preserving null distribution) between the cortical distribution of *PVALB* gene expression, and the mean change in integrated information from comparing VT stimulation (which does not re-awaken the animal from anaesthesia) against CT stimulation. Shading indicates 95% confidence intervals. Credits: Macaque icon designed by Freepik.com. DBS and macaque thalamus illustrations adapted from Tasserie et al (2022), published under CC-BY license.

Specifically, in humans we observe a significant negative correlation (rho(98) = −0.40, *P* = 0.01, CI = [−0.55, −0.22]) between the cortical distribution of *PVALB* expression and the distribution of anaesthetic-induced loss of integrated information (Fig. 5b). For comparison, a significant positive correlation is observed for the marker gene for the other main class of inhibitory interneurons, *SST* (rho(98) = 0.27, *P* = 0.045, CI = [0.09, 0.45]; Supplementary Fig. 7a). For the macaque, we also find a significant negative correlation between loss of cortical integrated information and cortical expression of *PVALB* (rho(80) = −0.61, *P* < 0.001, CI = [−0.74, −0.47]; Fig. 5c). However, no corresponding significant correlation is observed with the distribution of macaque *SST* expression from stereo-seq (rho(80) = −0.01, *P* = 0.477, CI = [−0.21, 0.22]; Supplementary Fig. 7b). Notably, the restoration of integrated information induced by CT-DBS (both compared against anaesthesia without stimulation, and against stimulation of the control site, VT) mirrors the spatial topography of anaesthetic-induced dis-integration, and correlates with *PVALB* expression (Extended Data Fig. 2): the more integrated information drops under anaesthesia, the more it is restored by DBS of the centro-median thalamus. Similar to the anaesthetic-induced loss of integrated information, its regionally heterogeneous restoration also closely follows the regional expression of *PVALB* (Extended Data Fig. 2). In other words, CT-DBS counteracts the integration-suppressing effects of anaesthesia in a way that is regionally specific as a function of *PVALB* expression.

Finally, the correlation between anaesthetic-induced cortical changes in integrated information and *Pvalb* cortical gene expression in the mouse follows two opposite patterns: we observe increases in regional integrated information, as well as decreases. Across cortical regions where integrated information is reduced under anaesthesia, we observe a significant negative correlation with cortical *Pvalb* (rho(24) = −0.28, *P* = 0.008, CI = [−0.60, 0.13]), similar to that in humans and macaques. Conversely, a significant positive correlation with *Pvalb* becomes apparent for regions whose integrated information is increased under anaesthesia (rho(44) = 0.48, *P* < 0.001; CI = [0.22, 0.68]; Fig. 5d). As with the macaque, no significant correlations (for either increases or decreases in integrated information) are observed with *Sst* gene expression in the mouse after accounting for spatial autocorrelation (Supplementary Fig. 7c). Thus, in the mouse, the direction of the relationship between integrated information and *Pvalb* cortical gene expression depends on whether integrated information is increased or decreased: in both cases, more extreme changes occur for regions with higher *Pvalb* gene expression. Many differences exist between our primate and murine datasets, including acquisition parameters and the anaesthetics used; it is known that anaesthetics can differ in their effects on cerebral blood flow and fMRI signals^21,75^. However, perhaps the most likely cause for the presence of local increases in integrated information in our mouse dataset, alongside the observed reductions, is the use of a lighter anaesthetic regimen in the mouse compared with the deep anaesthesia of our human and macaque datasets. Unsurprisingly, anaesthesia exerts dose-dependent effects on brain activity and functional connectivity across species^76^. This interpretation of a role of anaesthetic depth on integrated information is further supported by our analyses with different depths of anaesthesia in the human data, showing greater loss of integrated information at greater doses of anaesthesia (Supplementary Fig. 8). Future work with mice under deeper anaesthesia will be required to provide a definitive answer to this question. Nevertheless, there is a consistent finding in all three species: in addition to the global reduction in integrated information, we consistently observe a genetic underpinning for anaesthetic-induced loss of integrated information, whereby regions that exhibit reduced integrated information under anaesthesia do so in proportion to their *PVALB*/*Pvalb* gene expression.

We also repeat our analysis across subcortical regions for which gene expression data are available in both human and mouse^39,41^. Although we find reduced integrated information for most human subcortical regions and also several mouse subcortical regions, in neither case do we find a significant correlation with *PVALB*/*Pvalb* gene expression (Supplementary Fig. 9), suggesting that this relationship between integrated information and *PVALB*/*Pvalb*, although consistent across species, may be a primarily cortical phenomenon. Inspired by recent work in humans^77^, we further investigate the specific role of the thalamus using the differential mRNA expression of *PVALB* and *CALB1* to differentiate between thalamic nuclei rich in core versus matrix cells^77,78^. We ask whether a spatial correlation exists between transcriptomically defined core–matrix architecture and anaesthetic-induced changes in integrated information. Indeed, we find that loss of integrated information is stronger for human thalamic nuclei rich in matrix cells (Supplementary Fig. 10). This prominent role of the matrix thalamus aligns with the human functional connectivity results of ref. ^77^, but also with other reports about the importance of the matrix thalamus for anaesthesia from the human and non-human primate literature^30,66,79–82^, including our own present result, that deep-brain stimulation of the macaque central thalamus reverses the loss of integrated information induced by anaesthesia (Fig. 3).

However, this human result is not observed in our mouse data. We perform the mouse analysis through two complementary strategies. First, we use the same approach as for the human data, using the relative proportion of *Calb1* to *Pvalb* mRNA expression from the Allen Mouse Brain Atlas database (Supplementary Fig. 11). Second, since mRNA is not always a good proxy for the corresponding protein in the brain^70,83^, we use immunohistochemistry data about the density of parvalbumin (the protein coded by *Pvalb*) and calbindin (the protein coded by *Calb1*) in different nuclei of the mouse thalamus, as provided by ref. ^84^. After ranking regions on the basis of the relative abundance of these proteins, we perform the same correlation between regional core–matrix prevalence and regional change in integrated information (Supplementary Fig. 12). Despite using independent datasets and modalities to define mouse thalamic architecture, both analyses converge in showing similar, non-significant correlations. On one hand, this may be due to the relatively lighter anaesthesia used in our mouse datasets (with several nuclei failing to show significant NBS-corrected changes in integrated information), compared with the deep anaesthesia of our human dataset. Future work with deeper levels of anaesthesia will be required to enable formal comparison between the two species. On the other hand, we note that thalamic cellular architecture also differs between rodents and primates. In mice, interneurons represent only 6% of thalamic neurons and are largely restricted to the visual thalamus, whereas in primates, thalamic interneurons are more prevalent (~30% of the total thalamic neuronal population) and can be found throughout the entire thalamus^85^.

### A transcriptomic gradient mediates increased control cost of brain dynamics under anaesthesia

**Fig. 6:**
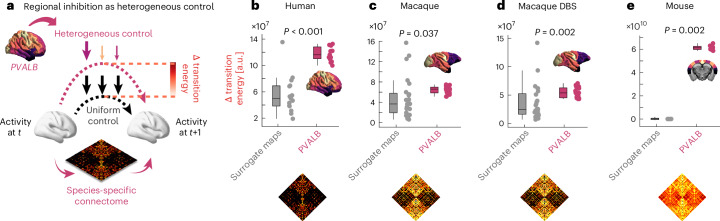
Modelling *PVALB*/*Pvalb-*mediated increase in the control cost of brain dynamics. **a**, We model regionally heterogeneous inhibition as a reduction in the amount of control energy that each region can inject into the system, proportional to that region’s *PVALB*/*Pvalb* expression. **b**–**e**, In each dataset, we compare the increase in control cost obtained with *PVALB*/*Pvalb* expression, against the control cost obtained from species-specific surrogate spatial gradients with preserved spatial autocorrelation, showing that *PVALB*/*Pvalb* induces significantly greater increases in transition costs due to its anatomical distribution. Human: *n* = 15; macaque: *n* = 24 runs from 3 animals; macaque DBS: *n* = 36 runs from 3 animals; mouse: *n* = 10. *P* values are from non-parametric paired-samples test (two-sided). For **b**–**e** boxplots: central line, median; box limits, upper and lower quartiles; whiskers, 1.5× interquartile range.

Is there also a link between *PVALB*/*Pvalb* gene expression and the observed increases in transition energy under anaesthesia? Network control theory requires a specification of a set of ‘control points’ where energy is injected into the system to induce the desired transition. For our initial quantification of transition energy, we used a uniform control strategy, whereby each brain region is given equal control. Next, we adopt a heterogeneous control strategy instead, to ask whether the increase in transition energy that we observed under anaesthesia may be driven by regional differences in *PVALB*/*Pvalb* gene expression. Given the inhibitory role of parvalbumin-positive interneurons, the main type of cell that express the *PVALB*/*Pvalb* gene, we model regionally heterogeneous inhibition as a reduction in the amount of control energy that each region can inject into the system, proportional to that region’s *PVALB*/*Pvalb* expression. This approach is analogous to a recent approach that modelled the effect of engaging the excitatory 5HT_2A_ receptor as increasing the regional amount of control energy, in proportion to each region’s receptor expression^60^ (Fig. 6a).

In other words, we seek to determine whether the specific regional distribution of *PVALB*/*Pvalb* gene expression in the mammalian cortex could correspond to especially suitable control points for inducing an increase in transition energy (that is, reduced controllability), which might then be targeted by anaesthesia. We recalculate the transition energy between each pair of successive BOLD signal patterns for the awake condition of each dataset, this time downweighting the energy injected into every region in proportion to its amount of *PVALB*/*Pvalb* gene expression (which is sigmoid normalized and thus lies between 0 and 1 in each species); that is, the more *PVALB*/*Pvalb* a region expresses, the less control input it will be able to exert on the system. Indeed, we find that the *PVALB*/*Pvalb-*weighted inputs result in higher transition energy than the uniform inputs (Supplementary Fig. 13). However, it could be argued that giving less control to some regions will result in higher control energy, regardless of their particular spatial arrangement. To demonstrate that our results are specific to the spatial distribution of *PVALB*/*Pvalb* gene expression, we therefore compare the transition energy obtained from heterogeneous control with the empirical *PVALB*/*Pvalb* regional distribution against the transition energy obtained from applying heterogeneous control with species-specific surrogate maps that preserve the *PVALB*/*Pvalb* map’s mean and spatial autocorrelation, but are otherwise random. Indeed, we find that the true regional distribution of *PVALB*/*Pvalb* gene expression is significantly better positioned to induce increases in transition energy than equivalent surrogate maps (Fig. 6). This effect is once again consistent across species: human (*n* = 15; null maps mean = 5.56 × 10^7^ (s.d. = 2.86 × 10^7^); *PVALB* mean = 1.17 × 10^8^ (s.d. = 1.06 × 10^7^), Wilcoxon sum of signed ranks = 1, *P* < 0.001; Hedge’s *g* = 2.76); macaque dataset 1 (*n* = 24; null maps mean = 4.83 × 10^7^ (s.d. = 4.04 × 10^7^); *PVALB* mean = 6.43 × 10^7^ (s.d. = 6.88 × 10^6^), Wilcoxon sum of signed ranks = 77, *P* = 0.037; Hedge’s *g* = 0.54); macaque dataset 2 (*n* = 36; null maps mean = 3.66 × 10^7^ (s.d. = 2.96 × 10^7^); *PVALB* mean = 5.37 × 10^7^ (s.d. = 8.58 × 10^6^), Wilcoxon sum of signed ranks = 137, *P* = 0.002; Hedge’s *g* = 0.77); and mouse (*n* = 10; null maps mean = 2.52 × 10^9^ (s.d. = 7.76 × 10^9^); *PVALB* mean = 6.13 × 10^10^ (s.d. = 1.90 × 10^9^), Wilcoxon sum of signed ranks = 0, *P* = 0.002; Hedge’s *g* = 9.82).

### Integrating species-specific transcriptomics and connectomics with computational modelling

Thus far, in the four mammalian species we find that anaesthesia reduces integrated information in the brain. Spatially, the best transcriptomic match for the regional reduction in integrated information is the regional expression of the *PVALB*/*Pvalb* gene, which we found to be especially suitable for inducing increases in transition energy. Such increases are indeed observed in our data. Furthermore, less controllable dynamics correlate with lower integrated information.

To go beyond correlation and obtain mechanist insights, we turn to generative computational modelling: this paradigm provides a powerful avenue to integrate multimodal data about brain network structure and neurobiology^24,42,86^. Whole-brain models, including the neurobiologically plausible dynamic mean-field (DMF) model employed here, represent regional macroscale activity in terms of two key ingredients: (1) a biophysical model of each region’s excitatory and inhibitory dynamics (see Supplementary Table 4 for model parameters); and (2) inter-regional anatomical connectivity. The model output consists of simulated BOLD signal timeseries for each region. The DMF model can be further enriched with regionally heterogeneous dynamics according to an empirical brain map of interest to interrogate its consequences for brain dynamics. Due to its multiplatform compatibility, low memory usage and high speed, we use the recently developed ‘FastDMF’ library^87^.

Here we use this model to ask how integrated information in brain dynamics is shaped by increasing regional inhibition in accordance with the empirical distribution of *PVALB*/*Pvalb* gene expression across species. For human, macaque and mouse, we develop species-specific whole-brain models informed by each species’s own structural connectome, tuned to reproduce the empirical functional connectivity dynamics (FCD) observed during wakefulness in each species^88^ (Methods; see Supplementary Table 5 for species-specific optimal working points). Next, we increase regional inhibition in proportion to each region’s normalized expression of species-specific *PVALB*/*Pvalb* gene. Note that this heterogeneous model is not directly fitted to match the anaesthesia data. Rather, its goal is to inform us about the dynamical consequences of increasing inhibition according to *PVALB*/*Pvalb* gene expression.

**Fig. 7:**
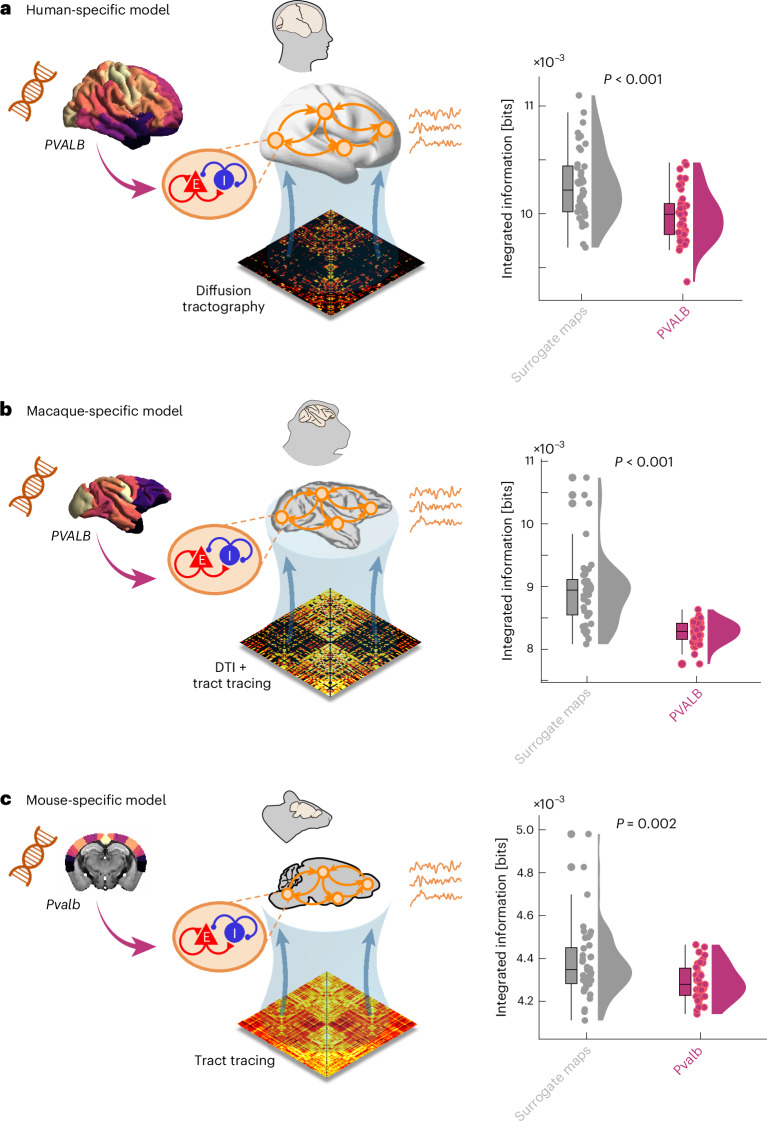
Increasing regional inhibition according to the anatomical distribution of *PVALB*/*Pvalb* expression disrupts integrated information in species-specific biophysical models. We start from models fitted to the awake functional connectivity dynamics of each species’s fMRI data. Inhibitory tone is then increased in a regionally heterogeneous manner, according to each region’s normalized *PVALB*/*Pvalb* gene expression (which is sigmoid normalized in each species and therefore bound between 0 and 1). Integrated information is then computed from the resulting simulated BOLD signals and compared against those obtained from models with regionally heterogeneous inhibition shaped by spatial autocorrelation-preserving null spatial maps. **a**, Model based on human connectome and incorporating human *PVALB* gene expression from the AIBS human transcriptomics database. Null maps (*n* = 41 simulations) mean = 1.03 × 10^−2^ (s.d. = 3.29 × 10^−4^); *PVALB* (*n* = 41 simulations) mean = 9.98 × 10^−3^ (s.d. = 2.38 × 10^−4^); *t*(80) = 4.57, *P* < 0.001 two-sided, Hedges *g* = 1.00, 95% CI [0.60, 1.45]. **b**, Model based on macaque CoCoMac connectome from DTI and tract tracing, and incorporating macaque *PVALB* gene expression from the Brain Science Data Center of the Chinese Academy of Sciences transcriptomics database. Null maps (*n* = 41 simulations) mean = 8.94 × 10^−^^3^ (s.d. = 5.85 × 10^−4^); *PVALB* (*n* = 41 simulations) mean = 8.28 × 10^−3^ (s.d. = 1.76 × 10^−4^); *t*(80) = 6.95, *P* < 0.001 two-sided, Hedges *g* = 1.52, 95% CI [1.21, 2.02]. **c**, Model based on mouse tract-tracing connectome and incorporating mouse *Pvalb* gene expression from the AIBS mouse transcriptomics database. Null maps (*n* = 41 simulations) mean = 4.38 × 10^−3^ (s.d. = 1.68 × 10^−4^); *Pvalb* (*n* = 41 simulations) mean = 4.29 × 10^−3^ (s.d. = 8.24 × 10^−5^); *t*(80) = 3.18, *P* = 0.002 two-sided, Hedges *g* = 0.70, 95% CI [0.34, 1.07]. For all boxplots: central line, median; box limits, upper and lower quartiles; whiskers, 1.5× interquartile range. *P* values are from independent-samples *t*-test. Credits: silhouettes and brain icons from SciDraw.io.

**Extended Data Fig. 3:**
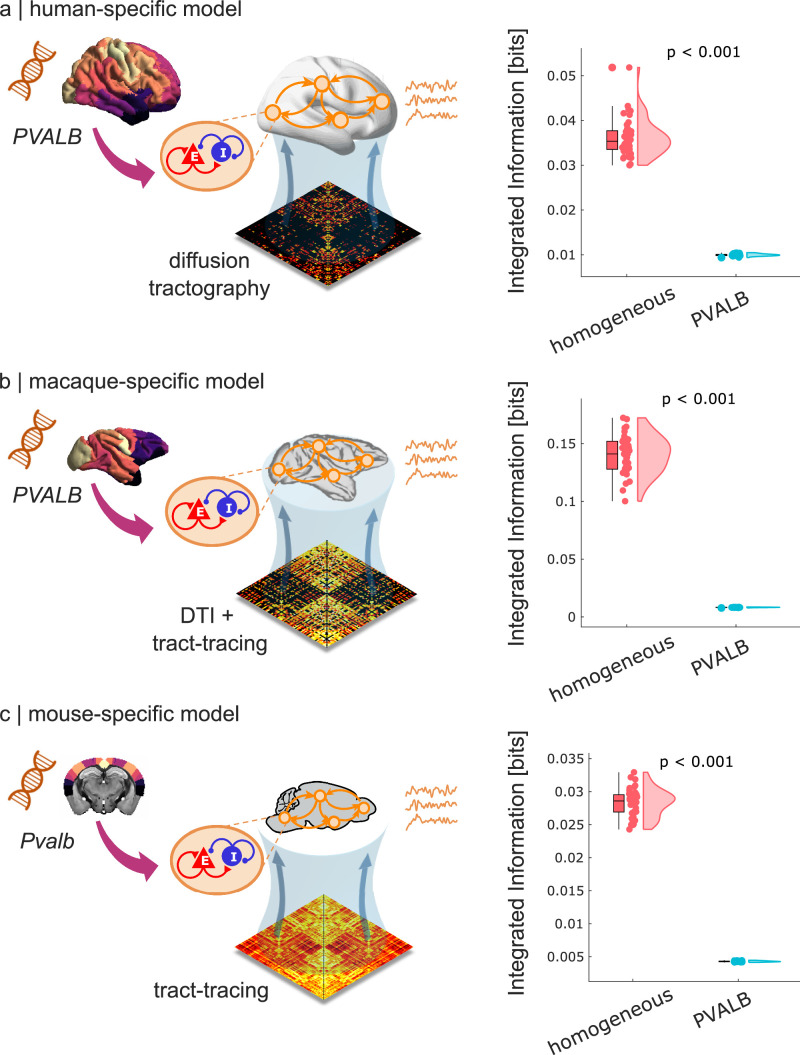
Species-specific biophysical whole-brain models exhibit significantly lower integrated information when incorporating regionally heterogeneous inhibition according to the empirical distribution of *PVALB/Pvalb* gene expression, than with homogeneous inhibition. (**a**) Model based on human connectome and incorporating human *PVALB* gene expression from the Allen Institute for Brain Science human transcriptomics database. N = 41 simulations for each condition. Box plots: central line, median; box limits, upper and lower quartiles; whiskers, 1.5× interquartile range. (**b**) Model based on macaque connectome from DTI and tract-tracing, and incorporating macaque *PVALB* gene expression from the Brain Science Data Center of the Chinese Academy of Sciences transcriptomics database. N = 41 simulations for each condition. Box plots: central line, median; box limits, upper and lower quartiles; whiskers, 1.5× interquartile range. (**c**) Model based on mouse tract-tracing connectome and incorporating mouse *Pvalb* gene expression from the Allen Institute for Brain Science mouse transcriptomics database. N = 41 simulations for each condition. Box plots: central line, median; box limits, upper and lower quartiles; whiskers, 1.5× interquartile range. All *p* < 0.001 from independent-samples t-test (two-sided).

Compared against the model with regionally homogeneous inhibition, introducing transcriptomics-informed inhibition results in simulated BOLD dynamics that exhibit significantly less integrated information (Extended Data Fig. 3). This effect is observed consistently in human, macaque and mouse. To further demonstrate that this effect is specific to the anatomical distribution of *PVALB*/*Pvalb* gene expression, we perform the same analysis using spatial autocorrelation-preserving null maps. For all three species, we find that the reduction in integrated information is significantly more pronounced when the regional heterogeneity of inhibition follows the empirical distribution of *PVALB*/*Pvalb* gene expression rather than an autocorrelation-preserving surrogate spatial gradient (Fig. 7).

**Fig. 8:**
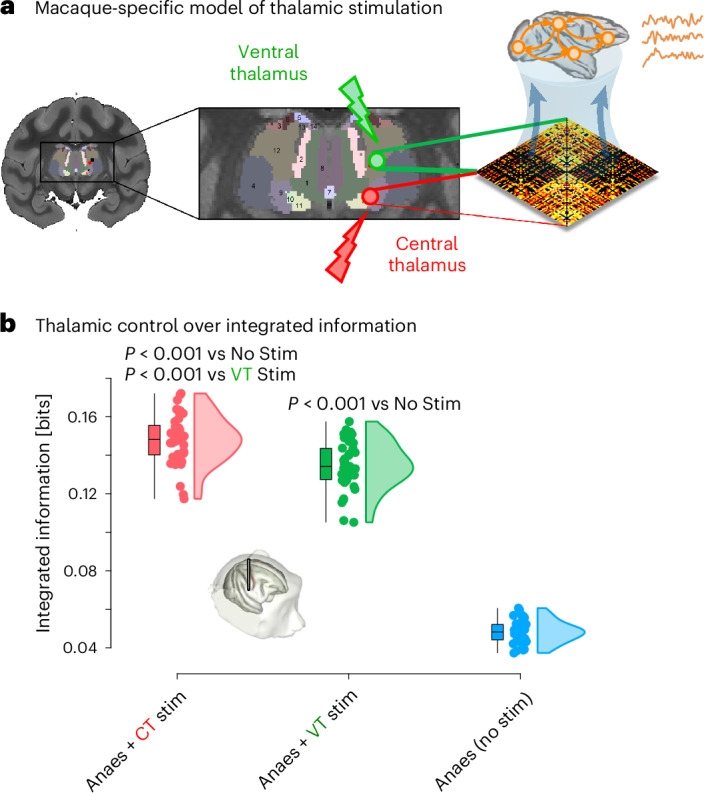
Macaque-specific biophysical model with stimulation of different thalamic nuclei reproduces the greater suitability of the central thalamus for restoring integrated information. **a**, Illustration of thalamic ROIs and their inclusion in the DMF model; adapted from ref. ^30^, published under a CC-BY licence. Models based on the macaque anatomical connectivity, fitted to the empirical anaesthesia condition, are subjected to injection of excitatory current on the basis of the structural connectivity of the central thalamus (CT, red) and ventrolateral lateral thalamus (VT, green), obtained from diffusion-weighted MRI tractography of an independent sample of macaques. **b**, Simulated CT stimulation achieves significantly integrated information in brain dynamics than both no-stimulation (CT mean = 1.48 × 10^−1^ (s.d. = 1.27 × 10^−2^); No stim mean = 4.82 × 10^−2^ (s.d. = 5.67 × 10^−3^); *t*(80) = 45.88, *P* < 0.001 from independent-samples *t*-test (two-sided), Hedges *g* = 10.04, 95% CI [8.49, 12.70]) and VT stimulation (CT mean = 1.48 × 10^−1^ (s.d. = 1.27 × 10^−2^); VT mean = 1.35 × 10^−1^ (s.d. = 1.23 × 10^−2^); *t*(80) = 4.87, *P* < 0.001 from independent-samples *t*-test (two-sided), Hedges *g* = 1.07, 95% CI [0.64, 1.58]). *N* = 41 simulations for each condition. Boxplots: central line, median; box limits, upper and lower quartiles; whiskers, 1.5× interquartile range. Credits: macaque DBS illustration adapted from ref. ^30^, published under a CC-BY licence.

Finally, we investigate whether we can obtain a model of the anaesthesia-reversing effects of thalamic stimulation observed in the macaque DBS dataset, and if so, whether the model provides a useful in silico indication of which of the two thalamic nuclei would be most suitable to stimulate for restoring integrated information. We obtain an ‘augmented’ macaque connectome, with cortico-cortical connectivity being given by the CoCoMac/DTI connectome previously used for our model^61^, plus cortical connectivity of the central and ventrolateral thalamus defined by diffusion tractography between the macaque cortex and thalamic nuclei from the SARM macaque subcortical atlas (Supplementary Methods and Fig. 8a). After tuning this model to reproduce the dynamics of the anaesthetized macaque brain (Supplementary Fig. 14), we then separately increase the intrinsic excitatory scaling of CT and VT from 1 (baseline value in the DMF model) to 3. This procedure is intended to simulate the experimental procedure of injecting input into the anaesthetized brain. We find that stimulation of both thalamic regions induces an increase in integrated information in the simulated dynamics compared to the ‘anaesthetized’ model (Fig. 8b). Remarkably, stimulation of the central thalamus region of interest (ROI) induces significantly greater recovery of integrated information than stimulation of the ventrolateral thalamus (Fig. 8b), in accordance with our empirical observations (Fig. 3). This relative advantage of CT over VT stimulation for restoring integrated information becomes more pronounced with greater levels of stimulation (Supplementary Fig. 15), also in accordance with empirical observations. Taken together, our computational modelling results show that the regional distribution of *PVALB*/*Pvalb* gene expression is especially suitable for inducing reductions in integrated information when used to tune regional inhibition. Conversely, since in our model, CT and VT differ only in terms of their empirically derived anatomical connectivity, we can infer that the connectivity profile of the CT nucleus is especially suitable for inducing increases in integrated information when used for stimulation.

### Validation and robustness

To ensure that our information-theoretic results are not unduly influenced by estimation bias, we replicate them using a debiased version of integrated information. Bias is estimated by computing the same information measure on surrogate timeseries data, constrained to have the same distribution of values for each region and preserve the instantaneous synchrony between regions (thereby preserving the functional connectivity) while destroying the past–future relationships on which integrated information is predicated. A debiased measure is obtained by subtracting the surrogate-derived quantity from the empirical quantity. We show that our results are not driven by bias, being essentially unchanged after debiasing (Supplementary Fig. 16 and Tables 6–10).

We also show results for different doses of anaesthesia available in human (Supplementary Fig. 8) and macaque (Supplementary Fig. 17) datasets. For the human dataset, we further show that consistent results are obtained with a different cortical parcellation, combining 200 cortical regions from the Schaefer atlas and 32 subcortical regions from the Tian atlas (Supplementary Fig. 8). We also show that the correlations between integrated information and transition energy remain significant in both macaque and human, when these additional data are included (Supplementary Fig. 18). We further validate our spatial correlations between integrated information and *PVALB*/*Pvalb* gene expression using (1) immunohistochemically quantified parvalbumin protein density in the macaque cortex^89^; (2) transcriptomically defined density of PV+ interneurons in the macaque cortex^40^; and (3) transcriptomically defined density of PV+ interneurons in the mouse cortex^90^ (Supplementary Fig. 19).

Although motion was actively prevented in each of the four animal datasets (see Methods) and further eliminated through denoising, we deploy three additional strategies to further mitigate any concerns about in-scanner motion affecting our measure of transition energy. First, we repeat our analyses using mean framewise displacement (FD) as a covariate of no interest in each species. We find that although some individual results become non-significant in human and marmoset, the results nonetheless remain qualitatively similar, and significant differences persist in each dataset: human, macaque multi-anaesthesia, macaque DBS, marmoset and mouse (Supplementary Tables 11–15). Second, as an alternative approach, for the human and marmoset datasets we also repeat the original analysis of transition energy, but instead of including motion as a covariate, we apply a more stringent threshold for rejection. Namely, we exclude humans with mean FD > 0.30 (3 exclusions), and we exclude marmosets with mean FD > 0.10 (5 exclusions; note that this is a conservative threshold adopted from the rodent literature). Results remain the same as in the main analyses (Supplementary Tables 16 and 17). Note that for the mouse and macaque datasets, it is superfluous to perform this analysis because all animals in all conditions are already below the stringent threshold of mean FD < 0.10. Third, we repeat the correlations between transition energy and integrated information but using partial correlation to partial out the potential confounding effects of motion (mean FD). Our results remain the same, with significant negative correlations between the two measures in each of the four primate datasets (Supplementary Fig. 20).

**Extended Data Fig. 4:**
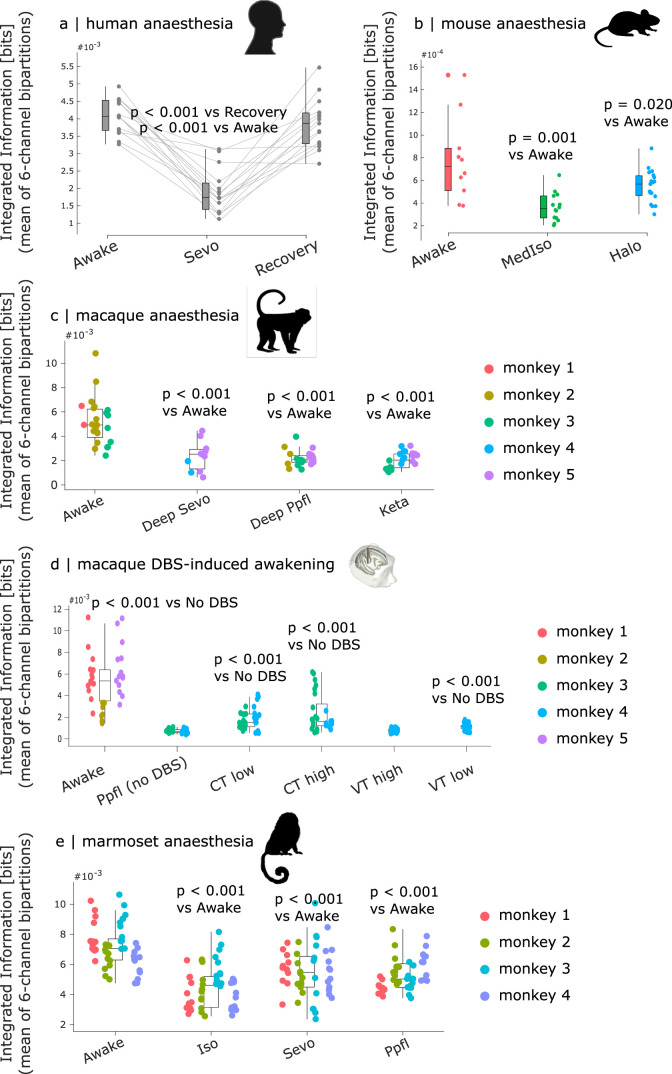
Integrated information across a bipartition of 6 channels. (**a**) Human (*n* = 15) sevoflurane anaesthesia (Sevo) versus wakefulness (p < 0.001 from two-sided paired-samples t-test, FDR-corrected for multiple comparisons against Awake condition) and recovery (p < 0.001 from two-sided paired-samples t-test, FDR-corrected for multiple comparisons against Recovery condition). Box plots: central line, median; box limits, upper and lower quartiles; whiskers, 1.5× interquartile range. (**b**) Mouse wakefulness (*n* = 10) versus medetomidine-isoflurane (MedIso; *n* = 14) and halothane (Halo; *n* = 19) anaesthesia data. P-values are from two-sided independent-samples t-test, FDR-corrected for multiple comparisons against Awake condition. Box plots: central line, median; box limits, upper and lower quartiles; whiskers, 1.5× interquartile range. (**c**) Macaque wakefulness versus anaesthesia with propofol (Ppfl), sevoflurane (Sevo), and ketamine (Keta). N = 24 runs from 3 animals for Awake; 11 runs from 2 animals for Sevoflurane; 23 runs from 3 animals for Propofol; 22 runs from 3 animals for Ketamine anaesthesia. P-values are from linear mixed effects models (two-sided), FDR-corrected for multiple comparisons against Awake condition (see Methods). Box plots: central line, median; box limits, upper and lower quartiles; whiskers, 1.5× interquartile range. (**d**) Macaque DBS stimulation. N = 36 runs from 3 animals for Awake; 28 runs from 2 animals for anaesthesia (DBS-off); 31 runs from 2 animals for low amplitude centro-median thalamic DBS; 25 runs from 2 animals for high amplitude centro-median thalamic DBS; 18 runs from 1 animal for low amplitude ventrolateral thalamic DBS; 18 runs from 1 animal for high amplitude ventrolateral thalamic DBS. P-values are from linear mixed effects models (two-sided), FDR-corrected for multiple comparisons against propofol anaesthesia with no DBS (see Methods). Box plots: central line, median; box limits, upper and lower quartiles; whiskers, 1.5× interquartile range. Data-points with the same colour indicate the same animal. (**e**) Marmoset wakefulness versus anaesthesia with isoflurane (Iso), sevoflurane (Sevo) and propofol (Ppfl). N = 48 runs from 4 animals for each condition. P-values are from linear mixed effects models (two-sided), FDR-corrected for multiple comparisons against Awake condition (see Methods). Box plots: central line, median; box limits, upper and lower quartiles; whiskers, 1.5× interquartile range. Data-points with the same colour indicate the same animal. See Source Data for full statistical reporting. Credits: Human head icon from pixabay.com. Mouse icon designed by CraftStarters.com. Macaque icon designed by Freepik.com. DBS and macaque thalamus illustrations adapted from Tasserie et al (2022), published under CC-BY license. Marmoset icon from pixabay.com. Source data

Due to the computational tractability limitations of information decomposition, for our main analyses we adopted a pairwise strategy, quantifying *Φ*_R_ between pairs of regions and then aggregating the results. However, to examine the role of integrated information generated by systems composed of more than two elements, we consider an alternative, more general strategy: instead of calculating *Φ*_R_ across pairs of regions, we calculate *Φ*_R_ across a large number of randomly sampled sets of *K* regions split into a bipartition with evenly sized parts. This approach (analysing *K* > 2 regions but divided in a bipartition) provides a suitable trade-off between considering truly higher-order information without falling victim to the combinatorial explosion of possible partitions (and elements in the information decomposition lattice). Specifically, to achieve a compromise between high order and low bias, we pick *K* = 6 and randomly sample 2,000 sets of 6 regions. Each set is divided into 2 groups of 3 regions, and we then compute the integrated information (*Φ*_R_) between the two groups of 3 channels. This method was initially proposed as a practical approach for integrated information analysis by ref. ^48^; it has already been used successfully in previous analyses using alternative measures of integrated information^91^; and more recently we have shown (using other, non-*Φ*_R_ measures of high-order information) that considering larger sets of regions beyond pairs increases measured effect sizes in empirical analyses. Indeed, we find that including a larger number of elements to consider beyond pairwise interactions does not qualitatively change our results. On the contrary, we consistently observe even stronger effect sizes, which now include false discovery rate (FDR)-corrected significant reductions in integrated information for both mouse anaesthesia conditions (Extended Data Fig. 4). This result suggests that anaesthesia may also affect the integrated information contained in beyond-pairwise interactions.

To complement our dominance analysis that directly compares *Φ*_R_ against other information-theoretic measures proposed to be relevant for consciousness, we show how each of these alternative information-theoretic quantities is reshaped by anaesthesia across all our datasets. For ease of comparison, their respective decompositions in terms of integrated information decompositions are shown in Supplementary Fig. 21. First, we consider the original proposed measure of integrated information, *Φ*_2008_, corresponding to the whole minus a naive sum of parts. This measure is therefore equivalent to the balance between integrated information (*Φ*_R_) and persistent redundancy. We show that the behaviour of *Φ*_2008_ is highly inconsistent both within and across species, and does not track loss of responsiveness (and presumably consciousness), being unchanged in anaesthetized humans or macaques, and even increased in anaesthetized mice and marmosets (Supplementary Fig. 22). Crucially, this analysis demonstrates that the possibility of *Φ*_2008_ taking negative values (paradoxical for a supposed measure of integration) is not only a theoretical concern but also a practical one: we see this happen in several of our empirical brain datasets (especially prominent in the macaque DBS dataset; Supplementary Fig. 22 and Tables 18–22).

Next we consider causal density, which attempts to quantify the overall prevalence of causal interactions as an indicator of the system’s causal complexity^50^. Specifically, causal density adopts a statistical interpretation of causality in terms of transfer entropy (information about *Y*’s future that is not provided by *Y*’s past, but only by *X*’s past). In the linear case, transfer entropy is equivalent to the Granger–Weiner notion of statistical causality^92^. Causal density is thus defined as the sum of the transfer entropies between elements. However, transfer entropy includes additional information-dynamic phenomena beyond pure unique-to-unique transfer, including synergistic effects (Supplementary Fig. 23). As a result, causal density shares several atoms with *Φ*_R_: the two pure transfer terms and all the ‘downward causation’ atoms. In a system where these were the only information-dynamic phenomena, causal density and *Φ*_R_ would therefore become equivalent. However, each measure also includes information-dynamics phenomena that the other ignores. On one hand, causal density double-counts the synergy-to-redundancy atom (being present in both transfer entropy from *X* to *Y* and transfer entropy from *Y* to *X*) and includes the two information duplication atoms (which *Φ*_R_ ignores). On the other hand, causal density does not account for any information-dynamics phenomena with synergy in the future, which instead are all included in *Φ*_R_ (ref. ^16^). Empirically, we find that causal density is substantially less sensitive to anaesthetic-induced perturbations than *Φ*_R_: in humans, causal density detects a reduction under anaesthesia but does not increase back upon recovery of responsiveness. In the mouse, the only significant difference is a paradoxical increase. In macaque and marmoset, no significant changes in causal density are observed for either anaesthesia or its DBS-induced reversal (Supplementary Fig. 23 and Tables 23–27).

Third, we consider the net balance (difference in magnitude) between the ‘pure’ flow of information from *X* to *Y* and from *Y* to *X*: this measure will be zero if the pure transfer from *X* to *Y* and from *Y* to *X* are equal. In the case of an imbalance (more transfer in one direction than the other), this measure is mathematically equivalent to the INSIDEOUT measure^93^, which was recently introduced to quantify ‘temporal irreversibility’ in linear systems. Although this ‘pure flow of information’ was not explicitly derived as a measure of consciousness, the INSIDEOUT measure has been repeatedly shown to track pharmacological and pathological perturbations of consciousness^52,53,93^, making this a relevant candidate to include. Empirically, we show that pure information flow/INSIDEOUT yields qualitatively similar results as *Φ*_R_ for tracking loss of recovery of responsiveness, being reduced in anaesthesia across species, and increasing again upon both spontaneous and DBS-induced awakening (Supplementary Fig. 24 and Tables 28–32). Indeed, the information flow/INSIDEOUT measure was also the second-best predictor of macaque behavioural arousal score in our dominance analysis, although *Φ*_R_ remains the best-performing measure, accounting for more than 2× as much variance (Fig. 3).

Finally, we repeat our dominance analysis but including an additional predictor: the traditional functional connectivity between regions which is ubiquitous in the fMRI literature. We find that our revised measure of integrated information (*Φ*_R_) remains by far the most important predictor of macaque behavioural arousal score. Alone, *Φ*_R_ accounts for almost half (46%) of all variance explained: more than 2× that of the next-best predictor (information flow/INSIDEOUT), with ~3× as much importance as either traditional functional connectivity (15%), or the original *Φ*_2008_ measure of integrated information (Supplementary Fig. 25).

Altogether, these empirical results converge with theoretical arguments in supporting the greater suitability of our revised measure of integrated information over its original formulation and alternative information-dynamic quantities for tracking consistent changes in neural dynamics under anaesthesia and their reversal with deep-brain stimulation.

## Discussion

Here we combined functional neuroimaging and causal manipulations through pharmacology and deep-brain stimulation to study macroscale integration of information in the brains of humans, macaques, marmosets and mice. Our main contributions are threefold. First, we provided evidence that breakdown of information integration is a convergent target of diverse anaesthetics across mammalian species. Second, we identified evolutionarily conserved underpinnings of anaesthetic-induced breakdown of integrated information in the connectivity and genetics of mammalian cortex. Third, we developed species-specific computational models to go beyond correlation and demonstrate how patterns of thalamic connectivity and regional inhibition mediated by *PVALB*/*Pvalb* gene expression can exert bidirectional control over neural integration of information.

By identifying changes in neural activity that coincide with the same behavioural outcome (breakdown of interaction with the environment) despite being induced by different anaesthetics, we can narrow down anaesthesia’s consciousness-suppressing effects on the brain. Across species, we provide evidence that anaesthesia reduces both integrated information and the network controllability of brain dynamics (indicated by higher transition energy). These effects are significantly correlated in all our primate datasets. A similar correlation was also found in recent analyses that applied network control theory to the structural connectomes of patients suffering from disorders of consciousness (DOC), showing that they are less suitable for supporting transitions between brain states^94^. The same patients also exhibited a correlated reduction in synergistic dynamics, a key component of integrated information^94^. Indeed, we previously showed that propofol anaesthesia and DOC both reduce integrated information between a shared set of cortical regions^45^. The same regions are also among those that exhibited reduced integrated information in the present dataset of human sevoflurane anaesthesia. Taken together, our results suggest that despite their different neurobiological origins (pharmacology and brain injury), anaesthesia and DOC-inducing brain lesions may have functionally equivalent effects: less controllable dynamics, ultimately manifesting in a failure to integrate information.

These results are relevant for potential avenues of treatment for DOC patients. On one hand, recent empirical and computational evidence indicates that suitable pharmacological intervention can also facilitate brain transitions. In particular, agonists of the serotonergic 5HT_2A_ receptor such as LSD, psilocybin and DMT induce a ‘flattening’ of the energy landscape corresponding to easier transitions^60^, and facilitatory effects have also been reported for dopaminergic agents^59^. Such pharmacologically induced facilitatory effects are the opposite of what is observed during both anaesthesia and DOC, which is encouraging because dopaminergic agents and 5HT_2A_ agonists are both being considered as potential pharmacological avenues for restoring consciousness in DOC patients^95,96^. Taken together, these studies provide empirical and computational evidence that the controllability of brain dynamics can be bidirectionally manipulated via selective pharmacological interventions.

On the other hand, the present work provides evidence that pharmacology is not the ‘only’ way to restore the controllability of brain dynamics. We also achieved the same effect via spatially selective deep-brain stimulation of the central thalamic nucleus in the macaque, which also restored behavioural arousal and integrated information. Indeed, our results converge with a growing literature indicating a role of the central thalamus for controlling consciousness and brain dynamics on both theoretical and empirical grounds^4,30,49,51,77,79–81,97–100^ (see Supplementary Discussion), including as a potential target for deep-brain stimulation in DOC patients^96,98^.

The dual causal manipulation of anaesthesia and electrical stimulation in non-human primates provides a rare opportunity to single out neural changes that specifically co-vary with the anaesthetic’s consciousness-suppressing effects. Previous work adopted this approach to show that integrated information in macaque electrodynamics is reduced by anaesthesia and sleep, and increased upon awakening induced by thalamic stimulation^80^, as are additional electrophysiological and fMRI markers of consciousness in the macaque^30,49,79,81^. Combining the specificity of deep-brain stimulation with global coverage of the entire cortex through functional MRI, the present study represents an extension of those earlier findings along multiple dimensions: (1) different neuroimaging modality (non-invasive fMRI rather than intracortical electrophysiology), allowing us to measure integrated information across the entire cortex; (2) a broader range of anaesthetics; (3) identification of transcriptomic underpinnings; and (4) crucially, generalization of the anaesthesia results to humans, mice and a different species of non-human primate (marmoset).

In addition, dominance analysis enabled us to directly compare the ability of different information-theoretic measures to track changes in behavioural arousal. On one hand, the present results consistently highlight integrated information (*Φ*_R_) as being unequivocally the most consistent information-theoretic marker of anaesthesia and its reversal, accounting for over half (51%) of the total variance explained and corroborating the susceptibility of integrative processes to loss and recovery of responsiveness. On the other hand, they also reveal net information flow (which is mathematically equivalent to the INSIDEOUT measure of temporal irreversibility^93^) as the measure that best complements *Φ*_R_ for explaining variance in behavioural arousal (being the second-best predictor). It stands to reason that the two measures should complement each other: *Φ*_R_ is symmetric and therefore insensitive to directionality of interactions in the system, whereas the net information flow is asymmetric, being high when the ‘pure’ (unique-to-unique) information transfer is greater in one direction than the other. Thus, *Φ*_R_ and net flow may complement each other because *Φ*_R_ cares about the ‘intensity’ of interactions between elements of the system (‘how much’ they interact to make an integrated whole) but not their direction, whereas net information flow cares about the ‘imbalance’ of the interactions but not their strength per se. Elucidating this newfound empirical relationship between *Φ*_R_ and net transfer/INSIDEOUT represents a promising avenue for future work.

Likewise, several other measures in the neuroscience literature are intended to reflect the balance of integration and segregation, drawing on diverse fields such as network science and dynamical systems theory^2,16,25,32,101–104^. Of particular relevance for the present results is the recently introduced measure of integration–segregation difference, defined as the difference between a network’s global efficiency and clustering coefficient^32^. When applied dynamically to different brain states obtained from human fMRI, this measure was found to be reduced under anaesthesia^32^. Thus, both *Φ*_R_ and ISD converge to indicate loss of integration during anaesthetic-induced loss of responsiveness, which is then reversed upon recovery. No formal work has yet related *Φ*_R_ and ISD, being both very recent. However, the fact that both are intended to reflect integration and segregation, and both track the effect of anaesthesia in the brain, suggests that an underlying relationship may indeed exist. In fact, an indirect relationship already exists in the literature: both ISD and *Φ*_R_ have shown empirical correlations with a third measure intended to capture integration–segregation balance: the metastability index^32,101^ (variability over time of the instantaneous synchrony), which will be high when a system is neither fully synchronized nor fully desynchronized, but rather alternates between states of high and low synchrony^102^. Nevertheless, it should be noted that *Φ*_R_ and ISD are conceptually distinct: first, ISD uses a graph-theoretic definition of integration and segregation, whereas *Φ*_R_ is based on information theory; second, integration–segregation difference is an explicit difference between the two quantities (with zero indicating perfect balance); whereas *Φ*_R_ does not subtract one from the other, but rather quantifies the presence of both. We look forward to future theoretical work that may further explore the links between these measures. Notably, similar to our present findings with *Φ*_R_, the integration–segregation difference of ref. ^32^ also displayed anaesthetic-induced reductions that were widespread across the brain but especially prominent in primary cortices (visual, somatomotor): ‘SMN and attention networks showed larger magnitudes of change compared to those of SUB, LIM and transmodal networks (FPN and DMN)’^32^. Primary visual and somatomotor regions were also the most prominent in time, being the first to disintegrate^32^. Likewise, upon recovery, ‘reintegration began with changes in the unimodal (VIS and SMN)’^32^. In other words, recent measures that consider both integration and segregation rather than simple correlation, appear to converge towards a consistent spatial topography of anaesthesia: widespread but unimodal dominated. Our transcriptomic and computational modelling results reveal a likely neurobiological reason for this spatial topography, arising from the regional prevalence of *PVALB*, the main marker of PV+ interneurons. Further, we showed that this phenomenon is under local control by the centro-median thalamus.

Our empirical observation that CT stimulation can counter the effects of anaesthesia on consciousness and information integration in fMRI signals does not, by itself, provide evidence about the underlying neurobiological mechanisms. One way to obtain such insights is via computational modelling^42^. References ^65,66^ recently used thalamocortical biophysical models based on human neuroimaging to successfully simulate the effects of anaesthesia and its reversal by stimulation of matrix (versus core) thalamus on cortical electrodynamics of the macaque, as reported by the electrophysiological experiments of ref. ^79^. Here we used a macaque-specific connectome and successfully modelled restoration of integrated information in fMRI, including the preferential role of CT over VT stimulation for restoring integrated information in the macaque. Crucially, in our model, CT and VT differ only in terms of their empirically derived patterns of anatomical connectivity with the macaque cortex, suggesting that the CT may achieve its role as local controller of global dynamics by virtue of its specific structural connectivity profile (although we note that this is likely to be only one of several convergent relevant factors, given the complex cytoarchitecture and microcircuitry of different thalamic nuclei^78,100^).

Having demonstrated that regions’ differential suitability as stimulation targets may be predicted from their structural connectivity, our computational model offers the potential for systematic screening of every region to identify alternative stimulation targets that may be even more successful than the central thalamus, or more anatomically accessible (see also Supplementary Discussion). Convergent evidence from the present work supports the translational potential of our model for DOC patients: both our empirical findings and computational models are highly consistent across human and macaque, despite being based on species-specific data. Indeed, our integration of species-specific connectomics and species-specific gene expression to model empirical observations in humans, macaques and mice constitutes one of the key advances of the present study.

Another key contribution of the present work is the identification of potential mechanisms underlying the effects of anaesthesia on macroscale information dynamics across species. Among 81 genes, the regional breakdown of integrated information induced by anaesthesia exhibits the closest cross-species correspondence with the spatial distribution of *PVALB*/*Pvalb* gene expression across humans, macaques and mice. Although regional gene expression data are not yet available at present for the marmoset cortex, we expect that future work will extend our spatial correlation results to this species, given the broader pattern of consistencies observed in the present work. *PVALB*/*Pvalb* is a cell-type marker for inhibitory interneurons, and indeed we validated a spatial association with PV+ interneurons using immunohistochemistry and cell-type deconvolution in macaque and mouse. Notably, our species-specific computational models confirmed that the spatial distribution of *PVALB*/*Pvalb* gene expression is especially suitable for shaping regional inhibition in a way that induces less controllable dynamics and less integrated information, both of which were reliably observed in our empirical data for almost every combination of species and anaesthetics.

In contrast to *PVALB*/*Pvalb*, no consistent association was observed for another prominent marker of inhibitory interneurons, *SST/Sst* gene expression. Of note, recent work in rodents showed that PV+ interneurons exert greater effect on cortical state than SST+ interneurons^72^. Crucially, the cortical pattern of parvalbumin gene expression is highly conserved from mice to humans^105^ and also from macaques to humans^70^, which may make it a plausible candidate underlying the effects of anaesthesia, which are also deeply conserved across species^19^. Admittedly, the anaesthetics employed here have widely different molecular targets, not all of which are directly known to influence inhibition or PV+ interneurons^106^. However, the brain is a complex system with intricate feedback loops and molecular cascades that are still far from completely understood. It should not be too surprising that drugs with different molecular targets at the microscale may end up exhibiting convergent effects on the same macroscale systems and physiological properties, beyond what may be apparent from in vitro studies. Indeed, ketamine shares effects with other anaesthetics such as propofol and isoflurane, not only at the macroscale (as reported here and in EEG studies^21^) but also at the mesoscale, in terms of downregulating *K*^+^/Cl^−^ co-transporter 2 (KCC2) in the ventral posteromedial nucleus of the thalamus^107^; and at the microscale, inducing decoupling between apical and basal dendritic compartments of mouse layer 5 pyramidal neurons, with a recent model showing that integrated information can be tuned by thalamic control over the coupling between apical and basal dendritic compartments of layer 5 pyramidal neurons^51^.

Crucially, apical–basal decoupling was also observed upon inactivation of the higher-order matrix thalamus^108^. Since cortical ‘down’ states during anaesthesia are controlled by thalamic drive to cortical PV+ interneurons in rodents^73^, multiple lines of evidence point to the thalamus as a potential convergence target for the results that we have reported here, which would also be in line with our empirical and computational results about the thalamus’s role in controlling state transitions. In particular, intralaminar thalamic nuclei, distinguished by ‘matrix’ cells (in opposition to ‘core’), project widely to the cortex, including the medial prefrontal and anterior cingulate cortex, as well as PV+ rich somatosensory and primary and supplementary motor cortices^100,109^. PV+ interneurons are primarily output modulating, whereas SST+ interneurons are mainly input modulating^90^. Thus, our results from empirical cortex-wide correlations and regionally heterogeneous computational models suggest that anaesthesia may affect macroscale information integration in the mammalian brain by acting more closely on output modulation than on input modulation. Future studies may investigate this possibility more explicitly by measuring changes in integrated information during optogenetic modulation of PV+ interneuron activity, with and without anaesthesia. Intriguingly, a recent study in the mouse identified PV+ interneurons as being sleep active, and selective chemogenetic manipulation further demonstrated that sleep/wake-dependent cortical ignition (the ability of local stimuli to propagate globally) is modulated by PV+ inhibition of pyramidal neurons^110^.

Our focus here was on the brain’s capacity to process environmental information, which is indexed by loss of behavioural responsiveness, a marker that is shared across species^19^ and also widely used in human clinical practice with DOC patients^111^. Indeed, the generalization of our results across four different species is a key strength of the present work. However, research with animal models comes with inevitable challenges. Since animals cannot provide subjective reports in the same way as humans, we need to rely on behavioural markers alone. Behavioural unresponsiveness is an imperfect marker of unconsciousness since it can also occur as a result of sensory disconnection or motor impairment, neither of which is the same as unconsciousness^112^. More broadly, our analysis is based on fMRI acquired in the resting state, that is, in the absence of a stimulus or a task. Responsiveness to the environment can also be evaluated in a way that bypasses behaviour, by observing the brain’s spontaneous response to naturalistic or synthetic stimuli (for example, suspenseful narratives or engaging movies^113^), as well as examining the neural effects of causal perturbations, used as a probe rather than as a means of inducing awakening^114–116^. Translating these paradigms across species will provide invaluable insights to dissociate brain and behavioural responsiveness, complementing our present identification of integrated information as a cross-species neural marker of anaesthesia.

On the theoretical side, it is important to acknowledge that integrated information decomposition is not without limitations (see ‘Measuring integrated information’ in Methods for a detailed discussion), and information decomposition remains an actively evolving field. In particular, computational tractability remains a limitation of information decomposition approaches due to combinatorial explosion in the number of terms. Therefore, here we focused on integrated information related to the dynamics of pairs of brain regions. Thus, our brain-wide estimation of information integration among pairs of regions is perhaps best viewed as a lower bound of the global integrated information that can be obtained at the level of macroscopic BOLD signals, since it neglects any additional integration between groups of three or more regions. However, we sought to mitigate this limitation by analysing a bipartition of 6 channels, which revealed consistently greater ability to detect anaesthetic-induced differences, including by identifying significant differences in both mouse anaesthesia conditions, one of which was not detected with our main analysis. Developing information decomposition approaches that scale more gracefully with system size remains an ongoing topic of active research in the field. More broadly, here we focused on ‘intrinsic’ information in the brain, but a complementary approach is to track ‘extrinsic’ task-relevant information from stimulus to behaviour across the brain^2,117–120^, although this approach is challenging to apply in the case of anaesthesia, which is operationalized by loss of behavioural responsiveness.

On the computational modelling side, it is important to bear in mind that computational models vary widely in terms of the inevitable trade-off between complexity and neurobiological detail, and where our model lies on this continuum. On one hand, our model does not incorporate individual neuron types, or layer-specific cytoarchitecture and thalamocortical connectivity^51,65,121^. On the other hand, our dynamic mean-field model represents brain regions as coupled excitatory and inhibitory neural masses, which is more biologically realistic than the binary spins of an Ising model, or the oscillators of Kuramoto and Hopf models, and therefore allowed us to intervene directly on a region’s excitation and inhibition. Choices are also inevitably required in terms of which sources of biological detail are relevant to include. If the goal were to model the detailed neurobiological mechanism of action of a specific drug with known receptor affinity, rather than our present goal of intervening directly on inhibition, then it would also be possible to implement more neurobiologically realistic receptor dynamics, for example, by adding more parameters such as regional gain scaling^121^. Instead, here we incorporate a different source of biological detail: namely, species specificity, in the form of species-specific gene expression and species-specific anatomical connectivity. Pertaining to the model of thalamic stimulation, our goals were twofold. First, to investigate whether we can obtain a species-specific model of integrated information being restored by local stimulation. Second, to investigate whether the model provides a useful in silico indication of which of the two thalamic nuclei would be most suitable to stimulate for restoring integrated information, purely on the basis of their relative connectivity profiles. Therefore, for this second objective we focused on the ‘relative’ performance of the two thalamic nuclei rather than their absolute performance. Indeed, we found greater restorative effect of CT over VT stimulation, and more so at higher stimulation levels. Both findings are in accordance with our empirical results. Nonetheless, we acknowledge that our model is not a perfect reflection of all empirical results: in particular, VT stimulation achieves greater success in our model than in real data. However, this discrepancy does not impinge on our conclusions about the relative performance between CT and VT. This partial discrepancy between the real and simulated results may arise from many factors; for example, our model does not include differential cell-type composition and gene expression of different thalamic nuclei^66,78,84,100^. Rather, we modelled the CT and VT as differing only in terms of their empirical species-specific profiles of structural connectivity. However, this relative simplicity has its own merits: it allows us to conclude that anatomical connectivity, by itself, could be a powerful predictor of a region’s suitability for stimulation aimed at restoring integrated information (without of course claiming that it is a perfect predictor, let alone the ‘only’ predictor). Ultimately, no model can ever be a perfect reflection of biological reality, and ours is no exception: as the adage goes, ‘all models are wrong’. We take the view that different models address different desiderata and provide different, complementary insights.

On the empirical side, we also acknowledge that although each of our results was repeatedly observed in multiple species and with multiple anaesthetics, the consistency was not perfect. Integrated information and the controllability of brain dynamics were also partly restored by low-intensity VT stimulation in the macaque, which did not restore behavioural arousal. We speculate that this discrepancy may occur when the neural effects (which must logically precede any behavioural ones) have not achieved sufficient prominence to translate into behaviour. Indeed, the most extreme effect of DBS on integrated information and the controllability of brain dynamics was consistently observed for high-intensity CT stimulation, concomitant with re-awakening from anaesthesia. In a similar vein, anaesthetic-induced breakdown of integrated information and increased transition energy were each absent from one of the anaesthetized mouse conditions (but note that neither effect was entirely absent in this species, and that reduced integrated information was detected in both datasets when accounting for multiple channels). Inclusion of the marmoset dataset allowed us to exclude differences in cortical gyrification as a candidate explanation for this weaker effect, since marmosets are a lissencephalic species similar to mice. As a more plausible explanation, we instead point to the fact that our mouse dataset used different anaesthetics and especially a lighter anaesthetic regimen than our dataset for the other species, such that interspecies differences may be confounded by differences between drugs^31^. In particular, medetomidine–isoflurane is an anaesthetic combination intended to minimize the effects of anaesthesia on the rodent brain (by using a smaller dose of each drug) while still suppressing motion^122^. Altogether, these dissociations open the door for future comparative studies about the specific mechanisms that enable different drugs or stimulation protocols to selectively suppress only one or the other aspect of brain function.

More broadly, we emphasize that the main focus of this work was on ‘commonalities’ rather than differences, both between drugs and between species. In this respect, broad convergence of results despite differences in species and in the anaesthetics used is an asset of our work. The same applies to the differences in acquisition, such as different temporal resolution, different scanners and magnetic field strength, and adoption of species-specific best practices for fMRI preprocessing and denoising. It is reassuring that our results display substantial consistency not only across species and anaesthetics, but also across these extraneous methodological variations. This consistency can be interpreted as an additional support for the robustness of our findings.

Overall, the results presented here offer mechanistic links between the shared neural effects of different anaesthetics and shared aspects of mammalian neurobiology. Through bidirectional causal manipulations that combine pharmacology and electrical deep-brain stimulation during functional MRI scanning, we discovered that anaesthetic-induced suppression of the mammalian brain’s ability to interact with the environment coincides with less controllable dynamics and a breakdown in the brain’s capacity to integrate information. We observed these results with different anaesthetics across humans, macaques, marmosets and mice. Integrating species-specific connectomics and transcriptomics, we further identified genetically encoded gradients of heterogeneous inhibition as potential neurobiological mechanisms underlying the shared neural effects of different anaesthetics. Going forward, our connectome-based model of DBS-induced restoration of integrated information may hold translational potential for predicting the success of stimulating different regions to re-awaken patients suffering from disorders of consciousness. Taken together, this work illuminates how regional differences in connectivity and genetically encoded circuit dynamics can be acted upon by anaesthetics and stimulation to govern information integration in the mammalian brain.

## Methods

For details of each fMRI dataset and its corresponding preprocessing and denoising procedures, see Supplementary Methods.

### Measuring integrated information

In this section, we provide a brief description of information decomposition and formulae required to compute the results. For further details, see refs. ^16,17^.

#### Partial information decomposition

We begin with Shannon’s mutual information (MI), which quantifies the interdependence between two random variables *X* and *Y*. It is calculated as

1 $$I(X;Y)=H(X)-H(X|Y)=H(X)+H(Y)-H(X,Y)$$

where *H*(*X*) stands for the Shannon entropy of a variable *X*. Above, the first equality states that the mutual information is equal to the reduction in entropy (that is, uncertainty) about *X* after *Y* is known. Put simply, the mutual information quantifies the information that one variable provides about another.

Crucially, ref. ^15^ observed that the information that two source variables *X* and *Y* give about a third target variable *Z*, *I*(*X*,*Y*; *Z*), should be decomposable in terms of different ‘types’ of information: information provided by one source but not the other (unique information); by both sources separately (redundant information); or jointly by their combination (synergistic information; Extended Data Fig. 1). Following this intuition, they developed the ‘partial information decomposition’ (PID)^15^ framework, which leads to the following fundamental decomposition:

2 $$I(X,Y;Z\,)=\mathrm{Red}(X,Y;Z\,)+\mathrm{Un}(X;Z\backslash Y\,)+\mathrm{Un}(Y;Z\backslash X\,)+\mathrm{Syn}(X,Y;Z\,).$$

Above, ‘Un’ corresponds to the unique information one source has but the other does not, ‘Red’ is the redundancy between both sources, and ‘Syn’ is their synergy: information that neither *X* nor *Y* alone can provide, but that can be obtained by considering *X* and *Y* together:

3 $$\mathrm{Syn}(X,Y;Z)=I(X,Y;Z)-(\mathrm{Red}(X,Y;Z)+\mathrm{Un}(X;Z\backslash Y)+\mathrm{Un}(Y;Z\backslash X))$$

The simplest example of a purely synergistic system is one in which *X* and *Y* are independent fair coins, and *Z* is determined by the exclusive-OR function *Z* = XOR(*X*,*Y*): that is, *Z* = 0 whenever *X* and *Y* have the same value, and *Z* = 1 otherwise. It can be shown that *X* and *Y* are both statistically independent of *Z*, which implies that neither of them provide, by themselves, information about *Z*. However, *X* and *Y* together fully determine *Z*, hence the relationship between *Z* with *X* and *Y* is purely synergistic. As another example for the case of Gaussian variables (as employed here), consider a 2-node coupled autoregressive process with two parameters: a noise correlation *c* and a coupling parameter *a*. As *c* increases, the system is flooded by ‘common noise’, making the system increasingly redundant because the common noise ‘swamps’ the signal of each node. As *a* increases, each node has a stronger influence both on the other and on the system as a whole, and we expect synergy to increase. Therefore, synergy reflects the joint contribution of parts of the system to the whole that is not driven by common noise. This can be demonstrated empirically^123^.

#### Integrated information decomposition

Dynamical systems can be studied via an information-theoretic lens by investigating how they process information through time, which can be assessed by characterizing the information flow between past and future. Formally, one can calculate the amount of information flowing from the system’s past to its future, known as ‘time-delayed mutual information’ (TDMI). Furthermore, one can use PID to decompose this information into atoms. Specifically, by denoting the past of variables as *X*_*t-τ*_ and *Y*_*t-τ*_ and treating them as information sources, and their joint future state (*X*_*t*_, *Y*_*t*_), as target, one can apply the PID framework and decompose the information flowing from past to future (TDMI) as:

4 $$\begin{array}{l}I({X}_{t-\tau },{Y}_{t-\tau };{X}_{t}{,}{Y}_{t})=\mathrm{Red}({X}_{t-\tau },{Y}_{t-\tau };{X}_{t},{Y}_{t})+\mathrm{Un}({X}_{t-\tau };{X}_{t},{Y}_{t} \backslash{Y}_{t-\tau })\\ +\mathrm{Un}({Y}_{t-\tau };{X}_{t},{Y}_{{\rm{t}}}\backslash {X}_{t-\tau })+\mathrm{Syn}({X}_{t-\tau },{Y}_{t-\tau };{X}_{t},{Y}_{t})\end{array}$$

This makes PID applicable to the dynamical systems setting, and yields a decomposition with redundant, unique and synergistic components in the past and future that can be used as a principled method to analyse information flow in neural activity^16^.

Crucially, the way in which two variables of a dynamical system encode information may itself change over time. For instance, information that was uniquely provided by one variable at one timepoint may become redundantly encoded by two in the future, or vice versa. For this reason, adopting a temporal perspective leads to an increased number of information atoms. For example, decomposing information flow between past and future of two components of a dynamical system yields not four, but rather 4 × 4 = 16 distinct information-dynamic phenomena, captured by different information atoms, each corresponding to a pair of the original four PID atoms evolving from past to future (Fig. 2a and Extended Data Fig. 1)^16^. To simplify notation, here we use the form ‘past atom → future atom’, such that for example, ‘Un*X* → Red’ refers to information that was unique to *X* in the past and is redundant in the future. To aid intuition, minimal examples of 2-element systems implementing each of the atoms are provided in Supplementary Fig. 1: each system displays only one of the 16 information-dynamic atoms.

This dynamical extension of PID was formally introduced by Mediano and Rosas under the name ‘integrated information decomposition’^16^. The 16 atoms that integrated information decomposition yields for two interdependent dynamical components can be systematically organized into a taxonomy of 6 information-dynamic phenomena (see refs. ^16,124^ for visual illustrations of this taxonomy):

- Persistence (also termed ‘storage’^16,124^): information that remains carried in the same way over time: Un*X* → Un*X*, Un*Y* → Un*Y*, Red → Red, and Syn → Syn;
- Copy (also termed ‘duplication’^16,124^): information that was not redundant in the past, but becomes redundantly available from both variables in the future; Un*X* → Red, and Un*Y* → Red;
- Pure transfer (also termed ‘migration’^16,124^): information that was uniquely present in a single variable in the past and is uniquely present in the other variable in the future; Un*X* → Un*Y*, and Un*Y* → Un*X*;
- Erasure (also termed ‘de-duplication’^16,124^): information that is pruned from duplication, such that it was redundant in the past, but is no longer redundant in the future; Red → Un*X*, and Red → Un*Y*;
- Upward causation (also termed ‘encryption’^16,124^): information that was entirely present in at least one variable in the past and becomes synergistic in the future, such that it can no longer be recovered by only considering one variable, but only by considering them both together: Un*X* → Syn, Un*Y* → Syn, and Red → Syn.
- Downward causation (also termed ‘decryption’^16,124^): information that was encoded synergistically by both variables in the past, but becomes no longer synergistic in the future, such that it is fully available from at least one of the two variables: Syn → Un*X*, Syn → Un*Y*, and Syn → Red.

Notably, this framework identifies stronger notions of redundancy, whereby information is present in both *X* and *Y* in both past and future; and synergy, whereby information is carried synergistically at all times^14,16^.

#### Minimum mutual information approximation

PID and integrated information decomposition are frameworks that formally specify the nature of the information atoms and their mutual relationships but do not fully specify how they should be estimated in practice. Hence, an important limitation of PID, which is inherited by integrated information decomposition, is the need to specify a redundancy function from which the calculation of all other atoms directly follows. A growing number of redundancy functions have been proposed, stemming from diverse literatures such as neuroscience, cryptography and game theory, and satisfying different combinations of desiderata^2,125^.

For the case of univariate Gaussian variables, several decompositions converge into the same simple form^126^. Known as ‘minimum mutual information PID’ (MMI-PID), this decomposition quantifies redundancy in terms of the minimum mutual information of each individual source with the target; synergy then becomes identified with the additional information provided by the weaker source once the stronger source is known. Crucially, this has been shown to be the most ‘conservative’ way of defining redundancy in the sense that it ensures that one does not underestimate its relevance, being an upper bound to other possible definitions^127^. An attractive feature of MMI is that it makes the redundancy only depend on the marginal distributions between individual sources and target. However, a limitation of MMI is that it calculates redundancy solely on the basis of the amount of information that each variable has about the target, which does not necessarily imply that this information comprises the same ‘content’^10^. In addition, a peculiarity of MMI is its stringent definition of unique information, which is always zero for one of the two variables under the MMI definition of redundancy. By identifying redundancy with the minimum of the MIs between each source and the target, the source whose MI is the minimum will have redundancy equal to its MI with the target. Since a source’s unique and redundant information must sum up to its MI with the target, this means that this source’s unique information will be zero. In other words, the liberal definition of redundancy under MMI is at the expense of a stringent interpretation of unique information. Relatedly, in PID, MMI is a totally monotonic function on the redundancy lattice and therefore yields a non-negative decomposition. It is worth noting that this does not hold in integrated information decomposition: the MMI double redundancy is monotonic (but not totally monotonic) on the double-redundancy lattice and can thus lead to negative-signed atoms. In practice, many PID formulations have been shown to agree in various empirical scenarios^16,128^, and consistent results have also been observed between different redundancy functions for integrated information decomposition^16,17^. Nonetheless, it should be borne in mind that they obey different theoretical desiderata and hence may be more or less suitable for specific types of analysis.

Since linear-Gaussian models are sufficiently good descriptors of functional MRI timeseries, here we adopt this MMI-PID decomposition, following our own and others’ previous applications of information decomposition to neuroscientific data^16,17,45^. Specifically, we used the Gaussian solver implemented in the JIDT toolbox (https://github.com/jlizier/jidt) to obtain TDMI, and subsequently applied the MMI decomposition as described in ref. ^16^. Moreover, following ref. ^16^, we employed the natural extension of MMI for dynamical settings, which leads to the estimation of the double-redundancy atom as:

5 $$\mathrm{Red}\to \mathrm{Red}=\min \{I({X}_{t-\tau };{X}_{{\rm{t}}}),I({X}_{t-\tau };{Y}_{t}),I({Y}_{t-\tau };{X}_{t}),I({Y}_{t-\tau };{Y}_{t})\}$$

### Information decomposition of integrated information

Through the framework of information decomposition, we can obtain an information-dynamic recipe for ‘integrated information’ in a system: information about the system’s future behaviour that becomes available when considering the system as a whole, beyond the information that is already provided by the individual parts. This whole-minus-sum information is the ‘difference that makes a difference’: information that arises from how the parts of the system interact with each other^47^.

The original formulation of ref. ^47^, which we here term *Φ*_2008_, is computed as follows:

6 $${\varPhi }_{2008}=I({X}_{t-\tau },{{\rm{Y}}}_{t-\tau };{X}_{t},{Y}_{t})-(I\left({X}_{t-\tau };{X}_{t}\right)+I\left({Y}_{t-\tau };{Y}_{t}\right))$$

However, once the original formulation from ref. ^47^ is rendered suitable for practical empirical application^48^, the resulting mathematical formulation has known shortcomings, including the fact that it can yield negative values in some cases, which are hard to interpret and seemingly paradoxical, as it does not seem plausible for a system to be ‘negatively integrated’ or an organism to have negative consciousness^48^. As a result, several alternative operationalizations of integrated information have been proposed over the years (see Supplementary Discussion for alternative development of ‘Integrated information beyond IIT 2.0’; note that our intention here is ‘not’ to test integrated information theory (in any of its versions: 2.0, 3.0 or 4.0)).

Crucially, with information decomposition, it can be formally demonstrated that *Φ*_2008_ is not a single information-dynamic quantity, but rather an aggregation of several distinct information-dynamic phenomena^16^: it contains all the synergistic information in the system, the unique information transferred from *X* to *Y* and vice versa, and importantly, the subtraction of persistent redundancy.

This insight resolves the paradox of why *Φ*_2008_ can return negative values: this will occur whenever the system is dominated by the persistent redundancy^16^.

Importantly, information decomposition also provides an explanation for why this subtraction of redundancy occurs. Based on the formula for *Φ*_2008_, the information in each of the parts is given by $I\left({X}_{t-\tau };{X}_{t}\right)$, which corresponds to information about the future of *X* that is fully provided by its past without the need for reference to any other parts of the system. In terms of information dynamics, the information provided by variable *X*, $I\left({X}_{t-\tau };{X}_{t}\right)$, is obtained by summing the four possible combinations of redundant information and *X*-unique information across past and future (recalling that redundancy is information that is present in both *X* and *Y*):

7 $$I\left({X}_{t-\tau };{X}_{{\rm{t}}}\right)=\mathrm{Un}X\to \mathrm{Un}X+\mathrm{Un}X\to \mathrm{Red}+\mathrm{Red}\to \mathrm{Un}X+\mathrm{Red}\to \mathrm{Red}$$

where the shorthand notation used in the last expression is as explained in the previous subsection. A similar decomposition can be calculated for $I\left({Y}_{t-\tau };{{\rm{Y}}}_{t}\right)$. Using these expressions, we obtain that the information contained in the sum of the parts is given by:

8 $$\begin{array}{c}I({X}_{t-\tau };{X}_{t})+I({Y}_{t-\tau };{Y}_{t})\\ =\\ \mathrm{Un}X\to \mathrm{Un}X+\mathrm{Un}X\to \mathrm{Red}+\mathrm{Red}\to \mathrm{Un}X+{\bf{Red}}\to {\bf{Red}}\\ +\\ \mathrm{Un}Y\to \mathrm{Un}Y+\mathrm{Un}Y\to \mathrm{Red}+\mathrm{Red}\to \mathrm{Un}Y+{\bf{Red}}\to {\bf{Red}}\end{array}$$

The **Red → Red** atom is therefore double counted in the formulation of the sum of the parts as $I\left({X}_{t-\tau };{X}_{t}\right)+I\left({{\rm{Y}}}_{t-\tau };{Y}_{t}\right).$ When subtracting this quantity from the total information about the system’s future that is provided by its past (that is, $I({X}_{t-\tau },\,{Y}_{t-\tau };{X}_{t},{Y}_{t})$, which is the TDMI and therefore simply the sum of all atoms, each counted once) to obtain *Φ*_2008_, the result is that we are left with a sum of all the synergy-containing atoms, plus Un*X*→ Un*Y* and Un*Y*→ Un*X*, minus **Red → Red**.

In terms of the taxonomy from integrated information decomposition, this means that *Φ*_2008_ is made up of the two atoms of pure transfer (Un*X* → Un*Y*, and Un*Y* → Un*X*), synergistic storage (Syn → Syn), encryption/upward causation (Un*X* → Syn, Un*Y* → Syn, and Red → Syn) and decryption/downward causation (Syn → Un*X*, Syn → Un*Y*, and Syn → Red), minus the persistent redundancy (Red → Red). Thus, *Φ*_2008_ double counts the persistent redundancy when computing the sum of the parts that it subtracts from the whole.

Once this issue has been identified as the source of the conceptual difficulties of *Φ*_2008_, it becomes straightforward to develop a revised ‘whole-minus-sum’ measure of integrated information (termed *Φ*_R_) that does not double count the persistent redundancy when computing the sum of the information held in the parts^16^.

The following formulations are therefore equivalent:

9 $$\begin{array}{l}{\varPhi }_{{\rm{R}}}=I\left({X}_{t-\tau },{Y}_{t-\tau };{X}_{t},{Y}_{t}\right)-\left(I\left({X}_{t-\tau };{X}_{t}\right)+I\left({Y}_{t-\tau };{Y}_{t}\right)\right)+\mathrm{Red}\to \mathrm{Red}\\ {\varPhi }_{{\rm{R}}}={\varPhi }_{2008}+\mathrm{Red}\to \mathrm{Red}\\ {\varPhi }_{{\rm{R}}}=\mathrm{Syn}\to \mathrm{Syn}+\mathrm{Syn}\to \mathrm{Un}X+\mathrm{Syn}\to \mathrm{Un}Y+\mathrm{Syn}\to \mathrm{Red}\\ \begin{array}{l}\,\,\,\,\,\,\,+\mathrm{Red}\to \mathrm{Syn}+\mathrm{Un}X\to \mathrm{Syn}+\mathrm{Un}Y\to \mathrm{Syn}+\\ \,\,\,\,\,\,\,\,\mathrm{Un}X\to \mathrm{Un}Y+\mathrm{Un}Y\to \mathrm{Un}X\end{array}\end{array}$$

Thus, we can obtain integrated information by adding all information-dynamic phenomena where information is not persistently present in the same individual variable: the sum of all synergy-containing atoms and the two transfer terms (Fig. 2a and Extended Data Fig. 1). This is the measure of integrated information that we used throughout this work (with ${\rm{Red}}\to {\rm{Red}}$ defined as in equation (4), using the minimum mutual information formalism). This measure is computationally tractable and properly reflects the original intuition of integrated information as measuring the extent to which ‘the whole is greater than the sum of its parts’, while also demonstrably yielding non-negative results even in a redundancy-dominated Gaussian system, thereby avoiding a major conceptual limitation of the original whole-minus-sum formulation of *Φ*_2008_ (ref. ^16^). In turn, this means that we can re-express *Φ*_2008_ as the balance between integrated information (*Φ*_R_) and the persistent redundancy.

To provide additional intuition about *Φ*_R_ and its relationship to the underlying network organization of a system, we provide a series of progressively more complex examples.

(1) Supplementary Fig. 1 shows the amount (in bits) of *Φ*_R_ generated by the minimal system implementing each information-dynamic atom. The results align with the theoretical decomposition of *Φ*_R_ (ref. ^16^), such that non-zero *Φ*_R_ is observed for all and only the systems whose atom is a constituent of *Φ*_R_. (2) Next, Supplementary Fig. 2 shows how much *Φ*_R_ is generated by each of the 13 possible 3-node motifs that can occur in a network, corresponding to the network’s elementary computational circuits^129^. Notably, we find higher *Φ*_R_ for motifs exhibiting recurrent connectivity, whether direct (that is, reciprocal connections between two nodes) or indirect (that is, a 3-node cycle). This link between integrated information and recurrent connectivity is intriguing, given the central role that both recurrent processing and integration of information play in many prominent theoretical accounts of consciousness^130^. (3) Finally, Supplementary Fig. 3 shows the mean amount of *Φ*_R_ between pairs of regions in a biophysically realistic network-based computational model (dynamic mean-field), whereby the wiring between regions is either the empirical human connectome from diffusion tractography, or one of several rewired network models. All null networks preserve the network size, density and weight distribution, thereby only varying in their topology: lattice, fully random topology, random but preserving the degree (number of connections) of each node, and random but preserving the degree and also the strength (sum of connections’ weights) of each node. We find that integrated information (*Φ*_R_) is lowest for the two most extreme topologies (lattice and fully random) and progressively increases as more features of human brain connectivity are introduced in the network, reaching its highest value for the empirical human connectome (Supplementary Fig. 3).

To summarize: computing integrated information (whole-minus-sum) requires computing both the information in the whole and in the sum of the parts. However, if we try to quantify the information contained in the sum of the parts by simply summing the information that can be found in part *X* without reference to *Y*, and the information that can be found in part *Y* without reference to *X*, then this ‘naïve sum of the parts’ will double count the information that is redundantly present in both *X* and *Y* across past and future (persistent redundancy). Use of this naïve sum of parts is what leads to the well-known conceptual difficulties of the original proposal for integrated information as ‘whole minus sum’ (*Φ*_2008_), including negative values for redundancy-dominated systems^16^. This issue is resolved in the revised measure of integrated information from ref. ^16^, *Φ*_R_, which does not double count the persistent redundancy when computing the sum of the information held in the parts. In turn, this means that we can re-express *Φ*_2008_ as the balance (difference) between integrated information (*Φ*_R_) and the persistent redundancy.

For our main analysis, we compute *Φ*_R_ between pairs of regions and then aggregate the results by averaging across all pairs. This approach is computationally tractable, but neglects interactions among more than two regions at a time. To achieve a compromise between high order and low bias, we adopt the same approach as^48,91^: instead of calculating *Φ*_R_ across pairs of regions, we calculate *Φ*_R_ across a large number of randomly sampled sets of *K* regions split into a bipartition with evenly-sized parts. Concretely, we pick *K* = 6 and randomly sample 2,000 sets of 6 regions. Each set is divided into 2 groups of 3 regions, and we then compute the integrated information (*Φ*_R_) between the two groups of 3 channels, finally aggregating across all sets to obtain a single estimate.

### Additional information-dynamic measures of consciousness

As described above (and shown in Extended Data Fig. 1 and Supplementary Fig. 21), the *Φ*_2008_ measure from ref. ^47^ can be obtained as the balance (difference) between integrated information (*Φ*_R_) and the persistent redundancy:

10 $${\varPhi }_{2008}=\varPhi {\rm{R}}-\mathrm{Red}\to \mathrm{Red}$$

Causal density^50,131^ corresponds to the sum of transfer entropies from *X* to *Y*, and from *Y* to *X*. Transfer entropy^132^ from *X* to *Y* is intended to quantify the information about *Y*’s future that is not provided by *Y*’s past, but only by *X*’s past. It is therefore intended to reflect the (statistical) influence of *X* on *Y*, being equivalent to the econometric measure of statistical causal influence known as Granger causality^92^, hence the name ‘causal density’ for the sum of transfer entropies. As demonstrated in ref. ^16^, transfer entropy from *X* to *Y* can be decomposed in terms of integrated information atoms as follows:

11 $${\mathrm{TE}}_{XY}=\mathrm{Syn}\to \mathrm{Red}+\mathrm{Syn}\to \mathrm{Un}Y+\mathrm{Un}X\to \mathrm{Red}+\mathrm{Un}X\to \mathrm{Un}Y$$

Thus, as shown in Supplementary Fig. 21, the causal density is computed as

12 $$\begin{array}{l}\mathrm{CD}=2(\mathrm{Syn}\to \mathrm{Red})+\mathrm{Syn}\to \mathrm{Un}Y+\mathrm{Un}X\to \mathrm{Red}+\mathrm{Un}X\to \mathrm{Un}Y\\ +\mathrm{Syn}\to \mathrm{Un}X+\mathrm{Un}Y\to \mathrm{Red}+\mathrm{Un}Y\to \mathrm{Un}X\end{array}$$

The net information flow can be obtained from information decomposition of the fMRI signal timeseries between pairs of regions, as the sum of the absolute difference between information that was in region *X* and is then in region *Y* (unique *X* to unique *Y*), and the information that was in region *Y* and is then in region *X* (unique *Y* to unique *X*) (Supplementary Fig. 21). These two information-dynamic terms correspond to the ‘pure transfer’ between *X* and *Y* (disregarding any synergistic phenomena or duplication of information). Information flow is zero when the two terms are equal in sign and magnitude, that is, *X* and *Y* are in balance. A positive value reflects the presence of a net flow of information between the two. Presence of a net flow of information indicates an asymmetry in the system, and therefore information flow as defined here is mathematically related to the notion of ‘temporal irreversibility’, as quantified by the recently introduced INSIDEOUT measure^93^.

As with integrated information, for all information-theoretic measures, a single overall value for the entire brain is obtained by averaging across all pairs of regions. This pairwise approach is necessary because information decomposition scales super-exponentially with the number of elements in the system; overcoming this limitation remains an area of active research.

### Network control energy

Network control theory allows us to probe the constraints of white-matter connectivity on dynamic brain activity and to calculate the minimum energy required for the brain to transition from one activation pattern to another^58,60^. The model of brain dynamics used for network controllability analysis is based on extensive previous work demonstrating its wide applicability in health and disease^55–57,60^. In effect, there exists substantial evidence that linear models provide an adequate description of the brain dynamics measured with fMRI, such that more complicated nonlinear models only capture little additional variance. On the basis of this literature and the well-known tractability of linear models, here we follow previous work on network control theory applications to structural brain networks^55^.

While this procedure has been detailed elsewhere^60^, we briefly summarize it here, following the same wording as in our previous work. For each species, we obtained an *N* × *N* structural connectome *A* as described above, where *N* is the number of regions (100 for human, 82 for macaque, 70 for marmoset and 162 for mouse). We then employed a linear time-invariant model:

13 $${\dot{\bf{{x}}}}(t)={A{\bf{x}}}(t)+{B{\bf{u}}}(t)$$

where **x** is a vector of length *N* containing the regional activity at time *t*. *B* is an *N* × *N* matrix that contains the control input weights, and is otherwise known as the control strategy. Here, *B* is the identity matrix, reflecting uniform control from all regions. To compute the minimum control energy required to drive the system (network) from an initial activity pattern (*x*_0_) to a final activity pattern (*x*_f_) over some finite amount of time (*T*), we minimize the inputs (*u*(*t*)) subject to equation (13):

14 $${\bf{u}}(t)^{*} =\min {\int }_{0}^{T}{\bf{u}}^{{{\top }}}(t){\bf{u}}(t){dt}$$

where *T* is the time horizon that specifies the time over which input to the system is allowed. Here, a common choice of *T* = 1 was used. The minimum control energy for a single brain region is then:

15 $${E}_{i}^{* }={\int }_{0}^{T}{{||}{\bf{u}(t)}_{i}^{* }{||}}_{2}^{2}{dt}$$

Finally, the global minimum control energy for a transition sums over each node:

16 $${E}_{\min }=\mathop{\sum }\limits_{i=1}^{N}{E}_{i}^{* }$$

This quantity (*E*_min_) was calculated for each pair of initial *x*_0_ and final *x*_f_ brain states (that is, adjacent fMRI signal volumes in each individual’s fMRI scans) and then averaged across the whole timeseries duration to obtain an overall estimate of transition energy under each condition.

Network control theory requires a specification of a set of ‘control points’ where energy is injected into the system to induce the desired transition: this control strategy is formalized in the matrix of control input weights *B*. For our initial quantification of transition energy, we used a uniform control strategy whereby equal inputs are provided at each brain region, such that *B* is the identify matrix. To model the potential role of regionally heterogeneous inhibition, we reduce the amount of control energy that each region can inject into the system, proportional to that region’s *PVALB/Pvalb* normalized gene expression. Concretely, this is implemented as subtracting from the identity matrix of uniform control input weights *B*, a diagonal matrix where each entry is the normalized gene expression (whose values lie in the range [0,1]). Thus, entries along the diagonal of the heterogeneous control matrix *B* are not all 1s anymore but lie in the range [0,1]. This approach for modulating the control strategy is analogous to a recent approach that modelled the effect of engaging the excitatory 5HT_2A_ receptor as increasing the regional amount of control energy in proportion to each region’s receptor expression^60^.

### Whole-brain computational modelling

The whole-brain computational modelling framework, as used in our previous work^88,94^ and implemented in the ‘FastDMF’ library^87^, is described in detail in the Supplementary Methods. Below we describe the main innovations specific to the present work.

#### Model with regionally heterogeneous inhibition

To interrogate the effect of providing additional inhibition in a regionally heterogeneous way as a simplified model of what might occur under anaesthesia, we increase the value of local inhibitory input according to the value of each region’s normalized *PVALB/Pvalb* gene expression quantified from species-specific transcriptomics. Specifically, regional inhibitory input was increased by its original value (0.7 for every region) multiplied by the region’s normalized gene expression. Since in each species, the gene expression values are sigmoid normalized and therefore lie in the range [0,1], the values of inhibition in the regionally heterogeneous models range from 0.7 (original value) to 1.4 (that is, doubled inhibition). The DMF model was then run as described above, and the corresponding functional measures were computed. To dissociate the effects of a heterogeneous distribution of inhibition from those of neuroanatomy, we also repeated the above process with uncorrelated surrogate versions of the species-specific gene expression maps (see ‘Null models’ section of the Supplementary Methods).

#### Modelling electrical stimulation of thalamic nuclei

##### Stimulation protocol

Based on the augmented macaque thalamocortical connectome, the DMF model was used to simulate 41 instances of BOLD timeseries using the *G* parameter that best reproduced the empirical dynamics observed in the macaque under anaesthesia. This corresponds to simulation of the anaesthetized condition. To simulate DBS-induced awakening, thalamic stimulation was modelled by increasing the excitatory scaling of the external input current to the CT or VT from 1 (baseline value) to 3, and then generating 41 instances of BOLD signals for each condition. In Supplementary Fig. 15, we also report results for alternative values of excitatory scaling (1.5× and 2×).

### Statistical reporting

See Supplementary Methods for full statistical reporting information.

### Reporting summary

Further information on research design is available in the Nature Portfolio Reporting Summary linked to this article.

## Supplementary information

Supplementary Information Supplementary Methods, Discussion, Figs. 1–25 and Tables 1–5.

Reporting Summary

Supplementary Table Supplementary Tables 6–10.

Supplementary Tables Supplementary Tables 11–15.

Supplementary Table Supplementary Tables 16 and 17.

Supplementary Table Supplementary Tables 18–22.

Supplementary Table Supplementary Tables 23–27.

Supplementary Table Supplementary Tables 28–32.

## Source data

Source Data Fig. 2 Statistical source data.

Source Data Fig. 3 Statistical source data.

Source Data Fig. 4 Statistical source data.

Source Data Extended Data Fig. 4 Statistical source data.

## Data Availability

For the human sevoflurane dataset, data are available from author D.G. through academic collaboration. For the macaque multi-anaesthesia dataset, raw data are available for access from B.J. through academic collaboration. For the macaque DBS dataset, raw data are available for access from B.J. through academic collaboration. For the mouse dataset, data are available from A.G. The marmoset fMRI data are available from K.M. through academic collaboration. The HCP DWI data in SRC format are available online (http://brain.labsolver.org/diffusion-mri-data/hcp-dmri-data). The macaque structural connectome is available on Zenodo at 10.5281/zenodo.1471588. The CoCoMac database is also available online at http://cocomac.g-node.org/main/index.php?. Preprocessed macaque dMRI data in DSI Studio format are available on Zenodo (10.5281/zenodo.6321168). The mouse connectome is available from A.G. The marmoset structural connectivity data are available online at 10.24475/bminds.mri.thj.4624. Human gene expression data^39^ are available from the Allen Human Brain Atlas at http://human.brain-map.org/static/download. Mouse gene expression data^41^ are available at https://mouse.brain-map.org/. Macaque cortical gene expression data from ref. ^40^ are available at https://macaque.digital-brain.cn/spatial-omics. The dataset is provided by Brain Science Data Center, Chinese Academy of Sciences (https://braindatacenter.cn/). The macaque gene expression data resampled to the Regional Mapping atlas are available at https://github.com/netneurolab/luppi-genes-receptors-macaque. Mouse regional PV+ neuron count data are from Table S3 in ref. ^90^. Macaque parvalbumin density data from immunohistochemistry for several regions of the macaque cortex are available in the Supplementary Materials of ref. ^89^. Immunohistochemically derived measurements of the relative prevalence of calbindin-positive and parvalbumin-positive neurons in different thalamic nuclei are available from the Supplementary Material of ref. ^84^. Source data are provided with this paper.

## Code availability

Analysis was performed in MTLAB v.2019a and 2024b, and Python 3.11. The Python processing for PreClinical data pipeline, Pypreclin v.1.0.1, is freely available at https://github.com/neurospin/pypreclin. FMRIB Software Library (FSL) is freely available online (http://www.fmrib.ox.ac.uk/fsl/; version accessed 4 February 2018). The CONN toolbox v.17f is freely available at http://www.nitrc.org/projects/conn/. DSI Studio (v.2022) is freely available at https://dsi-studio.labsolver.org/. The ‘abagen’ toolbox (v.0.1.4) is available at https://github.com/rmarkello/abagen. The RheMap toolbox (v.1.4) is available at 10.5281/zenodo.3668510. The BrainSpace toolbox for generation of Moran spectral surrogates is available at https://brainspace.readthedocs.io/en/latest/. The Python toolbox for Dominance Analysis is freely available at https://github.com/dominance-analysis/dominance-analysis. The Brain Connectivity Toolbox is available online at https://sites.google.com/site/bctnet/. The JIDT toolbox is available at https://github.com/jlizier/jidt. FastDMF toolbox whole-brain modelling is available at https://www.gitlab.com/concog/fastdmf. MATLAB/Octave and Python code to compute measures of integrated information decomposition of timeseries with the Gaussian MMI solver is available at https://github.com/Imperial-MIND-lab/integrated-info-decomp.
