# Supplementary material for: Convergent transcriptomic and connectomic controllers of information integration and its anaesthetic breakdown across mammalian brains
Source: Nat Hum Behav. 2026 Jan 28;10(4):777–802. doi: 10.1038/s41562-025-02381-5 (PMC13121024; doi:10.1038/s41562-025-02381-5)
Supplement: Supplementary file 1 — Supplementary Methods, Discussion, Figs. 1–25 and Tables 1–5. [file 41562_2025_2381_MOESM1_ESM.pdf]

nature human behaviour

Supplementary information

https://doi.org/10.1038/s41562-025-02381-5

Convergent transcriptomic and connectomic controllers of information integration and its anaesthetic breakdown across mammalian brains

In the format provided by the authors and unedited

Supplementary Information for

Convergent Transcriptomic and Connectomic Controllers of Information Integration and its Anaesthetic Breakdown Across Mammalian Brains

Luppi, Uhrig, Tasserie, *et al.*

Supplementary Methods

Human Sevoflurane Dataset

The human sevoflurane fMRI data used here have been previously reported (Luppi et al., 2023b; Ranft et al., 2016a). For clarity and consistency of reporting, where possible we use the same wording as in previous reports (Luppi et al., 2023b; Ranft et al., 2016a).

Recruitment and ethics

The data included here have been published before (Luppi et al., 2023b; Ranft et al., 2016a) and we refer the reader to the original publication for details (Ranft et al., 2016a). The ethics committee of the medical school of the Technische Universität München (München, Germany) approved the current study, which was conducted in accordance with the Declaration of Helsinki. Written informed consent was obtained from volunteers at least 48 h before the study session. Twenty healthy adult men (20 to 36 years of age; mean, 26 years) were recruited through campus notices and personal contact, and compensated for their participation in the study.

Before inclusion in the study, detailed information was provided about the protocol and risks, and medical history was reviewed to assess any previous neurologic or psychiatric disorder. A focused physical examination was performed, and a resting electrocardiogram was recorded. Further exclusion criteria were the following: physical status other than American Society of Anesthesiologists physical status I, chronic intake of medication or drugs, hardness of hearing or deafness, absence of fluency in German, known or suspected disposition to malignant hyperthermia, acute hepatic porphyria, history of halothane hepatitis, obesity with a body mass index more than 30 kg/m2, gastrointestinal disorders with a disposition for gastroesophageal regurgitation, known or suspected difficult airway, and presence of metal implants. Data acquisition took place between June and December 2013. No statistical methods were used to pre-determine sample sizes but our sample sizes are similar to those reported in previous publications (Ranft et al., 2016b). Since anaesthesia is identified by behavioural loss of responsiveness, no blinding was possible.

Study protocol

Sevoflurane concentrations were chosen so that subjects tolerated artificial ventilation (reached at 2.0 vol%) and that burst-suppression (BS) was reached in all participants (around 4.4 vol%). To make group comparisons feasible, an intermediate concentration of 3.0 vol% was also used. In the MRI scanner, volunteers were in a resting state with eyes closed for 700s. Since EEG data were simultaneously acquired during MRI scanning (Ranft et al., 2016a) (though they are not analysed in the present study), visual online inspection of the EEG was used to verify that participants did not fall asleep during the pre-anaesthesia baseline scan. Sevoflurane mixed with oxygen was administered via a tight-fitting facemask using an fMRI-

compatible anaesthesia machine (Fabius Tiro, Dräger, Germany). Standard American Society of Anesthesiologists monitoring was performed: concentrations of sevoflurane, oxygen and carbon dioxide, were monitored using a cardiorespiratory monitor (DatexaS/3, General electric, USA). After administering an end-tidal sevoflurane concentration (etSev) of 0.4 vol% for 5 min, sevoflurane concentration was increased in a stepwise fashion by 0.2 vol% every 3 min until the participant became unconscious, as judged by the loss of responsiveness (LOR) to the repeatedly spoken command "squeeze my hand" two consecutive times. Sevoflurane concentration was then increased to reach an end-tidal concentration of approximately 3 vol%. When clinically indicated, ventilation was managed by the physician and a laryngeal mask suitable for fMRI (I-gel, Intersurgical, United Kingdom) was inserted. The fraction of inspired oxygen was then set at 0.8, and mechanical ventilation was adjusted to maintain end-tidal carbon dioxide at steady concentrations of 33 ± 1.71 mmHg during BS, 34 ± 1.12 mmHg during 3 vol%, and 33 ± 1.49 mmHg during 2 vol% (throughout this article, mean ± SD). Norepinephrine was given by continuous infusion (0.1 ± 0.01 $\mu g \cdot kg^{-1} \cdot min^{-1}$) through an intravenous catheter in a vein on the dorsum of the hand, to maintain the mean arterial blood pressure close to baseline values (baseline, 96 ± 9.36 mmHg; BS, 88 ± 7.55 mmHg; 3 vol%, 88 ± 8.4 mmHg; 2 vol%, 89 ± 9.37 mmHg; follow-up, 98 ± 9.41 mmHg). After insertion of the laryngeal mask airway, sevoflurane concentration was gradually increased until the EEG showed burst-suppression with suppression periods of at least 1,000 ms and about 50% suppression of electrical activity (reached at 4.34 ± 0.22 vol%), which is characteristic of deep anaesthesia. At that point, another 700s of electroencephalogram and fMRI was recorded. Further 700s of data were acquired at steady end-tidal sevoflurane concentrations of 3 and 2 vol%, respectively, each after an equilibration time of 15 min. In a final step, etSev was reduced to two times the concentration at LOR. However, most of the subjects moved or did not tolerate the laryngeal mask any more under this condition: therefore, this stage was not included in the analysis (Ranft et al., 2016a).

Sevoflurane administration was then terminated, and the scanner table was slid out of the MRI scanner to monitor post-anaesthetic recovery. The volunteer was manually ventilated until spontaneous ventilation returned. The laryngeal mask was removed as soon as the patient opened his mouth on command. The physician regularly asked the volunteer to squeeze their hand: recovery of responsiveness was noted to occur as soon as the command was followed. Fifteen minutes after the time of recovery of responsiveness, the Brice interview was administered to assess for awareness during sevoflurane exposure; the interview was repeated on the phone the next day. After a total of 45 min of recovery time, another resting-state combined fMRI-EEG scan was acquired (with eyes closed, as for the baseline scan). When participants were alert, oriented, cooperative, and physiologically stable, they were taken home by a family member or a friend appointed in advance.

MRI data acquisition

Although the original study acquired both functional MRI (fMRI) and electroencephalographic (EEG) data, in the present work we only considered the fMRI data. Data acquisition was carried out on a 3-Tesla magnetic resonance imaging scanner (Achieva Quasar Dual 3.0T 16CH, The Netherlands) with an eight-channel, phased-array head coil. The data were collected using a gradient echo planar imaging sequence (echo time = 30 ms, repetition time (TR) = 1.838 s, flip angle = 75°, field of view = 220 × 220 mm^2, matrix = 72 ×

72, 32 slices, slice thickness = 3 mm, and 1 mm interslice gap; 700-s acquisition time, resulting in 350 functional volumes). The anatomical scan was acquired before the functional scan using a T1-weighted MPRAGE sequence with 240 × 240 × 170 voxels (1×1×1 mm voxel size) covering the whole brain. A total of 16 volunteers completed the full protocol and were included in our analyses; one subject was excluded due to high motion, leaving N=15 for analysis. In our main results we used fMRI data from the Awake, 3% vol, and Recovery scans; results for 2% vol and burst-suppression are provided in Figure S8.

Functional MRI preprocessing, denoising, and timeseries extraction

We applied a standard preprocessing pipeline, which we have previously employed with pharmaco-MRI datasets of anaesthesia, demonstrating its suitability for the present analysis. Preprocessing was performed using the CONN toolbox, version 17f (CONN; http://www.nitrc.org/projects/conn)(Whitfield-Gabrieli and Nieto-Castanon, 2012) based on Statistical Parametric Mapping 12 (http://www.fil.ion.ucl.ac.uk/spm), implemented in MATLAB 2016a. The pipeline involved the following steps: removal of the first 10s, to achieve steady-state magnetization; motion correction; slice-timing correction; identification of outlier volumes for subsequent scrubbing by means of the quality assurance/artifact rejection software *art* (http://www.nitrc.org/projects/artifact_detect); normalisation to Montreal Neurological Institute (MNI-152) standard space (2 mm isotropic resampling resolution), using the segmented grey matter image from each volunteer's T1-weighted anatomical image, together with an *a priori* grey matter template.

Denoising was also performed using the CONN toolbox, using the same approach as in our previous publications with pharmaco-MRI datasets (Luppi et al., 2023b, 2019), which has also been adopted with similar pharmaco-MRI datasets in publications by independent groups (Müller et al., 2021). Pharmacological agents can induce alterations in physiological parameters (heart rate, breathing rate, motion) or neurovascular coupling (Power et al., 2012). The anatomical CompCor (aCompCor) method removes physiological fluctuations by extracting principal components from regions unlikely to be modulated by neural activity; these components are then included as nuisance regressors (Behzadi Y et al., 2007). Following this approach, five principal components were extracted from white matter and cerebrospinal fluid signals (using individual tissue masks obtained from the T1-weighted structural MRI images) (Müller et al., 2021); and regressed out from the functional data together with six subject-specific realignment parameters (three translations and three rotations) as well as their first-order temporal derivatives; followed by scrubbing of outliers identified by ART, using Ordinary Least Squares regression (Whitfield-Gabrieli and Nieto-Castanon, 2012). Finally, the denoised FMRI signal timeseries were linearly detrended and band-pass filtered to eliminate both low-frequency drift effects and high-frequency noise, thus retaining frequencies between 0.008 and 0.09 Hz.

Functional MRI signals were parcellated into 100 cortical regions from the widely used Schaefer cortical atlas. We replicate the results with a different parcellation of the human brain (Luppi and Stamatakis, 2021), comprising 200 cortical regions from the Schaefer atlas as well as 32 subcortical regions from the Tian atlas (Tian et al., 2020) (Figure S8).

Macaque Multi-Anaesthesia and DBS datasets

The macaque fMRI datasets used here have been previously reported (Luppi et al., 2024b; Tasserie et al., 2022; Uhrig et al., 2018). For clarity and consistency of reporting, where possible we use the same wording as in previous reports (Luppi et al., 2024b; Tasserie et al., 2022; Uhrig et al., 2018).

Animals and ethics

For the macaque Multi-Anaesthesia dataset, five rhesus macaques were included for analyses (Macaca mulatta, one male, monkey J, and four females, monkey A, K, Ki, and R, 5-8 kg, 8-12 yr of age), in a total of six different arousal conditions: awake state, deep ketamine, light propofol, deep propofol, light sevoflurane, and deep sevoflurane anaesthesia. Three monkeys were used for each condition: awake state (monkeys A, K, and J), ketamine (monkeys K, R and Ki), propofol (monkeys K, R, and J), sevoflurane (monkeys Ki, R, and J). Each monkey had fMRI resting-state acquisitions on different days and several monkeys were scanned in more than one experimental condition. Sex was not considered in this study. Because of the small sample sizes, the sex balance per group could not be secured. All procedures are in agreement with the European Convention for the Protection of Vertebrate Animals used for Experimental and Other Scientific Purposes (Directive 2010/63/EU) and the National Institutes of Health's Guide for the Care and Use of Laboratory Animals. Animal studies were approved by the institutional Ethical Committee (Commissariat à l'Energie atomique et aux Énergies alternatives; France; protocols CETEA \#10-003 and 12-086). Additional details of the acquisitions can be found in the original publications (Barttfeld et al., 2015; Uhrig et al., 2018).

For the macaque DBS dataset, five male rhesus macaques (Macaca mulatta, 9 to 17 years and 7.5 to 9.1 kg) were included, three for the awake (non-DBS) experiments (monkeys B, J, and Y) and two for the DBS experiments (monkeys N and T). Only males were included in the DBS dataset in order to avoid the menstrual cycle and hormone variations. All procedures are in agreement with 2010/63/UE, 86-406, 12-086 and 16-040. For additional details, we refer the reader to the original publication(Tasserie et al., 2022). No statistical methods were used to pre-determine sample sizes but our sample sizes are similar to those reported in previous publications (Luppi et al., 2024b; Tasserie et al., 2022; Uhrig et al., 2018).

Anaesthesia protocol

For the Multi-Anaesthesia dataset, monkeys received anesthesia either with ketamine, propofol, or sevoflurane(Barttfeld et al., 2015; Uhrig et al., 2018), with two different levels of anesthesia for propofol and sevoflurane anesthesia (light and deep). The anesthesia levels were defined according to the monkey sedation scale, based on spontaneous movements and the response to external stimuli (presentation, shaking or prodding, toe pinch), and corneal reflex(Uhrig et al., 2016) and EEG. EEG data were simultaneously acquired during MRI scanning (though they are not analysed in the present study). For each scanning session, the

clinical score was determined at the beginning and end of the scanning session, together with continuous visual monitoring of electroencephalography monitoring(Uhrig et al., 2016). Monkeys were intubated and ventilated(Barttfeld et al., 2015; Uhrig et al., 2018). Heart rate, noninvasive blood pressure, oxygen saturation, respiratory rate, end-tidal carbon dioxide, and cutaneous temperature were monitored (Maglife, Schiller, France) and recorded online (Schiller).

During deep ketamine, deep propofol, and deep sevoflurane anesthesia, monkeys stopped responding to all stimuli, reaching a state of general anesthesia. For ketamine anesthesia, ketamine was injected intramuscular (20 mg/kg; Virbac, France) for induction of anesthesia, followed by a continuous intravenous infusion of ketamine (15 to 16 mg · kg–1 · h–1) to maintain anesthesia. Atropine (0.02 mg/kg intramuscularly; Aguettant, France) was injected 10 min before induction, to reduce salivary and bronchial secretions. For propofol anesthesia, monkeys were trained to be injected an intravenous propofol bolus (5 to 7.5 mg/kg; Fresenius Kabi, France), followed by a target-controlled infusion (Alaris PK Syringe pump, CareFusion, USA) of propofol (light propofol sedation, 3.7 to 4.0 µg/ml; deep propofol anesthesia, 5.6 to 7.2 µg/ml) based on the Paedfusor pharmacokinetic model (Absalom and Kenny 2005). During sevoflurane anesthesia, monkeys received first an intramuscular injection of ketamine (20 mg/kg; Virbac) for induction, followed by sevoflurane anesthesia (light sevoflurane, sevoflurane inspiratory/expiratory, 2.2/2.1 volume percent; deep sevoflurane, sevoflurane inspiratory/expiratory, 4.4/4.0 volume percent; Abbott, France). Only 80 minutes after the induction, the scanning sessions started for the sevoflurane acquisitions to get a washout of the initial ketamine injection (Schroeder et al. 2016). To avoid artefacts related to potential movements throughout magnetic resonance imaging acquisition, a muscle-blocking agent was coadministered (cisatracurium, 0.15 mg/kg bolus intravenously, followed by continuous intravenous infusion at a rate of 0.18 mg · kg–1 · h–1; GlaxoSmithKline, France) during the ketamine and light propofol sessions.

For the DBS dataset (Tasserie et al., 2022), anaesthesia was induced with an intramuscular injection of ketamine (10 mg/kg; Virbac, France) and dexmedetomidine (20 µg/kg; Ovion Pharma, USA) and then the same method as reported above for deep propofol sedation was used (Monkey T: TCI, 4.6 to 4.8 µg/ml; monkey N: TCI, 4.0 to 4.2 µg/ml). Awake scanning data were obtained from the remaining three animals, who did not provide anaesthesia data. Since anaesthesia is identified by behavioural loss of responsiveness, no blinding was possible.

Deep Brain Stimulation protocol

Two monkeys (N and T) were implanted with a clinical DBS electrode (Medtronic, Minneapolis, MN, USA, lead model 3389). The DBS lead had four active contacts for electrical stimulation (1.5-mm contact length, 0.5-mm spacing, and 1.27-mm diameter). We performed stereotactic surgery, targeting the right centro-median thalamus using a neuronavigation system (BrainSight, Rogue, Canada), guided by the rhesus macaque atlases (Paxinos et al., 2008; Saleem and Logothetis, 2012) and a preoperative and intraoperative anatomical MRI [MPRAGE (magnetization prepared - rapid gradient echo), T1-weighted, repetition time (TR) = 2200 ms, inversion time (TI) = 900 ms, 0.80-mm isotropic voxel size, and sagittal orientation]. The electrode was stabilized with the Stimloc lead anchoring device (Medtronic, Minneapolis,

MN, USA). The extracranial part of the DBS lead was hosted using a homemade three-dimensional (3D) printed MRI-compatible chamber. We waited at least 20 days after implantation before starting the DBS-fMRI experiments. Two methods were used to ensure for the anatomical localization of the DBS lead and the DBS contacts. First, a reconstruction method based on in vivo brain imaging. Second, a histology study in one of the implanted monkeys (for more details, see(Tasserie et al., 2022)).

For stimulation, the DBS electrode was plugged to an external stimulator (DS8000, World Precision Instrument, USA), and all the parameters were tuned to a fixed value of frequency (f = 130.208 Hz, T = 7.68 ms), waveform (monopolar signal), and length of width pulse (monkey N, w = 320 μs; monkey T, w = 140 μs). The absolute voltage amplitude was set to 3V ("low" DBS) or 5V ("high" DBS). The resting-state fMRI experiments were acquired either in the awake state or under propofol anaesthesia without ("off" condition) or with a low or high DBS during the entire run on either the CT or VT thalamic nuclei. The DBS started a few seconds before the beginning of the fMRI sequence and stopped just after the end of the MRI sequence(Tasserie et al., 2022).

As reported by Tasserie et al (Tasserie et al., 2022), "DBS significantly affected the general physiology parameters of the anaesthetized monkeys such as the mean heart rate (P = 3.38×10^{-26}) and mean blood pressure (P = 5.78×10^{-17}). For example, for monkey T, high CT-DBS significantly increased mean heart rate (P = 1.23×10^{-23}, compared to anaesthesia; P = 6.76×10^{-19}, compared to low CT-DBS condition) and mean blood pressure (P = 8.66×10^{-14}, compared to anaesthesia condition; P = 6.67×10^{-11}, compared to low CT-DBS condition)".

Behavioural assessment of arousal

We used a preclinical behavioural scale adapted from Uhrig et al (Uhrig et al., 2016) to assess the arousal levels of the monkeys. This scale, based on the Human Observers Assessment of Alertness and Sedation Scale (Chernik et al., 1990) and previously utilised in non-human primate (NHP) research (Vincent et al., 2007), was used consistently across all experimental conditions, in both datasets (Table S1). The behavioural score ranged from 0 to 11, where 11 represented the maximum note achievable and 0 the lowest. The arousal testing occurred outside the MRI environment and was conducted at the beginning and end of each scanning session, for each condition, once the animals were no longer under paralysis.

In all cases, and for both datasets, we observed no differences in arousal scores between different animals in the same condition. For both datasets, the behavioural score during wakefulness was the maximum of 11/11 for all the animals (Monkey A, Monkey K, and Monkey J from the Multi-Anaesthesia dataset, and Monkeys B, J and Y from the DBS dataset): exploration of the surrounding world = 2; spontaneous movements = 2; shaking/prodding = 2; toe pinch = 2; eyes opening = 2; corneal reflex = 1.

For results pertaining to the different anaesthesia conditions of the Multi-Anaesthesia dataset, see Table S25 of (Luppi et al., 2024b). As a summary, deep anaesthesia with ketamine, propofol, or sevoflurane induced an arousal score of 0, consistently in all animals. In contrast, light anaesthesia induced an arousal score of 3 for sevoflurane, and 4 for propofol.

In the anesthesia without DBS ("off") condition, monkeys N and T displayed the minimum behavioral score of 0 over 11, same as the deep anaesthesia from the Multi-Anaesthesia dataset: exploration of the surrounding world = 0; spontaneous movements = 0; shaking/prodding = 0; toe pinch = 0; eyes opening = 0; corneal reflex = 0.

For anesthetized macaques under CT DBS at low amplitude (3V), we measured a clinical score of 3 over 11 (exploration of the surrounding world = 0; spontaneous movements = 0; shaking/prodding = 0; toe pinch = 1, eyes opening = 1; corneal reflex = 1).

When the CT electrical stimulation amplitude was increased to 5V (high-amplitude CT DBS), animals reached a total score of 9 over 11 (exploration of the surrounding world = 1; spontaneous movements = 1; shaking/prodding = 2; toe pinch = 2; eyes opening = 2; corneal reflex = 1).

For VT DBS, both low (3V) and high (5V) amplitude stimulation led to a clinical score of 0, identical to what is observed in the absence of any stimulation.

MRI data acquisition

To minimise head motion, monkeys were head-fixed using an implanted magnetic resonance compatible head post. . For the awake scanning sessions, monkeys trained to sit in the sphinx position in a primate chair(Uhrig et al., 2014) inside the dark magnetic resonance imaging scanner and fixate without any task and the eye position was monitored at 120 Hz (Iscan Inc., USA). The eye-tracking was performed to make sure that the monkeys were awake during the whole scanning session and not sleeping. The eye movements were not regressed out from rfMRI data. For the anesthesia sessions, animals were positioned in a sphinx position, mechanically ventilated, and their physiologic parameters were monitored. No eye-tracking was performed in anesthetic conditions.

For the Multi-Anesthesia dataset, before each scanning session, a contrast agent, monocrystalline iron oxide (MION) nanoparticle (Feraheme, AMAG Pharmaceuticals, USA; 10 mg/kg, intravenous), was injected into the monkey's saphenous vein(Vanduffel et al., 2001). Monkeys were scanned at rest on a 3-Tesla horizontal scanner (Siemens Tim Trio, Germany) with a single transmit-receive surface coil customised to monkeys. Each functional scan consisted of gradient-echo planar whole-brain images (repetition time = 2,400 ms; echo time = 20 ms; 1.5-mm3 voxel size; 500 brain volumes per run).

For the DBS dataset, absence of motion was ensured by paralising the animals with curare. Monkeys were scanned at rest on a 3-Tesla horizontal scanner (Siemens, Prisma Fit, Erlanger Germany) with a customised eight-channel phased- array surface coil (KU Leuven, Belgium). The parameters of the functional MRI sequences were: echo planar imaging (EPI), TR = 1250 ms, echo time (TE) = 14.20 ms, 1.25-mm isotropic voxel size and 500 brain volumes per run. Event-related data pertaining to auditory stimulation were also acquired and are reported in Tasserie et al. (2022), but here we only used the resting-state fMRI data, and will not discuss the event-related data further. Scalp EEG data were also acquired using an MR-compatible system and custom-built caps (EasyCap, 13 channels), an MR amplifier (BrainAmp, Brain Products, Germany), and the Vision Recorder software (Brain Products). These results are

reported in Tasserie et al. (2022), but here we did not consider the EEG data and will not discuss them further.

Functional MRI preprocessing, denoising, and timeseries extraction

For the Multi-Anaesthesia dataset, a total of 157 functional magnetic imaging runs were acquired(Uhrig et al., 2018): Awake, 31 runs (monkey A, 4 runs; monkey J, 18 runs; monkey K, 9 runs), Ketamine, 25 runs (monkey K, 8 runs; monkey Ki, 7 runs; monkey R, 10 runs), Light Propofol, 25 runs (monkey J, 2 runs; monkey K, 11 runs; monkey R, 12 runs), Deep Propofol, 31 runs (monkey J, 9 runs; monkey K, 10 runs; monkey R, 12 runs), Light Sevoflurane, 25 runs (monkey J, 5 runs; monkey Ki, 10 runs; monkey R, 10 runs), Deep Sevoflurane anaesthesia, 20 runs (monkey J, 2 runs; monkey Ki, 8 runs; monkey R, 10 runs). Additional details are available from the original publications(Barttfeld et al., 2015; Signorelli et al., 2020; Uhrig et al., 2018).

Functional images were reoriented, realigned, and rigidly coregistered to the anatomical template of the monkey Montreal Neurologic Institute (Montreal, Canada) space with the use of Python programming language and FMRIB Software Library (FSL) software (United Kingdom, http://www.fmrib.ox.ac.uk/fsl/; accessed February 4, 2018)(Uhrig et al., 2018). From the images, the global signal was regressed out to remove any confounding effect due to physiologic changes (e.g., respiratory or cardiac changes).

For the DBS dataset, a total of 199 Resting State functional MRI runs were acquired: Awake 47 runs (monkey B: 18 runs; monkey J: 13 runs; monkey Y: 16 runs), anaesthesia (DBS-off) 38 runs (monkey N: 16 runs,; monkey T: 22 runs), low amplitude centro-median thalamic DBS 36 runs (monkey N: 18 runs; monkey T: 18 runs), low amplitude ventro-lateral thalamic DBS 20 runs (monkey T), high amplitude centro-median thalamic DBS 38 runs (monkey N: 17 runs; monkey T: 21 runs), and high amplitude ventro-lateral thalamic DBS 20 runs (monkey T: 20 runs)(Tasserie et al., 2022).

Images were preprocessed using Pypreclin (Python preclinical pipeline) (Tasserie et al., 2020). Functional images were corrected for slice timing and B0 inhomogeneities, reoriented, realigned, resampled (1.0 mm isotropic), masked, coregistered to the MNI macaque brain template (Frey et al., 2011) and smoothed (3.0-mm Gaussian kernel). Anatomical images were corrected for B1 inhomogeneities, normalised to the anatomical MNI macaque brain template, and masked.

For both datasets, data were parcellated according to the Regional Map parcellation (Kötter and Wanke, 2005). This parcellation comprises 82 cortical ROIs (41 per hemisphere). Voxel time series were filtered with low-pass (0.05-Hz cutoff) and high-pass (0.0025-Hz cutoff) filters and a zero-phase fast-Fourier notch filter (0.03 Hz) to remove an artifactual pure frequency present in all the data (Barttfeld et al., 2015; Signorelli et al., 2020; Uhrig et al., 2018).

Furthermore, an extra quality control (QC) procedure was performed to ensure the quality of the data after time-series extraction (Signorelli et al., 2020). This quality control procedure is based on trial-by-trial visual inspection by an expert neuroimager (C.M.S.), and it is the same

as was previously implemented in (Luppi et al., 2024b; Signorelli et al., 2020). Its adoption ensures that we employ consistent criteria across our two datasets, by adopting the more stringent of the two. We plotted the time series of each region, as well as the static functional connectivity matrix (FC), the dynamic connectivity (dFC) and a Fourier analysis to detect unconventional spikes of activity. For each dataset, visual inspection was first used to become familiar with the characteristics of the entire dataset: how the amplitude spectrum, timeseries, FC and dynamic FC look. Subsequently, each trial was inspected again with particular focus on two main types of potential artefacts. The first one may correspond to issues with the acquisition and is given by stereotyped sinusoidal oscillatory patterns without variation. The second one may correspond to a head or other movement not corrected properly by our preprocessing procedure. This last artefact can be sometimes recognized by bursts or peaks of activity. Sinusoidal activity generates artificially high functional correlation and peak of frequencies in the Amplitude spectrum plot. Uncorrected movements generate peaks of activity with high functional correlation and sections of high functional correlations in the dynamical FC matrix. If we observed any of these anomalies we rejected the trial, opting to adopt a conservative policy. See Figures S19-S21 from (Luppi et al., 2024b) for examples of artifact-free and rejected trials.

As a result, for the Multi-Anaesthesia data set a total of 119 runs are analysed in subsequent sections (the same as used in Signorelli et al. (Signorelli et al., 2020)): awake state 24 runs, ketamine anaesthesia 22 runs, light propofol anaesthesia 21 runs, deep propofol anaesthesia 23 runs, light sevoflurane anaesthesia 18 runs, deep sevoflurane anaesthesia 11 runs. For the DBS data set, a total of 156 runs are analysed in subsequent sections: awake state 36 runs, Off condition (propofol anaesthesia without stimulation) 28 runs, low-amplitude CT stimulation 31 runs, low-amplitude VT stimulation 18 runs, high-amplitude CT stimulation 25 runs, high-amplitude VT stimulation 18 runs.

Mouse anaesthesia dataset

The mouse fMRI data used here have been previously reported (Gutierrez-Barragan et al., 2021). For clarity and consistency of reporting, where possible we use the same wording as in the original publication (Gutierrez-Barragan et al., 2021).

Animals and ethics

In vivo experiments were conducted in accordance with the Italian law (DL 26/214, EU 63/2010, Ministero della Sanita, Roma) and with the National Institute of Health recommendations for the care and use of laboratory animals (Gutierrez-Barragan et al., 2021). The animal research protocols for this study were reviewed and approved by the Italian Ministry of Health and the animal care committee of Istituto Italiano di Tecnologia (IIT). All surgeries were performed under anesthesia.

Adult (< 6 months old) male C57BL/6J mice were used throughout the study. Mice were group housed in a 12:12 hours light-dark cycle in individually ventilated cages with access to food and water ad libitum and with temperature maintained at 21 ± 1 degrees centigrade and

humidity at 60 ± 10%. All the imaged mice were bred in the same vivarium and scanned with the same MRI scanner and imaging protocol employed for the awake scans (see below). No statistical methods were used to pre-determine sample sizes but our sample sizes are similar to those reported in previous publications (Gutierrez-Barragan et al., 2021).

Experimental groups and datasets

A first group of mice (n = 10 awake C57Bl6/J male mice; awake dataset) underwent head-post surgery, scanner habituation and fMRI image acquisitions as described below. See (Gutierrez-Barragan et al., 2021) for the full surgical, habituation, and scanner protocol. The scans so obtained constitute the awake rsfMRI dataset we used throughout our study. Two additional groups of age matched male C57BL/6J mice were used as reference rsfMRI scans under anesthesia. Since anaesthesia is identified by behavioural loss of responsiveness, no blinding was possible.

The first group of animals (n = 19, halothane dataset) was previously scanned in our laboratory under shallow halothane anaesthesia, 0.75% (Gutierrez-Barragan et al., 2019). The employed anaesthesia regimen is well characterized (Gutierrez-Barragan et al., 2021; Whitesell et al., 2021), it is representative of the network architecture observed with different anaesthesia regimens in rodents (Grandjean et al., 2019) and it exhibits rich spatiotemporal dynamics by preserving spectral properties of fMRI signal fluctuations (Gutierrez-Barragan et al., 2019).

To probe a possible generalizability of our findings to other anesthetic conditions, a second, separate group (n = 14) of mice were imaged under medetomidine-isoflurane anesthesia (0.05 mg/kg bolus and 0.1 mg/kg/h IV infusion, plus 0.5% isoflurane) (Gutierrez-Barragan et al., 2021). While this anesthetic combination is known to shift the spectral components of fMRI signal fluctuations towards higher frequencies (hence departing from the characteristic 1/f power law distribution that characterizes awake and halothane rsfMRI datasets (Grandjean et al., 2019; Gutierrez-Barragan et al., 2019)), it nonetheless represents
the most-widely anesthetic mixture used in the rodent imaging community (Grandjean et al., 2019).

MRI data acquisition

For awake scanning, the mouse was secured using an implanted headpost the custom-made MRI-compatible animal cradle and the body of the mouse was gently restrained (for details of the headpost implantation and habituation protocol, see the original publication (Gutierrez-Barragan et al., 2021)). For scanning under anesthesia, mice were first deeply anesthetized with isoflurane (4% induction), intubated and artificially ventilated (90 BPM). In one group, anesthesia was then switched to halothane (0.75%). In a second group, a bolus of medetomidine (0.05 mg/kg) was given via tail vein cannulation before waiting 5 minutes and starting an infusion of medetomidine (0.1 mg/kg/h) with isoflurane reduced to 0.5%. In both cases, the rsfMRI acquisition started 30 minutes after the switch to light anesthesia (Gutierrez-Barragan et al., 2021).

All scans were acquired at the IIT laboratory in Rovereto (Italy) on a 7.0 Tesla MRI scanner (Bruker Biospin, Ettlingen) with a BGA-9 gradient set, a 72 mm birdcage transmit coil, and a four-channel (awake, halothane) or three-channel (medetomidine-isoflurane) solenoid receive coil. Awake and medetomidine-isoflurane rsfMRI scans were acquired using a single-shot echo planar imaging (EPI) sequence with the following parameters: TR/TE=1000/15 ms, flip angle=60 degrees, matrix=100 x 100, FOV=2.3 x 2.3 cm, 18 coronal slices (voxel-size 230 x 230 x 600 mm), slice thickness=600 mm and 1920 time points, for a total time of 32 minutes. Mice under halothane anesthesia (n = 19) were scanned with a TR/TE=1200/15ms, flip angle=60 degrees, matrix=100 x 100, 24 coronal slices (voxel-size 200 x 200 x 500 mm), for a total of 1600 time points, total acquisition time of 32 minutes as described in Gutierrez-Barragan et al (Gutierrez-Barragan et al., 2021, 2019).

Functional MRI preprocessing, denoising, and timeseries extraction

Preprocessing of fMRI images was carried out as described in previous work (Gutierrez-Barragan et al., 2021). Briefly, the first 2 minutes of the time series were removed to account for thermal gradient equilibration. RsfMRI timeseries were then time despiked (3dDespike, AFNI), motion corrected (MCFLIRT, FSL), skull stripped (FAST, FSL) and spatially registered (ANTs registration suite) to an in-house mouse brain template with a spatial resolution of 0.23 x 0.23 x 0.6mm^3. Denoising involved the regression of 25 nuisance parameters. These were: average cerebral spinal fluid signal plus 24 motion parameters determined from the 3 translation and rotation parameters estimated during motion correction, their temporal derivatives and corresponding squared regressors. No global signal regression was employed. In-scanner head motion was quantified via calculations of frame-wise displacement (FD). Average FD levels in awake conditions were comparable to those obtained in anesthetized animals (halothane) under artificial ventilation ($p = 0.13$, Student t test) (Gutierrez-Barragan et al., 2021). To rule out a contribution of residual head-motion, we further introduced frame-wise fMRI scrubbing (FD > 0.075 mm). The resulting time series were band-pass filtered (0.01-0.1 Hz band) and then spatially smoothed with a Gaussian kernel of 0.5 mm full width at half maximum. Finally, the timeseries were trimmed to ensure that the same number of timepoints were included for all animals, resulting in 1414 volumes per animal. Finally data were parcellated into 162 cortical and subcortical symmetric regions from the Allen Mouse Brain Atlas (CCFv3).

Marmoset FMRI anaesthesia dataset

The marmoset data included here have been published before (Muta et al., 2023). For clarity and consistency of reporting, we use the same wording as in the original publication where possible, and we refer the reader to the original work for details (Muta et al., 2023).

Animals and ethics

This study was approved by the Animal Experiment Committees at the RIKEN Center for Brain Science (CBS) and was conducted per the guidelines for Conducting Animal Experiments of RIKEN CBS. Three male and one female healthy common marmosets (*C. jacchus*) between 3 and 6 years of age were included. All marmosets were examined 12 times to collect

functional MRI data in all conditions. After awake data were firstly collected, and sedate/anesthetic data were done in a random order for sedate/anesthetic condition with an interval of 1 month between each examination in each individual. No statistical methods were used to pre-determine sample sizes but our sample sizes are similar to those reported in previous publications (Muta et al., 2023).

Anesthesia protocol

We used data from awake scans, and from scans under general anaesthesia induced with isoflurane, sevoflurane, or propofol. Essential details are reported below, and we refer to the original publication for additional detail (Muta et al., 2023). Since anaesthesia is identified by behavioural loss of responsiveness, no blinding was possible.

Isoflurane. Three percent isoflurane with 100% O_2 as carrier gas was administered through a facial mask to the marmosets retained with leather gloves. Once sufficient sedation was achieved, an 8 Fr catheter (Atom multi-use tube, Atom Medical Corp., Tokyo, Japan) was inserted into the trachea as an intratracheal tube. The isoflurane concentration was reduced to 2.5%, and 50 μg/kg of atropine and 3.0 mL of physiological saline were administered subcutaneously to prevent intratracheal secretion and dehydration, respectively. After intratracheal intubation, general anesthesia was maintained with 1.8% isoflurane. The marmosets were connected to artificial ventilation for small animals (SN-480-7, Shinano Seisakusho, Tokyo, Japan), and mechanical ventilation was performed under the following conditions: 50% inspiratory oxygen, 8 mL of tidal volume, and 30 breaths per min RR. PR, SpO_2, RR, $EtCO_2$, inspiratory and endtidal isoflurane concentrations, and rectal temperature were measured with a vital sign monitor.

Sevoflurane. All procedures were performed in the same way as isoflurane, except for dosages. The induction of general anesthesia, intratracheal intubation, and maintenance of general anesthesia were performed with 5.0 and 3.0% of sevoflurane (Pfizer Japan Inc., Tokyo, Japan), respectively.

Propofol. Propofol (12 mg/kg) was administered as induction of general anesthesia over 3 min via an indwelling needle. Atropine (50 μg/kg) was administered subcutaneously to prevent intratracheal secretion. Immediately after bolus administration, the predicted plasma concentration of propofol was titrated to 7–9 μg/mL for intratracheal intubation with continuous infusion. The administration dose and protocol were calculated beforehand using a pharmacokinetic parameter reported by (Muta et al., 2023, p. 202) and pharmacokinetic analysis software, NONMEM ver. VII (GloboMax ICON Development Solutions, Ellicott City, MD, USA). Once a sufficient plasma concentration was obtained, the propofol dose was controlled to maintain that concentration. Intratracheal intubation, respiratory management, and vital sign monitoring were performed in the same manner as for isoflurane.

MRI data acquisition

All marmosets underwent a surgical procedure to attach a headpost to the cranial bones to prevent head movement during data collection. During imaging, the marmosets were placed

on a custom-made imaging table (Takashima Seisakusho Co., Ltd, Tokyo, Japan) and immobilized by fixing the head post using a head post fixing tool attached at a custom-made imaging table in all conditions. The marmosets were fitted with earplugs. A hot water circulator was used during imaging to maintain body temperature 36–38 degrees centigrade under all conditions (Muta et al., 2023). In Awake condition, data were collected in the dark and monitored with an infrared camera to prevent the marmosets from falling asleep. If the marmosets were observed closing their eyes during a scan, they were awakened with a loud noise before the next scan was started. They were rewarded with a highly palatable food at the end of each imaging. In anesthetic condition, the marmosets were monitored in the same way to observe spontaneous movement or coughing for intratracheal tube. If spontaneous movement or coughing was observed, additional sedatives/anesthetics were administrated and infusion rate or concentration of inhalational anesthesia was increased.

An ultra-high field MRI system with a static magnetic field strength of 9.4 T (Bruker BioSpin, Ettlingen, Germany), a custom-made 8-channel receiver coil for the marmoset head (Takashima Seisakusho Co., Ltd, Tokyo, Japan), and a 154 mm inner diameter transmitter coil (Bruker BioSpin, Ettlingen, Germany) were used to collect structural and functional data. Structural data and T2-weighted images were imaged using rapid acquisition with relaxation enhancement (RARE) sequence with the following conditions and parameters: time repetition (TR)=4331 ms, time echo (TE) = 15.0 ms, FOV = 42.0 × 28.0 × 36.0 mm, matrix size = 120 × 80 voxels, resolution = 0.35 × 0.35 mm, slice thickness = 0.7 mm, number of slices = 52, scan time = 1 min and 26 s, RARE factor = 4. Functional images were captured using a gradient recalled echo-planar imaging (EPI) sequence with the following conditions and parameters: TR = 2,000 ms, TE = 16.0 mm, FOV = 42.0 × 28.0 × 36.0,mm matrix size = 60 × 40 voxels, resolution = 0.7 × 0.7 mm, slice thickness = 0.7 mm, number of slices = 52, repetition = 155, scan time = 310s. Functional imaging was performed 12 times per animal, per condition (Muta et al., 2023).

Marmoset functional MRI preprocessing and denoising

After the acquired data were converted to Neuro Informatics Technology Initiative format (NIfTI), the voxel size was changed from 0.7 mm isotropic to 3.5 mm isotropic using SPM (Wellcome Trust Center for Neuroimaging, London, UK). Estimation and correction of geometric distortions induced by magnetic susceptibility were performed with the top-up tool of the FMRIB Software Library (FSL) software (FMRIB, Oxford, UK) because all cross-sections were imaged with a single excitation in EPI. Slice timing correction was performed to correct for signal acquisition timing discrepancies in each section. Realignment was applied to compensate for head movements caused by body movements. The deviations in 6 directions were obtained: x (left/right), y (front/back), z (up/down), pitch (rotational direction of nodding and looking up), roll (rotational direction of moving the ear closer to the shoulder), and yaw (rotational direction of looking left/right). For each measurement time point (TR), the deviation from the reference time point, and the first functional brain image, was determined; and the image was moved and rotated by the rigid body model based on this deviation. The method of finding the parameters of the linear transformation was used to minimize the difference between the first functional brain image and the affine transformation of the series of functional brain images to be corrected, by calculating convergence using the method of least squares.

After correcting the spatial scale error between the structural and functional images with co-registration, segmentation was performed to provide information on the tissue to which each voxel belongs in terms of brain tissue classification. The voxels were spatially standardized by normalization, which aligns the voxels to the standard brain image to correct for structural differences between individuals. Smoothing was applied to suppress excessive voxel value fluctuations within individuals and apply normal probability field theory. Functional data were smoothed using spatial convolution with a Gaussian kernel of 2 voxels (7 mm). Then, physiological noise was denoised using ordinary least squares regression with cerebrospinal fluid pulsation, heart rate, and respiratory artifacts as regressors. Temporal band pass filtering was performed by frequency filtering (0.01–0.1 Hz) using the fMRI denoising pipeline of CONN. Finally, preprocessed functional data were parcellated into 70 regions in the cerebral cortex, corresponding to regions of the marmoset MBM atlas (Liu et al., 2018) for which structural connectivity data was also available (see below).

Species-specific connectomes

Human structural connectome

We used diffusion-weighted imaging (DWI) MRI data from the Human Connectome Project. The DWI acquisition protocol is covered in detail elsewhere (Glasser et al., 2013). The diffusion MRI scan was conducted on a Siemens 3T Skyra scanner using a 2D spin-echo single-shot multiband EPI sequence with a multi-band factor of 3 and monopolar gradient pulse. The spatial resolution was 1.25 mm isotropic. TR = 5500 ms, TE = 89.50 ms. The b-values were 1000, 2000, and 3000 s/mm2. The total number of diffusion sampling directions was 90, 90, and 90 for each of the shells in addition to 6 b0 images. We used the version of the data made available in DSI Studio-compatible format at http://brain.labsolver.org/diffusion-mri-templates/hcp-842-hcp-1021 (Yeh et al., 2018).

We adopted previously reported procedures to reconstruct the human connectome from DWI data. The minimally-preprocessed DWI HCP data were corrected for eddy current and susceptibility artifact. After preprocessing, the DTI data were reconstructed using the model-free q-space diffeomorphic reconstruction algorithm (QSDR) implemented in DSI Studio (www.dsi-studio.labsolver.org) (Yeh et al., 2011), following our previous work (Luppi and Stamatakis, 2021). QSDR initially reconstructs DWI data in native space, and subsequently computes values of quantitative anisotropy (QA) in each voxel, based on which DSI Studio performs a nonlinear warp from native space to a template QA volume in Montreal Neurological Institute (MNI) space. Once in MNI standard space, spin density functions are reconstructed, with a mean diffusion distance of 1.25 mm with three fiber orientations per voxel (Yeh et al., 2011). Finally, fiber tracking was carried out by means of DSI Studio's own "FACT" deterministic tractography algorithm, requesting 1,000,000 streamlines according to widely adopted parameters (Luppi and Stamatakis, 2021): angular cutoff = 55◦, step size = 1.0 mm, tract length between 10mm (minimum) and 400mm (maximum), no spin density function smoothing, and QA threshold determined by DWI signal in the cerebro-spinal fluid. Streamlines were automatically rejected if they presented improper termination locations, based on a white matter mask automatically generated by applying a default anisotropy

threshold of 0.6 Otsu's threshold to the anisotropy values of the spin density function (Luppi and Stamatakis, 2021).

For each individual, their structural connectome was reconstructed by drawing an edge between each pair of regions *i* and *j* if there were white matter tracts connecting the corresponding brain regions end-to-end; edge weights were quantified as the number of streamlines connecting each pair of regions. A group-consensus matrix *A* across subjects was then obtained using the approach previously adopted for whole-brain modelling by Wang et al. (Wang et al., 2019), whereby an edge was retained between two regions if it was present in at least 50% of subjects, and its weight was given by the average weight.

Macaque structural connectome

Anatomical (structural) connectivity data were derived from the recent macaque connectome of (Shen et al., 2019), which combines diffusion MRI tractrography with axonal tract-tracing studies from the CoCoMac database (Bakker et al., 2012), representing the most complete representation of the macaque connectome currently available. Structural connectivity data are expressed as a matrix in which the 82 cortical regions of interest are displayed in x-axis and y-axis. Each cell of the matrix represents the strength of the anatomical connection between any pair of cortical areas. For consistency with the human structural connectome, a symmetrised connectome was used, as in our previous work (Luppi et al., 2024b).

Macaque thalamocortical structural connectivity

The DTI-CoCoMac connectome from The Virtual Brain does not include thalamic nuclei. To obtain a macaque connectome with thalamic nuclei, we combined the 82-ROI RM parcellation with thalamic nuclei from the SARM macaque subcortical atlas (Hartig et al., 2021). We then performed diffusion tractography between the regions of this augmented RM atlas, based on diffusion MRI data from N=19 macaque monkeys (*Macaca mulatta*) from multiple institutions, collected on the PRIME-DE platform (Michael Milham et al., 2018). Preprocessed dMRI data in DSI Studio format are available on Zendo (DOI: 10.5281/zenodo.6321168). The dMRI were processed by FSL TOPUP/EDDY and DSI Studio to generate SRC Files. The DTI data were reconstructed using the model-free q-space diffeomorphic reconstruction algorithm (QSDR) implemented in DSI Studio (www.dsi-studio.labsolver.org) (Yeh et al., 2011). QSDR initially reconstructs DWI data in native space, and subsequently computes values of quantitative anisotropy (QA) in each voxel, based on which DSI Studio performs a nonlinear warp from native space to a template QA volume (for macaques, the INDI template). For 4 animals, DSI's reconstruction quality metric (R-squared value between the individual QA and template QA map) was lower than .60; these animals were excluded from further analysis, leaving N=15. All other animals had R^2 of .67 or greater. Once in standard space, spin density functions are reconstructed, with a mean diffusion distance of 1.25 mm with three fiber orientations per voxel (Yeh et al., 2011). Finally, fiber tracking was carried out by means of DSI Studio's own "FACT" deterministic tractography algorithm, requesting 10,000,000 streamlines, according to widely adopted parameters (coinciding with those described above for obtaining the human consensus connectome) (Gu et al., 2015): angular cutoff = 55◦, step

size = 1.0 mm, tract length between 10mm (minimum) and 400mm (maximum), no spin density function smoothing, and QA threshold determined by DWI signal in the cerebro-spinal fluid. Streamlines were automatically rejected if they presented improper termination locations, based on a white matter mask automatically generated by applying a default anisotropy threshold of 0.6 Otsu's threshold to the anisotropy values of the spin density function (Gu et al., 2015).

The thalamus-augmented RM atlas was mapped from NMT space to INDI template space by using the RheMap toolbox (Sirmpilatze and Klink, 2020), and connectivity between the 82 cortical regions of the RM atlas and the CT and VT was then computed as the number of streamlines connecting regions end-to-end. We then aggregated the connectivity across animals: for each cortical region *i* and thalamic region *j*, the value of the thalamocortical connectivity between *i* and *j* was set to the median value of non-zero connections between *i* and *j* across animals, provided that an edge was present in more than just one animal (to avoid false positives). Otherwise, the edge between *i* and *j* was set to zero. Finally, an augmented macaque structural connectome was obtained by combining the thalamocortical connections returned via this procedure, and the cortico-cortical connectome of Shen and colleagues, each rescaled to have edge weights between 0 and 1.

Marmoset structural connectome

The marmoset structural connectome was obtained from the Brain/MINDS Marmoset MRI NA216 in-vivo database (https://doi.org/10.24475/bminds.mri.thj.4624) (Hata et al., 2023). Full details are provided in the original data-release publication (Hata et al., 2023). Briefly: *in vivo* MR imaging was conducted using a 9.4 T BioSpec 94/30 (Bruker Optik GmbH, Ettlingen, Germany) unit and a transmitting and receiving coil with an 86-mm inner diameter. Diffusion weighted spin-echo echo planar imaging was used with TR = 3000 ms, TE = 25.6 ms, b-value = 1000 and 3000 s/mm2 in 30 and 60 diffusion directions, respectively (plus 4 b0 images), number of segments = 6, FA = 90, NA = 3, voxel size = 350 × 350 × 700 micrometers, and scan time = 90 min. Diffusion metrics were created by DTI model, and diffusion fiber structural connectome was created by constrained spherical deconvolution, using the number of streamlines between each pair of 104 brain regions (37 cortical and 15 subcortical per hemisphere) (Hata et al., 2023). Here we used the structural connectivity between 70 pairs of regions (35 per hemisphere) that have a correspondence with the cortical regions of the marmoset MBM atlas (Liu et al., 2018).

Mouse structural connectome

For the mouse structural connectome, we used a parcellated version of the high-resolution mouse connectome of Coletta *et al.* (Coletta et al., 2020). Below, we summarise how Coletta and colleagues obtained the high-resolution mouse structural connectome.

The present mouse structural connectome is based on "high-resolution models of the mouse brain connectome (100 µm^3) previously released by Knox and colleagues (Knox et al., 2018). The Knox connectome is based on 428 viral microinjection experiments in C57BL/6J male

mice obtained from the Allen Mouse Brain Connectivity Atlas (http://connectivity.brain-map.org/). The connectome data were derived from imaging enhanced green fluorescent protein (eGFP)–labeled axonal projections that were then registered to the Allen Mouse Brain Atlas and aggregated according to a voxel-wise interpolation model (Knox et al., 2018)".

Before constructing the SC matrix, Coletta et al. ensured symmetry along the right-left axis for all the major macrostructures of the mouse brain. To this purpose, they "flipped each macrostructure (isocortex, hippocampal formation, subcortical plate, pallidum, striatum, pons, medulla, midbrain, thalamus, hypothalamus, cerebellum, and olfactory bulb) along the sagittal midline (once for the right hemisphere and once for the left hemisphere) and took the intersection with the respective nonflipped macrostructure. This procedure resulted in the removal of a set of nonsymmetric voxel (total fraction, 8.6%), the vast majority of which reside in fringe white/gray matter or cerebrospinal fluid/gray matter interfaces. The removal of these nonsymmetric voxels did not substantially affect the network structure of the resampled connectome, as assessed with a spatial correlation analysis between the symmetrized and nonsymmetrized right ipsilateral (i.e., squared) connectome". Coletta et al. then filtered out fiber tracts and ventricular spaces, and estimated SC using a resampled version of the recently published voxel scale model of the mouse structural connectome (Knox et al., 2018), to make the original matrix computationally tractable. Resampling of the Knox et al. connectome was carried out by aggregating neighboring voxels according to a Voronoi diagram based on Euclidean distance between neighboring voxels, preserving the intrinsic architectural foundation of the connectome while minimizing spatial blurring and boundary effects between ontogenically distinct neuroanatomical divisions of the mouse brain, or white/gray matter, and parenchymal/ventricular interfaces (see Coletta et al. for details of the Voronoi aggregation scheme (Knox et al., 2018)). By averaging the connectivity profile of neighboring voxels based on their relative spatial arrangement, this strategy has also the advantage of mitigating limitations related to the enforced smoothness of source space used by the original kernel interpolation used by (Knox et al., 2018).

A whole-brain connectome was then built under the assumption of brain symmetry (Coletta et al., 2020). Forty-four dangling nodes (i.e., nodes with no outgoing connectivity) were next removed from the resulting matrix, resulting in a final weighted and directed 15,314 × 15,314 matrix composed of 0.027-mm3 aggregate Voronoi voxels. The obtained Voronoi diagram made it possible to map the results back into the original 100-μm three-dimensional coordinate system of the Allen Institute mouse brain connectome [CCFv3]. For each pair of the 162 Allen Atlas regions that we employed, their structural connectivity was obtained by averaging the connectivity of the respective constituent voxels. For consistency with the human structural connectome, we used a symmetrised connectome.

Gene expression datasets

Human gene expression from microarray probes

Human gene expression profiles were obtained using microarray data from the Allen Human Brain Atlas (AHBA) (Hawrylycz et al., 2012). We followed the same preprocessing as recently described (Zarkali et al., 2022). Briefly, the Allen Human Brain Atlas (AHBA) is a publicly

available transcriptional atlas containing gene expression data measured with DNA microarrays and sampled from hundreds of histologically validated neuroanatomical structures across six (five male and one female) normal postmortem human brains. We extracted and mapped gene expression data to the 100 cortical ROIs of the Schaefer parcellation using the *abagen* toolbox (Markello et al., 2021). Data was pooled between homologous cortical regions to ensure adequate coverage of both left (data from six donors) and right hemisphere (data from two donors). Distances between samples were evaluated on the cortical surface with a 2 mm distance threshold. Only probes where expression measures were above a background threshold in more than 50% of samples were selected. A representative probe for a gene was selected based on highest intensity. Gene expression data were normalised across the cortex using scaled, outlier-robust sigmoid normalisation. 15,633 genes survived these preprocessing and quality assurance steps. The same normalisation was applied to subcortical data. To characterise the core-matrix architecture of the thalamus, the normalised regional expression of *PVALB* mRNA was subtracted from the normalised regional expression of *CALB1* mRNA, such that *CALB1* -dominated nuclei are more matrix-like, and *PVALB*-dominated nuclei are more core-like, as per the procedure of (Huang et al., 2024; Müller et al., 2020).

Macaque gene expression from high-resolution spatial transcriptomics

We used cortex-wide macaque gene expression data recently made available by (Chen et al., 2023), who combined single-nucleus RNA sequencing ("snRNA-seq") with high-resolution, large-field-of view spatial transcriptomics from spatiotemporal enhanced resolution omics sequencing ("stereo-seq") (Chen et al., 2023). Specifically, the authors made available (https://macaque.digital-brain.cn/ spatial-omics) post-mortem gene expression data covering 143 regions of the left cortical hemisphere of one 6yo male cynomolgus macaque (*Macaca fascicularis*). We refer the reader to (Chen et al., 2023) for details. Briefly, Chen and colleagues obtained 119 coronal sections at 500-μm spacing, covering the entire cortex of the left hemisphere, which were used for stereo-seq transcriptomics (Chen et al., 2023). Adjacent 50-μm thick sections were also acquired for regional microdissection and snRNAseq analysis, as well as 10-μm sections adjacent to each stereo-seq section, which were used for the anatomical parcellation of brain regions via immunostaining (Chen et al., 2023). Stereo-seq is a DNA nanoball (DNB) barcoded solid-phase RNA capture method that involves reverse transcription of RNAs released from frozen tissue sections fixated onto the stereo-seq chip, and subsequent PCR amplification. The resulting "amplified barcoded complementary DNA (cDNA) is used as template for library preparation, and sequenced" to obtain high-resolution spatially resolved transcriptomics (Chen et al., 2023). Gene expression data were made available by (Chen et al., 2023) for 143 cortical regions of the left hemisphere, including "prefrontal, frontal, cingulate, somatosensory, insular, auditory, temporal, parietal, occipital and piriform areas". As reported in (Chen et al., 2023), "for each coronal section, the cortical region and layer parcellation were manually delineated on Stereo-seq data background, based on cytoarchitectual pattern (e.g. cell density, cell size) revealed by total mRNA expression, nucleic acid staining, and NeuN staing of adjacent sections." Since the original parcellation used by (Chen et al., 2023) is different from the Regional Mapping macaque cortical atlas (Kötter and Wanke, 2005) that was used for our macaque functional MRI data, here we used the version of the macaque gene expression data that was mirrored between hemispheres and mapped onto the "Regional Mapping" macaque cortical atlas, as made available by (Luppi

et al., 2024a). For consistency across datasets, the gene expression data were normalised across regions using scaled, outlier-robust sigmoid normalisation.

Mouse gene expression from in situ hybridization

Mouse gene expression profiles were obtained using in situ hybridization data from the Allen Mouse Brain Atlas (Lein et al., 2007). We followed the same preprocessing as recently described (Yee et al., 2018). Briefly, the Allen Mouse Brain Atlas consists of data acquired from a pipeline that includes semi-automated riboprobe generation, tissue preparation and sectioning, in-situ hybridization (ISH), imaging, and data post-processing. These data were acquired both sagittally and (for a smaller set of genes) coronally, and were further processed, aligned by the Allen Institute to their Common Coordinate Framework version 3 (CCFv3) reference atlas (Wang et al., 2020) and summarized voxelwise through a measure termed gene expression energy (defined as the sum of expressing pixel intensity divided by the sum of all pixels in a division), resulting in 3D gene expression images at a 200 μm isotropic resolution. This gene expression energy increases in regions of high expression, and is bounded by zero in regions of no expression. For our study, we used gene expression energy data from the coronal dataset (4345 gene expression images corresponding to 4082 unique genes), because of its whole-brain coverage and data quality. Fernandes et al (Fernandes et al., 2017) provide tools to work with this gene expression data; these tools are available online (github.com/DJFernandes/ABIgeneRMINC). Voxelwise gene expression data were further summarized as normalized mean expression within regions of interest as defined by the CCFv3 reference atlas. For each ROI and each gene, voxelwise expression energy data were averaged over voxels containing valid expression signal. Finally, the gene expression data were normalised across regions using scaled, outlier-robust sigmoid normalisation. To characterise the core-matrix architecture of the thalamus, the normalised regional expression of *Pvalb* mRNA was subtracted from the normalised regional expression of *Calb1* mRNA, such that *Calb1*-dominated nuclei are more matrix-like, and *Pvalb*-dominated nuclei are more core-like, mirroring the procedure of (Huang et al., 2024; Müller et al., 2020) to identify 'matrix' and 'core' thalamic nuclei in humans.

Neurobiological validation datasets

We obtained the density of parvalbumin-expressing neurons from Burt et al. (Burt et al., 2018), who compiled, from multiple immunohistochemical studies, the relative densities of inhibitory interneurons which are immunoreactive to parvalbumin. We used the version of these data mapped onto the 82 regions of the RM macaque cortical atlas, as made available by (Luppi et al., 2024a). We also obtained mouse regional count of PV+ neurons from Kim, Yang et al. (Kim et al., 2017), who "developed a cell counting and distribution mapping platform, based on automated imaging by serial two-photon tomography (STPT) and data analysis by machine learning algorithms" and released tables with cell counts and cell densities for the Allen Mouse Brain Atlas regions (Wang et al., 2020), available at

http://mouse.brainarchitecture.org/cellcounts/ost/. Immunohistochemically-derived measurements of the relative prevalence of calbindin-positive and parvalbumin-positive neurons in different thalamic nuclei were obtained from Bjerke et al (Bjerke et al., 2021). Nuclei were then ranked from most calbindin-dominated to most parvalbumin-dominated, mirroring the procedure of (Huang et al., 2024; Müller et al., 2020) to identify 'matrix' and 'core' thalamic nuclei in humans. Note that Bjerke and colleagues did not provide data for the reticular nucleus of the thalamus, because this region was oversaturated with staining(Bjerke et al., 2021).

Whole-brain modelling with FastDMF

Briefly, macroscale whole-brain computational models represent regional activity in terms of two key ingredients: (i) a biophysical model of each region's local dynamics; and (ii) inter-regional anatomical connectivity. Thus, such *in silico* models provide a well-suited tool to investigate how the structural connectivity of the brain shapes the corresponding macroscale neural dynamics (Cabral et al., 2017; Cofré et al., 2020; Deco and Kringelbach, 2014; Demirtaş et al., 2019; Kringelbach and Deco, 2020; Luppi et al., 2023a; Shine et al., 2021; Wang et al., 2019). In particular, the Dynamic Mean Field (DMF) model employed here simulates each region (defined via a species-specific brain parcellation scheme) as a macroscopic neural field comprising mutually coupled excitatory and inhibitory populations, providing a neurobiologically plausible account of regional neuronal firing rate. Specifically, the model simulates local biophysical dynamics of excitatory (NMDA) and inhibitory (GABA) neuronal populations, interacting over long-range neuroanatomical connections. Here, we used species-specific structural connectomes, obtained as described above.

The DMF model has one free parameter, known as "global coupling" and denoted by *G*, which controls the overall strength of signal transmission between brain regions (conductivity of the white matter fibers is assumed to be constant across the brain). The following differential equations therefore govern the model's behaviour:

$$I_n^{(E)} = W_E I_0 + w_+ J_{NMDA} S_n^{(E)} + G J_{NMDA} \sum_p^N C_{np} S_p^{(E)} - J_n^{FIC} S_n^{(I)} \tag{17}$$

$$I_n^{(I)} = W_I I_0 + J_{NMDA} S_n^{(E)} - S_n^{(I)} \tag{18}$$

$$r_n^{(E)} = F\left(I_n^{(E)}\right) = \frac{g_E \left(I_n^{(E)} - I_{thr}^{(E)}\right)}{1 - exp\left(-d_E g_E \left(I_n^{(E)} - I_{thr}^{(E)}\right)\right)} \tag{19}$$

$$r_n^{(I)} = F\left(I_n^{(I)}\right) = \frac{g_n^{NM} g_I \left(I_n^{(I)} - I_{thr}^{(I)}\right)}{1 - exp\left(-d_I g_n^{NM} g_I \left(I_n^{(I)} - I_{thr}^{(I)}\right)\right)}$$

(20)

$$\frac{dS_n^{(E)}(t)}{dt} = \frac{S_n^{(E)}}{\tau_{NMDA}} + \left(1 + S_n^{(E)}\right) \gamma r_n^{(E)} + \sigma v_n(t)$$

(21)

$$\frac{dS_n^{(I)}(t)}{dt} = \frac{S_n^{(I)}}{\tau_{GABA_A}} + r_n^{(I)} + \sigma v_n(t)$$

(22)

Following previous work, for each excitatory *(E)* and inhibitory *(I)* neural mass, the quantities $I_n^{(E,I)}$, $r_n^{(E,I)}$, and $S_n^{(E,I)}$ represent its total input current (nA), firing rate (Hz) and synaptic gating variable, respectively. The function F(·) is the transfer function (or *F–I curve*), representing the non-linear relationship between the input current and the output firing rate of a neural population. Finally, J_n^{FIC} is the local feedback inhibitory control of region *n*, which is optimized to keep its average firing rate at approximately 3Hz, and v_n is uncorrelated Gaussian noise injected to region *n* (Deco et al., 2018; Herzog et al., 2022; Luppi et al., 2023c, 2022). The model's fixed parameters are reported in Table S4. Therefore, the global coupling *G* remains the sole free parameter in the model.

To find the most suitable value of *G* for the simulation of each species, we adopted the approach previously described (Deco et al., 2018; Herzog et al., 2022; Luppi et al., 2023c, 2022) which aims to obtain the best match between empirical and simulated functional connectivity dynamics (FCD). The FCD is computed as follows. First, we obtain Pearson correlation matrices between regional fMRI signal timeseries, computed within a sliding window of 30 TRs with increments of 3 TRs (Deco et al., 2018; Herzog et al., 2022; Luppi et al., 2023c, 2022). Subsequently, the resulting matrices of functional connectivity at times t_i and t_j are themselves correlated, for each pair of timepoints t_i and t_j, thereby obtaining an FCD matrix of time-versus-time correlations. Thus, each entry in the FCD matrix represents the similarity between functional connectivity patterns at different points in time. This procedure is performed for the empirical data from the awake condition of each species, and also for model-generated data simulated at different values of the *G* parameter. A Balloon-Windkessel hemodynamic model (Friston et al., 2003) is used to turn simulated regional neuronal activity into simulated regional fMRI signal, and the simulated regional BOLD signal is bandpass

filtered in the same range as each empirical dataset. We then used a Bayesian optimisation algorithm (Herzog et al., 2022) to identify the value of the *G* parameter that minimises the Kolmogorov-Smirnov distance between the histograms of empirical (group-wise) and simulated FCD values (obtained from the upper triangular FCD matrix), for each condition, following previous work (Deco et al., 2018). Finally, we set the model's *G* parameter to the value that was observed to minimise the mean KS distance - corresponding to the model that is best capable of simulating the temporal dynamics of resting-state brain functional connectivity. Use of the FCD is well established as fitting target for the DMF model (Deco et al., 2018; Luppi et al., 2023c, 2022). After having found the best-fitting value of *G* for each condition (see Table S5), 41 further simulations were obtained from the corresponding DMF model with the optimal *G* parameter, and were used for computing integrated information in the same way as the empirical data. The code used to run all the simulations in this study was written in optimised C++ using the high-performance library Eigen. The C++ core of the code, together with Python and Octave/Matlab interfaces is publicly available as "FastDMF" [132] and maintained at http://www.gitlab.com/concog/fastdmf.

Statistical reporting

For the human sevoflurane dataset, statistical significance was assessed using a non-parametric paired-samples t-test. Use of a non-parametric test ensures robustness to violations of the normality assumption, which was not formally tested. Effect sizes are provided as Hedge's measure of standardised difference *g,* which is analogous to Cohen's *d,* but recommended for smaller sample sizes such as the ones available in the present study. For the mouse dataset, statistical significance was assessed using a non-parametric independent-samples t-test. Use of a non-parametric test ensures robustness to violations of the normality assumption, which was not formally tested. Effect sizes are provided as Hedge's *g*. For the marmoset and both macaque datasets (Multi-Anaesthesia and DBS), pairwise contrasts (two-sided) were carried out using linear mixed effects modelling (implemented using MATLAB's *fitlme* function), with condition as the fixed effect and animal identity as the random effect (Luppi et al., 2024b). Estimated effect is also provided. This approach enabled us to take into account the fact that the same animal could provide more than one data-point to each condition, as well as contributing data-points for more than one condition (Luppi et al., 2024b). For the marmoset dataset and the macaque Multi-Anaesthesia dataset, each anaesthesia condition was compared against wakefulness, to test the hypothesis that our neural markers are affected by anaesthesia. For the macaque DBS dataset, we compared the No-DBS anaesthesia condition, against all other conditions: wakefulness, and all stimulation types. This allowed us to replicate the effects of anaesthesia on our neural markers of interest (since one of the included comparisons is Awake against no-stimulation anaesthesia), and to test the hypothesis that CT stimulation should counter the effects of anaesthesia on our neural markers (Luppi et al., 2024b). In all datasets, correction for multiple comparisons against the same condition was carried out using the False Discovery Rate procedure (Benjamini and Hochberg, 1995). We also repeated the transition energy analyses using mean framewise displacement (FD) as a covariate of no interest, to account for potential confounding effects of motion.

The network-based statistic (NBS) (Zalesky et al., 2010) was used to investigate the statistical significance of edge-wise changes in integrated information. NBS is a nonparametric statistical method for network data that controls the family-wise error due to multiple comparisons (Zalesky et al., 2010). Connected components of the graph are identified from edges that survive an a-priori statistical threshold (F-contrast; here we set the threshold to an F-value of 9, two-sided, with an alpha level of 0.05). Subsequently, the statistical significance of the identified connected components is assessed by comparing the empirical connected component size, against a null distribution of the size of connected components obtained from non-parametric permutation testing. Since this method rejects the null hypothesis on a component-by-component level, it achieves superior power compared to mass-univariate approaches (Zalesky et al., 2010).

Surrogate data from null models

To control for the spatial autocorrelation inherent in neuroimaging data (Alexander-Bloch et al., 2018; Markello and Misic, 2021; Váša and Mišić, 2022), we assessed the statistical significance of correlations with gene expression maps non-parametrically, by comparing each empirical correlation against a distribution of 10,000 correlations with surrogate null maps having the same spatial autocorrelation. Surrogate null maps were generated using Moran spectral randomisation based on the inverse Euclidean distances between parcel centroids, as implemented in the *BrainSpace* toolbox (https://brainspace.readthedocs.io/en/latest/) (Vos de Wael et al., 2020). Moran spectral randomisation quantifies the spatial autocorrelation in the data in terms of Moran's *I* coefficient (Dray, 2011; Vos de Wael et al., 2020; Wagner and Dray, 2015), by computing spatial eigenvectors known as Moran eigenvector maps. The Moran eigenvectors are then used to generate null maps data by imposing the spatial structure of the empirical data on randomised surrogate data (Vos de Wael et al., 2020). The same approach was also used to generate surrogate gene expression maps for the network control energy calculation, and for the biophysical model with regionally heterogeneous inhibition. By breaking the correspondence between heterogeneity and anatomy, use of these surrogate maps enables us to assess their respective contributions.

To obtain surrogate null time-series for computing the bias in our information-theoretic quantification (Koçillari et al., 2024), the empirical time-series for each subject in each condition were randomly reshuffled in time, subject to the constraint of preserving the instantaneous synchrony between regions (thereby preserving the correlation between pairs of time-series). This procedure destroys the past-future relationships on which integrated information is predicated, such that any remaining value can be used to quantify the estimation bias in our measures.

Dominance analysis

Unlike other methods of assessing predictor importance, such as methods based on regression coefficients or univariate correlations, dominance analysis accounts for predictor-predictor interactions and its interpretation is straightforward. Specifically, dominance analysis seeks to determine the relative contribution ("dominance") of each independent variable to the overall fit (adjusted R^2) of a multiple linear regression model (Azen and Budescu, 2003; Hansen et al., 2022). This is done by fitting the same regression model on every combination

of predictors ($2^p - 1$ submodels for a model with p being the predictors). Total dominance is defined as the average of the relative increase in R^2 when adding a single predictor of interest to a submodel, across all $2^p - 1$ submodels. The sum of the dominance of all input variables is equal to the total adjusted R^2 of the complete model, from which a percentage of relative importance is obtained by partitioning the total variance accounted for by each predictor.

As target variable, we use the arousal score corresponding to each condition, on a scale from 0 to 11 (Uhrig et al., 2016). As regressors, we use the data pertaining to our measures of interest: integrated information and the other information-theoretic measures. We repeat this analysis with combined data across the macaque Multi-Anaesthesia and DBS datasets. To ensure that data could be combined across the DBS and Multi-Anaesthesia datasets, values of each marker were normalised between 0 and 1 separately within each dataset (by subtracting the minimum value observed within the dataset, and then dividing by the maximum) before aggregating the two datasets.

To assess statistical significance of the dominance analysis multiple regression, we performed a one-sided non-parametric permutation test: we built a null distribution of adjusted R^2 values by repeating the multiple regression 1,000 times, but with permuted assignment of the arousal scores, and evaluating how often the null adjusted R^2 was greater than the empirical adjusted R^2 (Luppi et al., 2024b).

Supplementary Discussion

Integrated Information beyond IIT 2.0

Note that the formula for the original Φ_{2008} above stems from what is known as IIT 2.0, but TDMI is by no means the only way of quantifying the dynamical structure of a system. Alternatives include variants based on mismatched decoding (Φ^*) (Oizumi et al., 2016a), information geometry (Φ^G) (Oizumi et al., 2016b; Toker and Sommer, 2019), stochastic interaction (Ay, 2015; Barrett and Seth, 2011), Causal Density (Seth et al., 2011), and others still (Griffith, 2014; Langer and Ay, 2020; Tononi et al., 1994), including some that are based on causal perturbations or a stronger, non-Shannon formulations of "intrinsic information" (Barbosa et al., 2021a, 2020) (see (Mediano et al., 2025, 2022; Tegmark, 2016) for comparative reviews). Indeed, Integrated Information Theory has found alternative ways to overcome some of the perceived shortcoming of IIT 2.0's Φ_{2008}: subsequent developments in IIT 3.0 and 4.0 used alternative metrics with a more explicit focus on causal interpretations (Albantakis et al., 2022; Barbosa et al., 2021b; Marshall et al., 2023; Oizumi et al., 2014) and explicit formalisation of "intrinsic" information (Barbosa et al., 2021a, 2020) which differs from Shannon information. Here, we do not consider the alternative measures of integrated information proposed in IIT 3.0 and 4.0 because they are currently computationally intractable for systems bigger than a small set of logic gates (Barrett and Mediano, 2019). Indeed, our intention here is *not* to test Integrated Information Theory (in any of its versions – 2.0, 3.0 or 4.0). Rather, in the spirit of "weak Integrated Information Theory" and its focus on practical measures for real brain data (Mediano et al., 2022), we build on the core intuition of quantifying the coexistence of integration and differentiation of information – an intuition dating back at least to (Seth et al., 2011; Tononi et al., 1994; Tononi and Edelman, 1998).

The thalamus and other putative controllers of consciousness

The central thalamus consists of several intra- and para-laminar thalamic nuclei that act as intermediaries between arousal systems (receiving afferent projections from the brainstem ascending Reticular Activating System (Moruzzi and Magoun, 1949; Steriade and Glenn, 1982)) and extensive cortical networks (Schiff, 2008), as well as entertaining bi-directional projections to and from the basal ganglia (Shine, 2020; Shine et al., 2023).

Earlier studies had reported effects of selective thalamic stimulation on responsiveness and arousal in animals (Alkire et al., 2007; Bastos et al., 2021; McCafferty et al., 2023; Redinbaugh et al., 2022, 2020; Tasserie et al., 2022; Xu et al., 2020) and human patients (Laureys et al., 2000; ND Schiff, 2007; Staunton, 2008), including evidence that electrical stimulation of specific central-lateral thalamic nuclei (but not control sites) can restore arousal and putative electrophysiological markers of consciousness during anaesthetic-induced loss of responsiveness in macaques (Bastos et al., 2021; Redinbaugh et al., 2020; Tasserie et al., 2022), counteracting the loss of high-frequency activity and communication between the thalamus and deep cortical layers, which is induced by anaesthesia. Other groups applied

optogenetic in rodent models of consciousness loss and could demonstrate a direct link between the modulation of CT neurons and the transition in the state of consciousness(Gent et al., 2018; Liu et al., n.d.). In particular, our findings are consistent with previous studies(Afrasiabi et al., 2021; Bastos et al., 2021; Redinbaugh et al., 2020) since modelling the volume of activated tissue(Tasserie et al., 2022) indicated that our electrical stimulation encompassed the same targets as these groups, including intra-laminar central thalamic nuclei (Schiff, 2008).

However, this evidence should not be interpreted as indicating that the thalamus is the sole arbiter of consciousness. Other subcortical structures such as the basal ganglia are known to play an important role in anaesthetic-induced and pathological loss of responsiveness (Brown et al., 2010; Guang et al., 2021; Hemmings et al., 2019; Mhuircheartaigh et al., 2010; Moruzzi and Magoun, 1949). There is also clear evidence for the role of the brainstem and ascending arousal system in controlling loss of consciousness (Fischer et al., 2016; Gao et al., 2019; Mashour, 2024; Solt et al., 2014; Spindler et al., 2021; Taylor et al., 2016; Vincent et al., 2024). Indeed, the dopaminergic ventral tegmental area projects widely to both the central thalamus and the cortex (Edlow et al., 2024; Li et al., 2021) and can be targeted by electrical stimulation or optogenetics to reverse anaesthesia in rodents (Taylor et al., 2016; Vincent et al., 2024). The evidence of anatomical connectivity between central thalamus and prefrontal cortex (Morais et al., 2023) means that the present evidence are also consistent with rodent evidence that PFC stimulation via carbachol administration awakens rats from sevoflurane anaesthesia (Pal et al., 2018), whereas tetrodotoxin-mediated inactivation of rat medial PFC had the opposite effect, delaying recovery(Huels et al., 2021). Likewise, PFC inactivation also diminishes the ability of basal forebrain stimulation to promote arousal from sevoflurane anaesthesia(Dean et al., 2022). Although such studies are yet to be translated to humans or non-human primates, they suggest the possibility that PFC stimulation may also represent an avenue of achieving similar effects as CT stimulation, both thanks to direct connections between the two, as well as via striatum-mediated disinhibition of the thalamus via descending arousal pathways (Luppi et al., 2024b; Mashour, 2024; Mashour et al., 2022).

Supplementary Figures

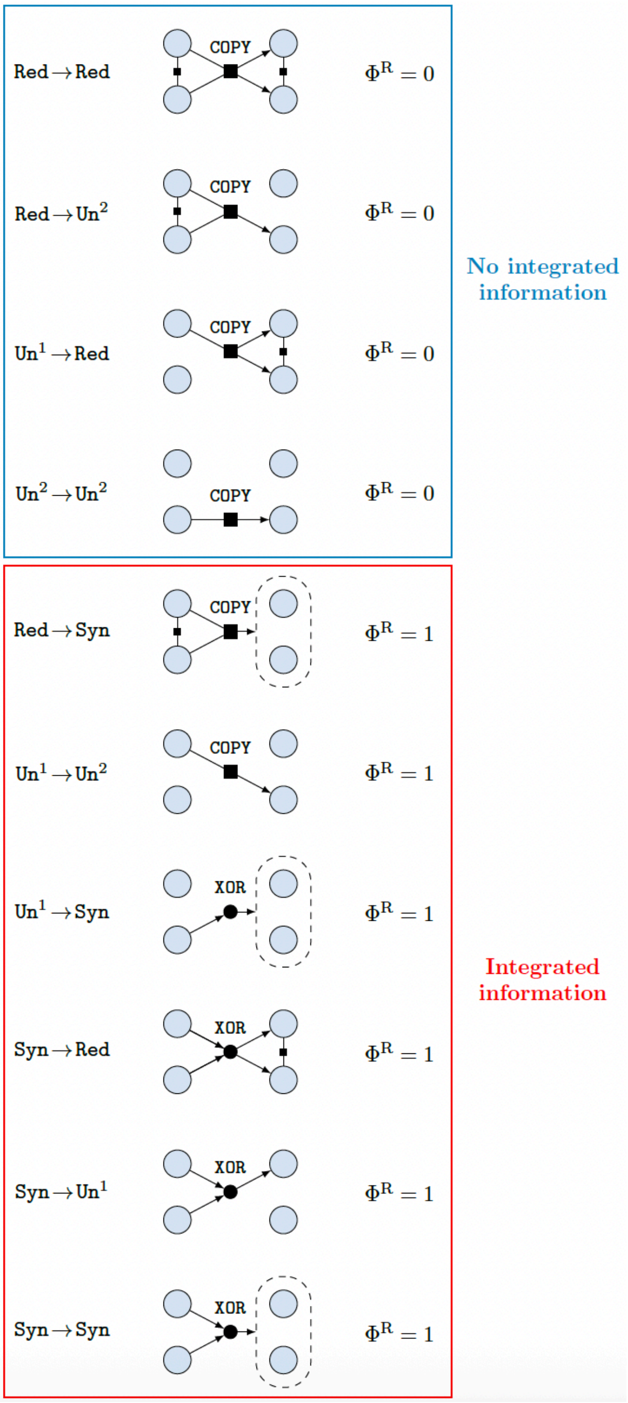

Figure S1. Elementary examples of information-dynamic atoms | Each 2-element system displays one and only one of the 16 information-dynamic atoms (cases where Un1 and Un2 would be interchangeable are only shown for one of them). We also show how much integrated information (Φ_R) would be exhibited by each system.

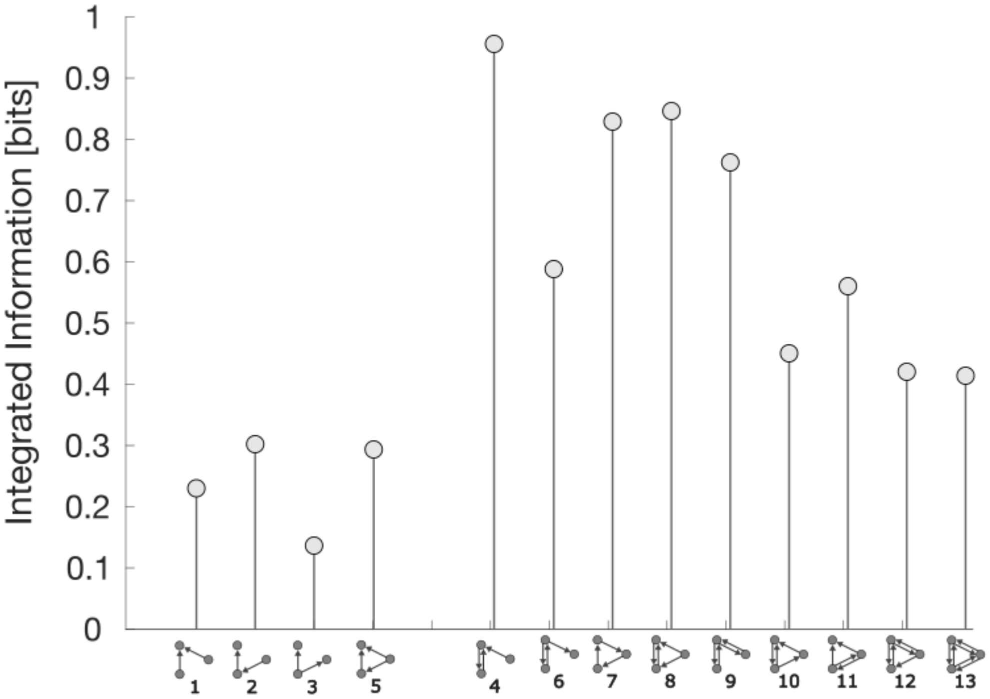

Figure S2. Integrated information (Φ_R) for each of the 13 possible 3-node motifs | We find higher Φ_R for motifs exhibiting recurrent connectivity, whether direct (i.e., reciprocal connections between two nodes) or indirect (i.e., a 3-node cycle). Color follows conventional motif number from (Sporns and Kötter, 2004).

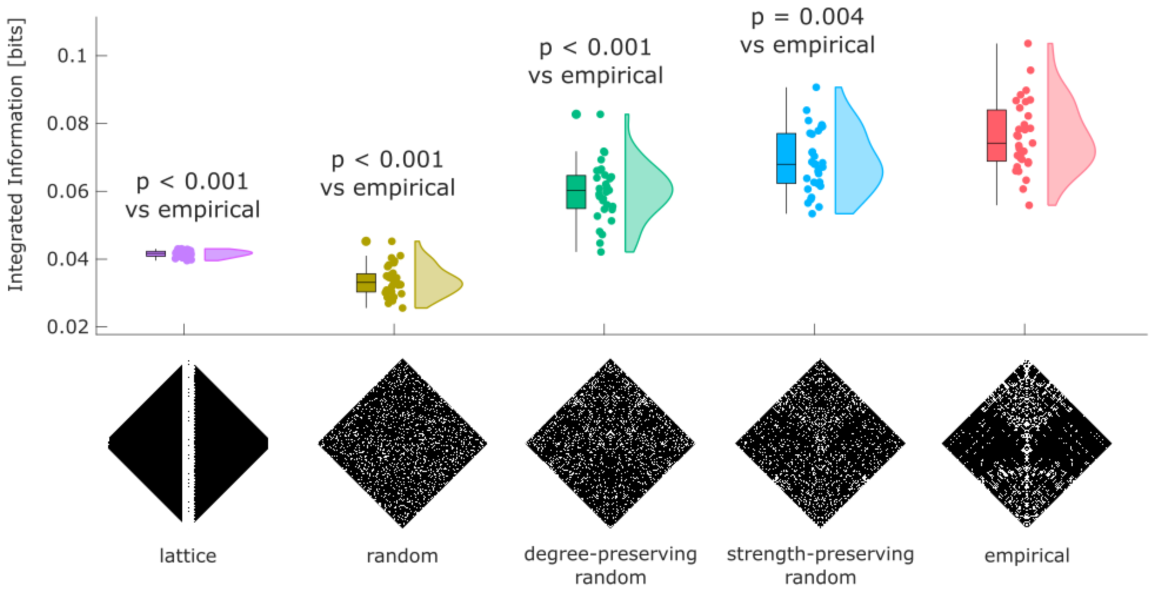

Figure S3. Integrated information (Φ_R) for connectome-based brain models based on different network topologies | All null networks preserve the network size, density, and weight distribution of the empirical human connectome reconstructed from diffusion tractography, thereby only varying in their topology. From left to right: lattice network; fully random topology; random but preserving the empirical degree (number of connections) of each node; random but preserving the empirical degree and also the empirical strength (sum of connections' weights) of each node; and finally the empirical human connectome. We find that integrated information (Φ_R) is significantly higher for the empirical human connectome than for each of the null networks. N = 31 simulations for each condition P-values are from independent-samples t-tests (two-sided). Box plots: central line, median; box limits, upper and lower quartiles; whiskers, 1.5× interquartile range.

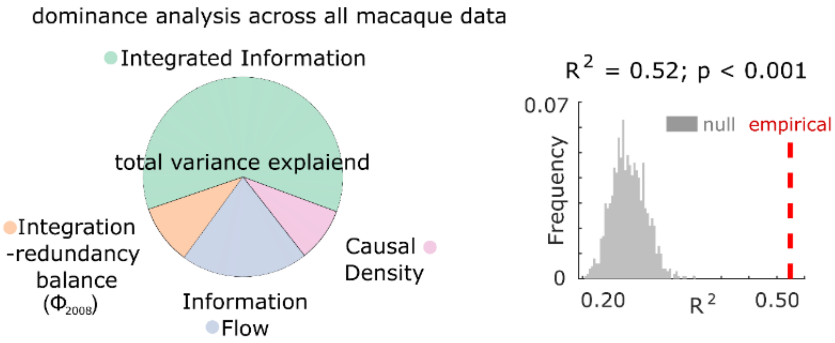

Figure S4. Comparing functional brain markers' ability to account for changes in arousal scores induced by anaesthesia and thalamic deep brain stimulation | Regression target is the behavioural arousal score from the combined Multi-Anaesthesia and DBS macaque datasets, measured on the same preclinical behavioural scale (Tasserie et al., 2022; Uhrig et al., 2018). Percentage of relative importance is represented as a pie chart, revealing that the Φ_R measure of integrated information is the predictor with highest relative importance, accounting for 61% of the total variance explained. We establish the statistical significance of the multiple linear regression model accounting for arousal score as a function of our fMRI measures model using a non-parametric permutation test (one-sided), by comparing the empirical variance explained against a null distribution of R^2 obtained from repeating the multiple regression with randomly reassigned arousal scores. The empirical variance explained is significantly greater than chance ($R^2 = 0.52$; p < 0.001).

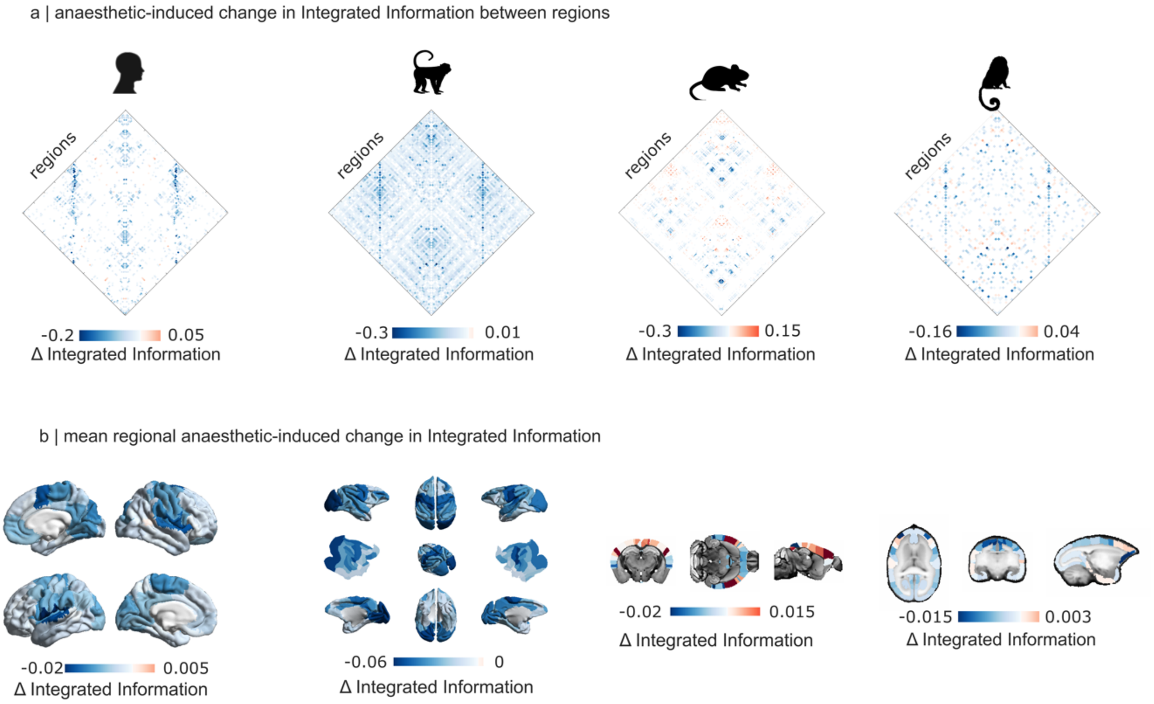

Figure S5. Localised change in integrated information across species | (**a**) For each species, in each condition the Integrated Information between each pair of regions is obtained from Information Decomposition of the fMRI signal time-series. The resulting functional networks are then compared using the Network-Based Statistic (Zalesky et al., 2010). For the human dataset, we compare sevoflurane anaesthesia versus wakefulness and versus recovery. For the macaque multi-anaesthesia dataset, we compare wakefulness against deep anaesthesia with propofol, sevoflurane, or ketamine. For the macaque DBS dataset, we compare propofol anaesthesia against wakefulness, and against high- and low-intensity stimulation of the central thalamus, which both restored behavioural markers of arousal. For the mouse dataset, we compare wakefulness against halothane and medetomidine+isoflurane. For the marmoset dataset, we compare wakefulness against propofol, isoflurane, and sevoflurane. The results are averaged within species to obtain edge-level results for each species. (**b**) Mean regional changes in Integrated information for each cortical region. Credits: Human head icon from pixabay.com. Mouse icon designed by CraftStarters.com. Macaque icon designed by Freepik.com. Marmoset icon from pixabay.com.

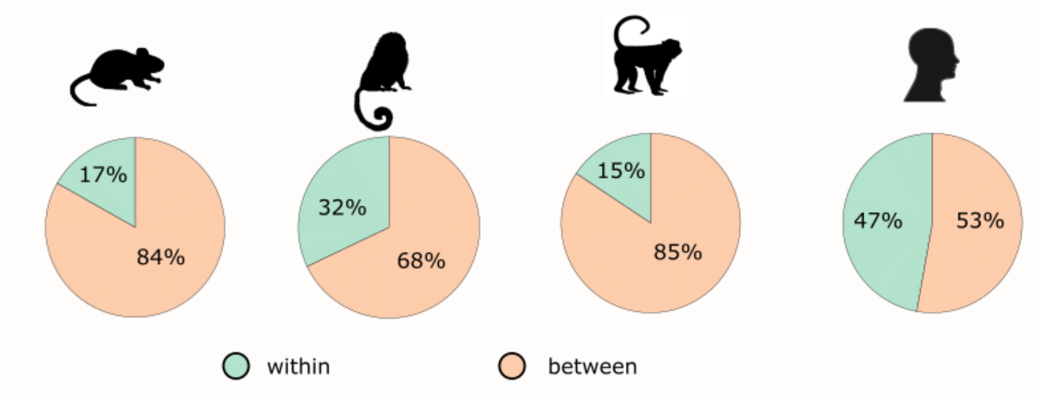

Figure S6. Proportion of NBS-corrected cortical changes in integrated information that occur within or between different functional systems (averaged across contrasts). Credits: Human head icon from pixabay.com. Mouse icon designed by CraftStarters.com. Macaque icon designed by Freepik.com. Marmoset icon from pixabay.com.

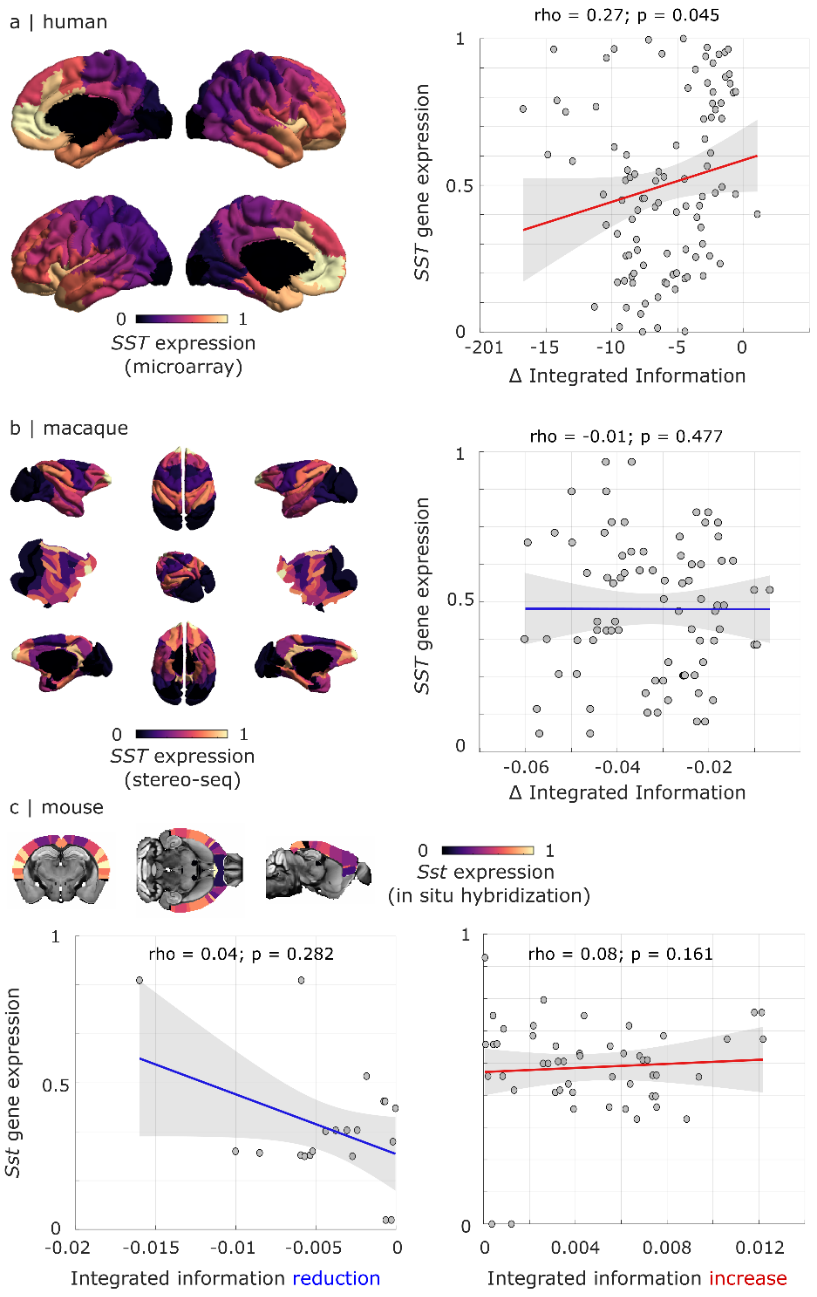

Figure S7. Correlations between regional loss of integrated information under anaesthesia and the regional distribution of *SST/Sst* gene expression | (a) Regionally-defined transcriptomic data for n = 100 human cortical regions were obtained from the Allen Institute for Brain Science database. (b) Regionally-defined transcriptomic data for n = 80 macaque cortical regions were made available by the Brain Science Data Center of the Chinese Academy of Sciences. (c) Regionally-defined transcriptomic data for n = 72 human cortical regions were obtained from the Allen Institute for Brain Science database. The statistical significance of correlations (Spearman's *rho*) was assessed against spatial autocorrelation-preserving null models implemented with Moran spectral randomisation (Methods). Shading indicates 95% confidence intervals.

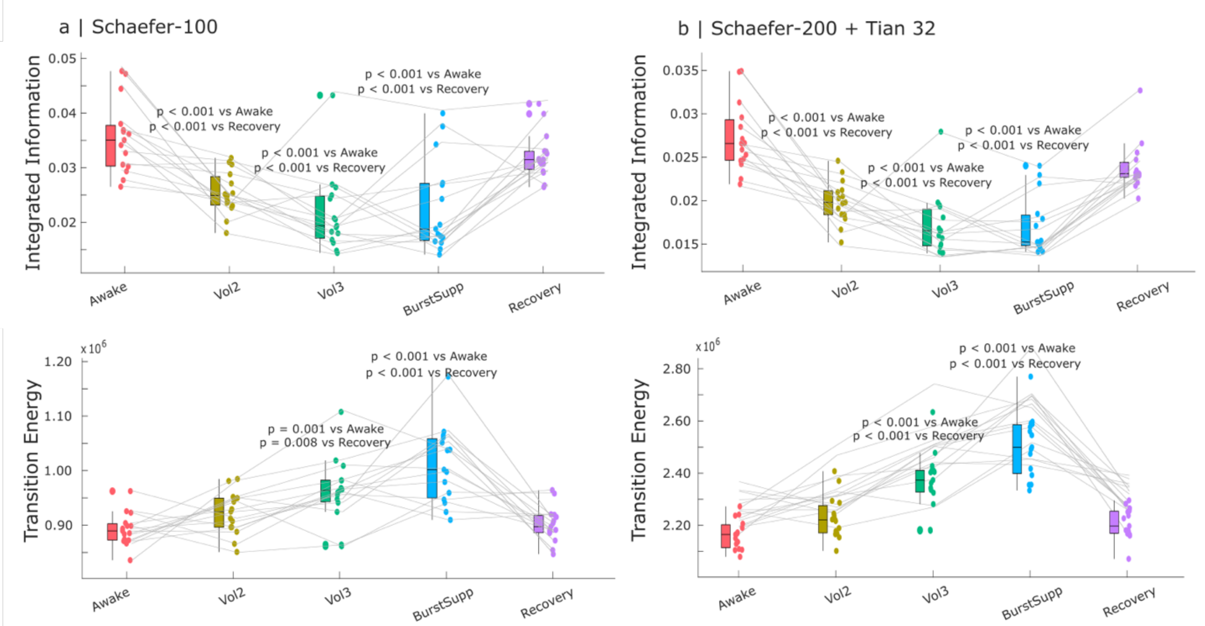

Figure S8. Replication of human results across different doses of sevoflurane and a different parcellation | (a) Integrated information (top) and transition energy (bottom), for Schaefer-100 cortical atlas; n = 15 human volunteers for each condition. Box plots: central line, median; box limits, upper and lower quartiles; whiskers, 1.5× interquartile range. (**b**) Integrated information (top) and transition energy (bottom), for Schaefer-200 cortical atlas with 32 subcortical regions from Tian atlas; n = 15 human volunteers for each condition. P-values from paired-samples t-tests, two-sided, FDR-corrected for multiple comparisons against Awake condition, and against Recovery condition. Box plots: central line, median; box limits, upper and lower quartiles; whiskers, 1.5× interquartile range. Vol2, 2% vol sevoflurane; Vol3, 3% vol sevoflurane; BurstSupp, burst-suppression.

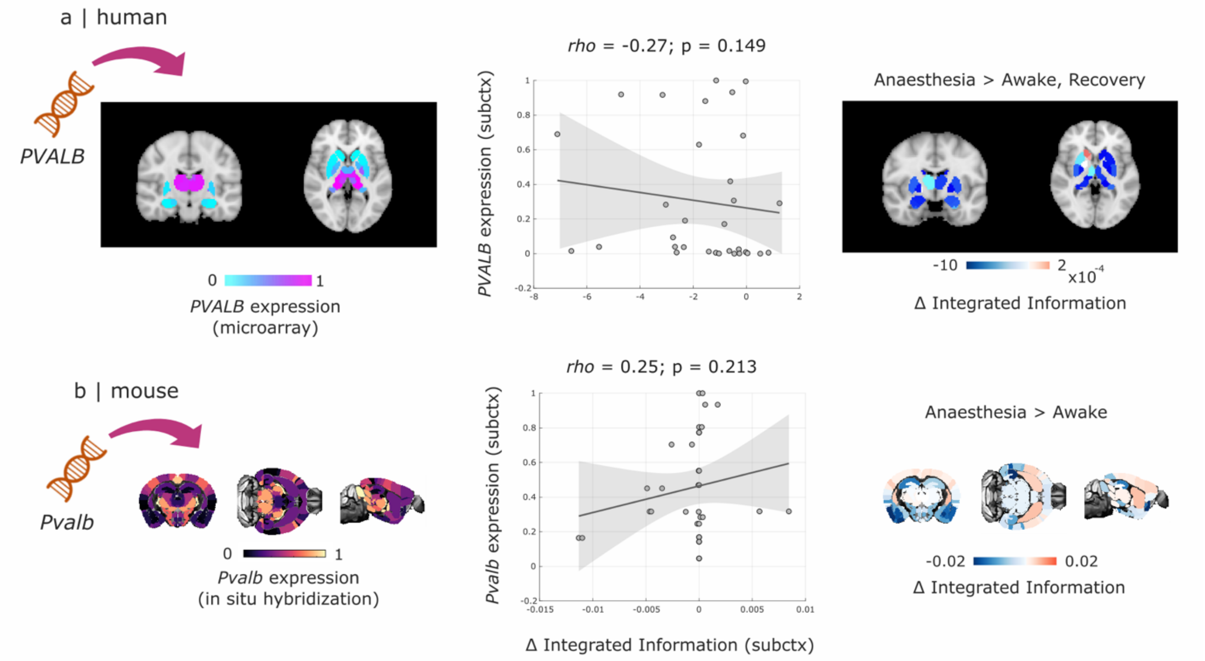

Figure S9. Correlations between subcortical loss of integrated information under anaesthesia and the subcortical distribution of *PVALB/Pvalb* gene expression | (**a**) Human microarray data from the Allen Institute database (n = 32 subcortical regions). (**b**) Mouse in situ hybridization data from the Allen Institute database (n = 34 subcortical regions). Note that some subcortical regions show no significant changes in integrated information. Spatial correlation is quantified as Spearman's *rho,* with shading indicating 95% confidence interval.

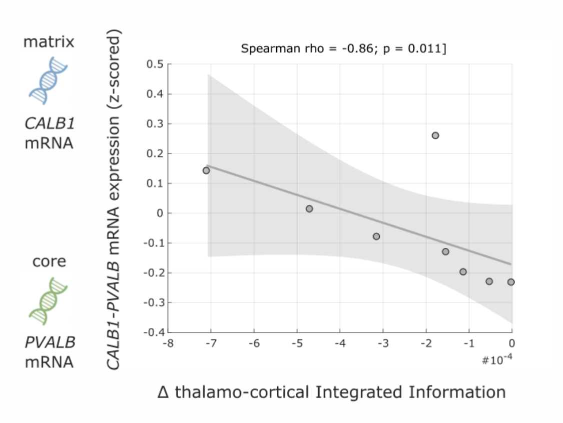

Figure S10. Relationship between anaesthetic-induced loss of thalamo-cortical integrated information and transcriptomically-defined core-matrix architecture of the human thalamus | Core-matrix architecture (y-axis) for human thalamic nuclei is defined transcriptomically from the relative mRNA expression of *CALB1* and *PVALB,* such that greater relative *CALB1* expression denotes matrix nuclei, and greater relative *PVALB* expression denotes core nuclei, as per (Huang et al., 2024; Müller et al., 2020). Spatial correlation across n = 8 thalamic nuclei is quantified as Spearman's *rho,* with shading indicating 95% confidence interval.

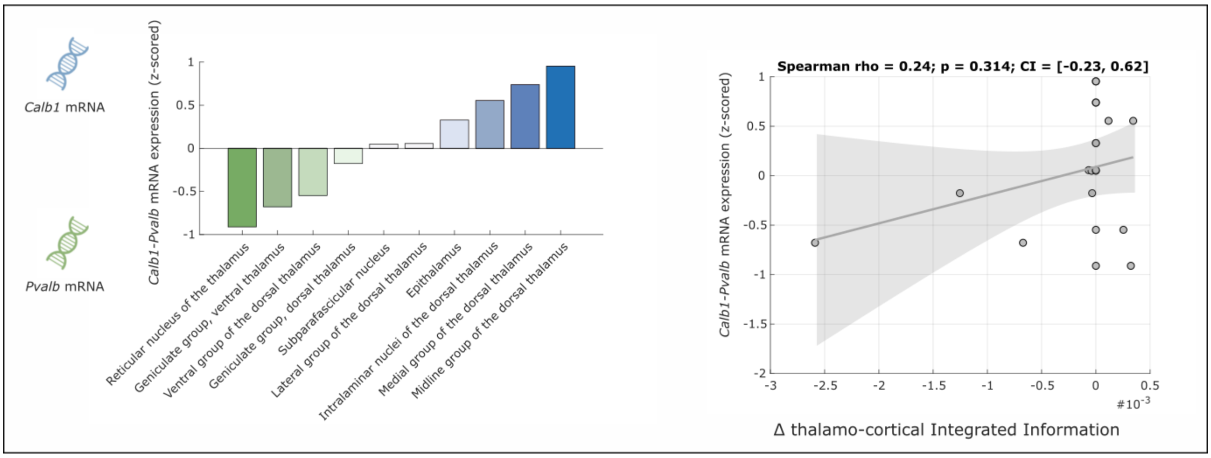

Figure S11. Relationship between anaesthetic-induced change in thalamo-cortical integrated information and transcriptomically-defined core-matrix architecture of the mouse thalamus | Core-matrix architecture (y-axis) for mouse thalamic nuclei is defined transcriptomically from the relative mRNA expression of *Calb1* and *Pvalb,* such that greater relative *Calb1* expression denotes matrix nuclei, and greater relative *Pvalb* expression denotes core nuclei, as per (Huang et al., 2024; Müller et al., 2020). Spatial correlation across n = 20 thalamic nuclei is quantified as Spearman's *rho,* with shading indicating 95% confidence interval.

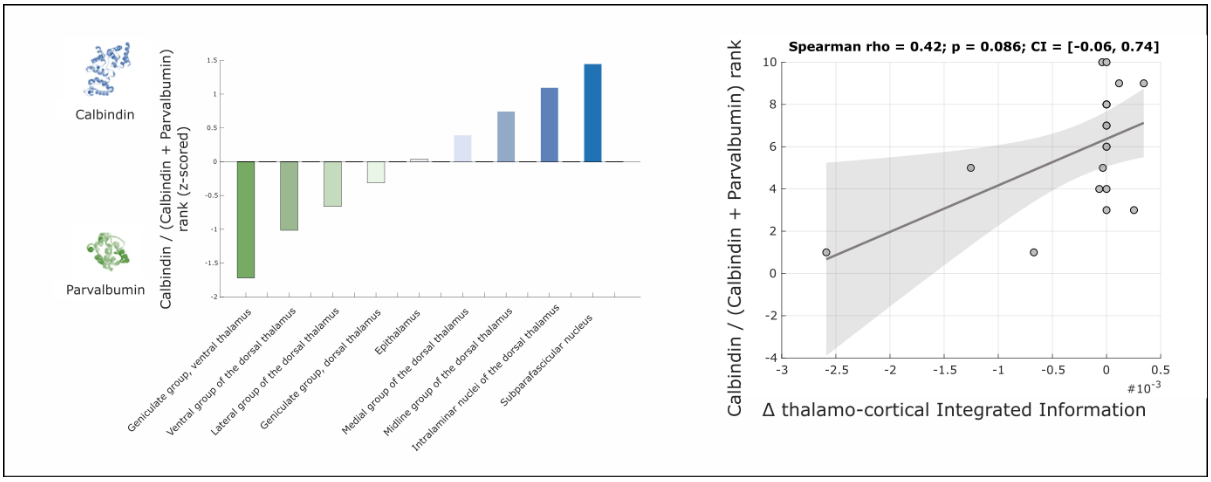

Figure S12. Relationship between anaesthetic-induced change in thalamo-cortical integrated information and core-matrix architecture of the mouse thalamus defined from immunohistochemistry | Core-matrix architecture (y-axis) for mouse thalamic nuclei is defined from the relative protein density of calbindin (the protein coded for by *Calb1*) and parvalbumin (the protein coded for by *Pvalb*), as quantified from immunohistochemistry(Bjerke et al., 2021), such that greater relative calbindin density vis-à-vis parvalbumin denotes more matrix-like nuclei. Spatial correlation across n = 18 thalamic nuclei is quantified as Spearman's *rho,* with shading indicating 95% confidence interval. Note that Bjerke and colleagues did not provide data for the reticular nucleus of the thalamus, because this region was oversaturated with staining. Credits: protein icons adapted from Huang et al (Huang et al., 2024), published under CC-BY license.

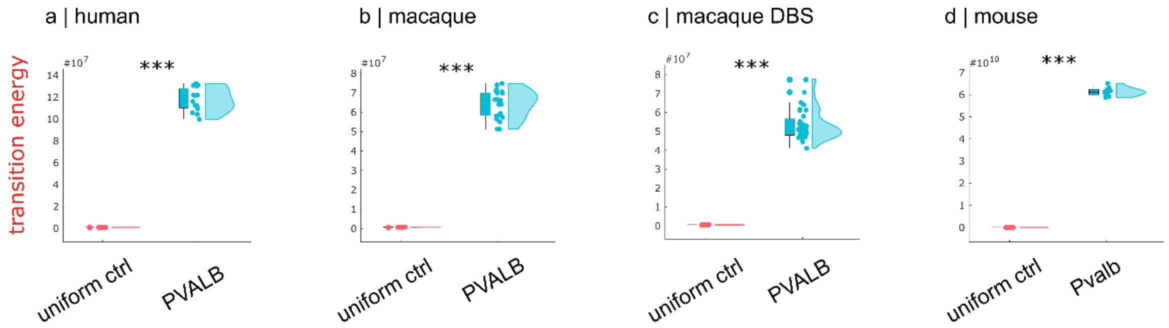

Figure S13. Regional reductions of control input increase the overall control cost of brain dynamics compared with uniform control | (**a**) Human dataset (n = 15). (**b**) Macaque multi-anaesthesia dataset (n = 24 runs from 3 animals). (**c**) Macaque DBS dataset (n = 36 runs from 3 animals). (**d**) Mouse dataset (n = 10). P-values are from non-parametric paired-samples test (two-sided). Box plots: central line, median; box limits, upper and lower quartiles; whiskers, 1.5× interquartile range.

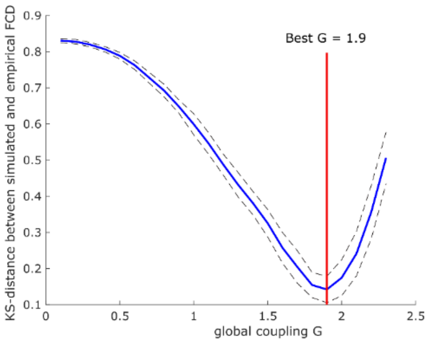

Figure S14. Dynamic mean-field model fitting | The global coupling parameter *G* is tuned to find the value where the model-simulated functional connectivity dynamics (FCD) exhibit the least KS distance from the empirical FCD of the anaesthetised macaque.

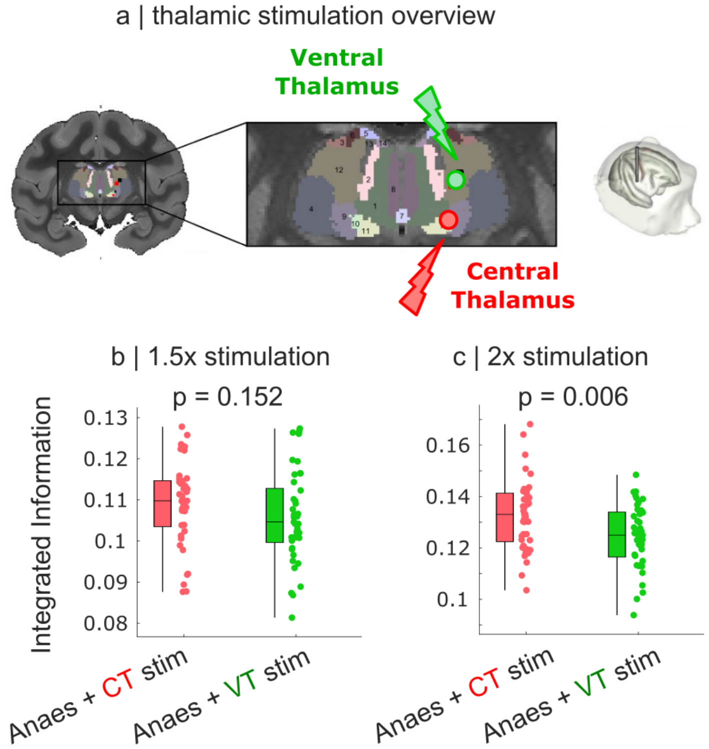

Figure S15. Difference in integrated information for macaque-specific biophysical model with stimulation of different thalamic nuclei depends on stimulation intensity | (**a**) Illustration of thalamic ROIs and their inclusion in the DMF model; adapted from Tasserie *et al.* (Tasserie et al., 2022), published under CC-BY license. Models based on the macaque anatomical connectivity, fitted to the empirical anaesthesia condition, are subjected to injection of excitatory current based on the structural connectivity of the central thalamus (CT; red) and ventral lateral thalamus (VT; green), obtained from diffusion-weighted MRI tractography of an independent sample of macaques. (**b**) No significant difference in integrated information between CT and VT for model with 1.5x scaling of excitatory input. P-value from non-parametric independent-samples test (two-sided). N = 41 simulations for each condition. Box plots: central line, median; box limits, upper and lower quartiles; whiskers, 1.5× interquartile range. (**c**) At 2x scaling of excitatory input, both CT and VT stimulation induce greater integrated information than at 1.5x scaling; however, a significantly greater effect is now also observed for CT than VT stimulation. P-value from non-parametric independent-samples test (two-sided). N = 41 simulations for each condition. Box plots: central line, median; box limits, upper and lower quartiles; whiskers, 1.5× interquartile range.

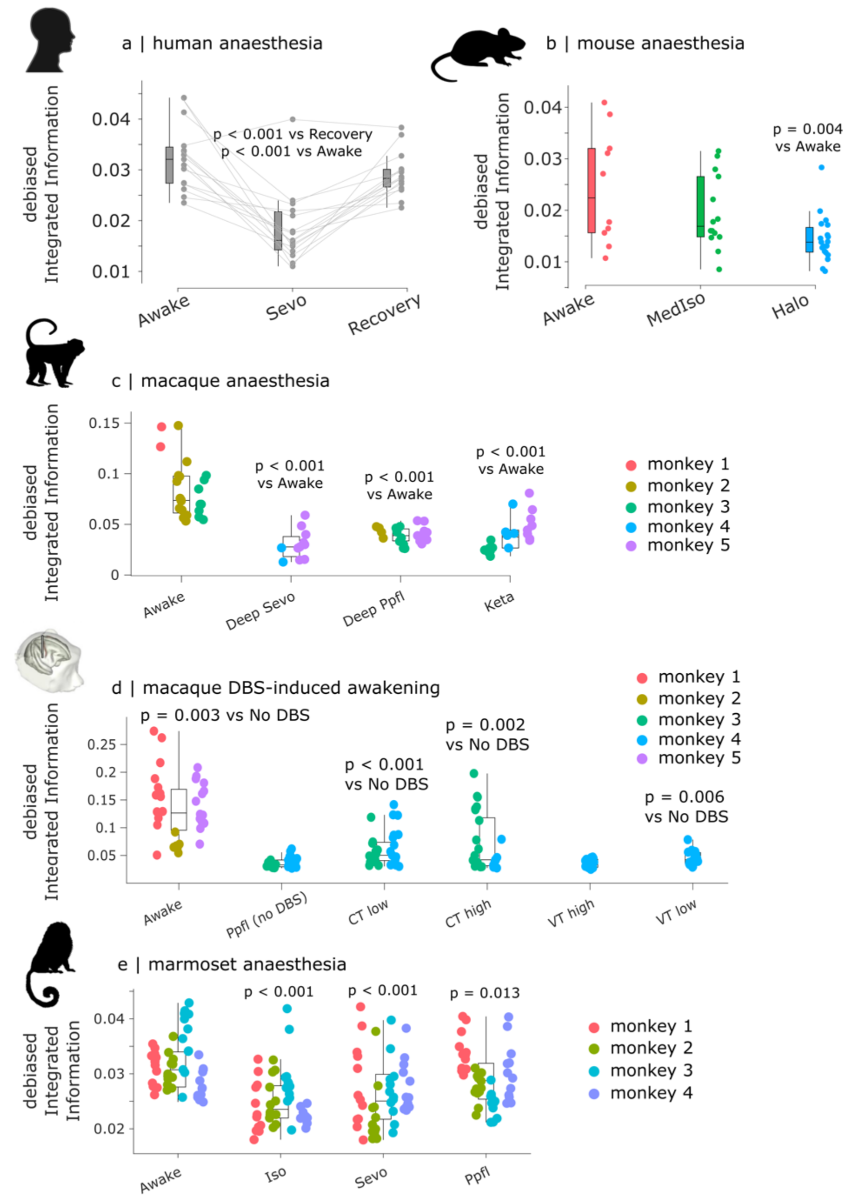

Figure S16. Integrated information results are not driven by estimation bias | (**a**) Human (n = 15) sevoflurane anaesthesia (Sevo) versus wakefulness ($p < 0.001$ from two-sided paired-samples t-test, FDR-corrected for multiple comparisons against Awake condition) and recovery ($p < 0.001$ from two-sided paired-samples t-test, FDR-corrected for multiple comparisons against Recovery condition). Box plots: central line, median; box limits, upper and lower quartiles; whiskers, 1.5× interquartile range. (**b**) Mouse wakefulness (n=10) versus medetomidine-isoflurane (MedIso; n = 14) and halothane (Halo; n = 19) anaesthesia data. P-values are from two-sided independent-samples t-test, FDR-corrected for multiple comparisons against Awake condition. Box plots: central line, median; box limits, upper and lower quartiles; whiskers, 1.5× interquartile range. (**c**) Macaque wakefulness versus anaesthesia with propofol (Ppfl), sevoflurane (Sevo), and ketamine (Keta). N = 24 runs from 3 animals for Awake; 11 runs from 2 animals for Sevoflurane; 23 runs from 3 animals for Propofol; 22 runs from 3 animals for Ketamine anaesthesia. P-values are from linear mixed effects models (two-sided), FDR-corrected for multiple comparisons against Awake condition (see Methods). Box plots: central line, median; box limits, upper and lower quartiles; whiskers, 1.5× interquartile range. (**d**) Macaque DBS stimulation. N = 36 runs from 3 animals for Awake; 28 runs from 2 animals for anaesthesia (DBS-off); 31 runs from 2 animals for low amplitude centro-median thalamic DBS; 25 runs from 2 animals for high amplitude centro-median thalamic DBS; 18 runs from 1 animal for low amplitude ventro-lateral thalamic DBS; 18 runs from 1 animal for high amplitude ventro-lateral thalamic DBS. P-values are from linear mixed effects models (two-sided), FDR-corrected for multiple comparisons against propofol anaesthesia with no DBS (see Methods). Box plots: central line, median; box limits, upper and lower quartiles; whiskers, 1.5× interquartile range. Data-points with the same colour indicate the same animal. (**e**) Marmoset

wakefulness versus anaesthesia with isoflurane (Iso), sevoflurane (Sevo) and propofol (Ppfl). N = 48 runs from 4 animals for each condition. P-values are from linear mixed effects models (two-sided), FDR-corrected for multiple comparisons against Awake condition (see Methods). Box plots: central line, median; box limits, upper and lower quartiles; whiskers, 1.5× interquartile range. Data-points with the same colour indicate the same animal. See Supplementary Tables S6-S10 for full statistical reporting. Credits: Human head icon from pixabay.com. Mouse icon designed by CraftStarters.com. Macaque icon designed by Freepik.com. DBS and macaque thalamus illustrations adapted from Tasserie et al (2022), published under CC-BY license. Marmoset icon from pixabay.com.

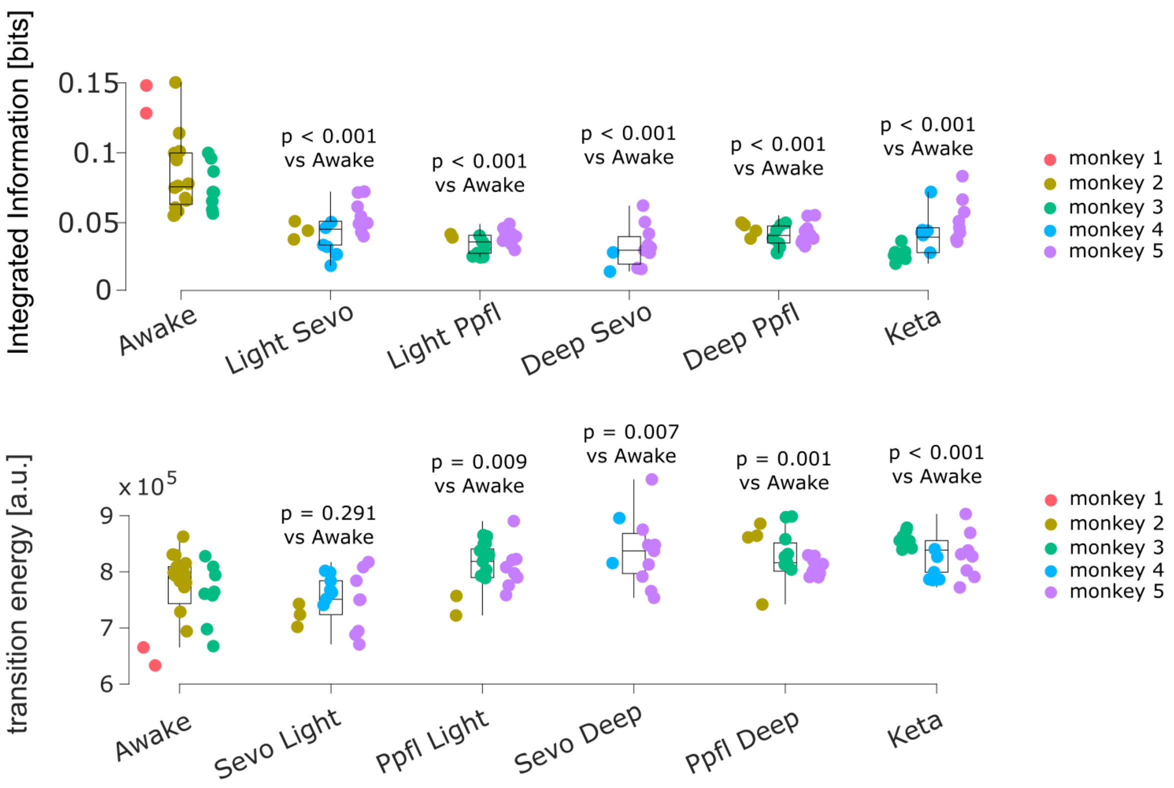

Figure S17. Replication of macaque results across different doses of sevoflurane and propofol | Integrated information (top) and transition energy (bottom). P-values are from linear mixed effects models (two-sided), FDR-corrected for multiple comparisons. Box-plots indicate the median and inter-quartile range of the data distributions. Within each panel, data-points of the same color refer to the same animal.

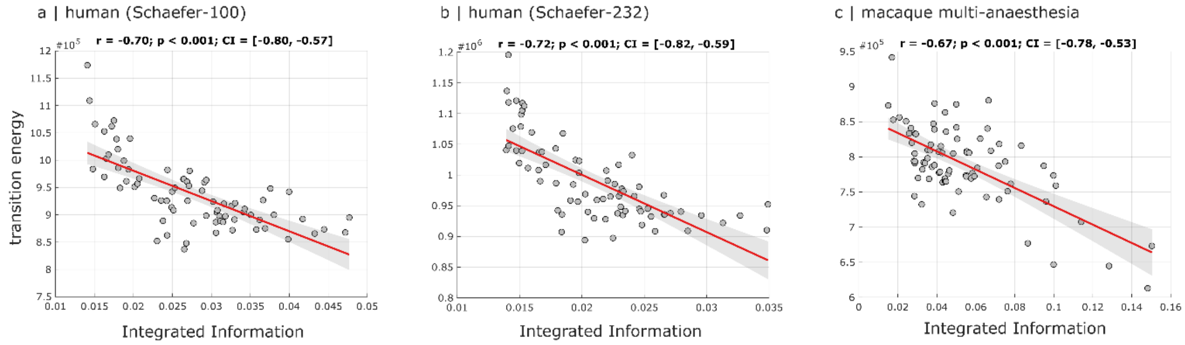

Figure S18. Negative Pearson correlations between integrated information and transition energy remain significant when including all anaesthetic doses | (**a**) Human dataset (Schaefer-100 atlas). N = 15 human volunteers for each of 5 conditions. Shading indicates 95% confidence interval. (**b**) Human dataset (Schaefer-200 + Tian-32 subcortical atlas). N = 15 human volunteers for each of 5 conditions. Shading indicates 95% confidence interval. (**c**) Macaque multi-anaesthesia dataset. N = 24 runs from 3 animals for Awake; 18 runs from 3 animals for Light Sevoflurane; 21 runs from 3 animals for Light Propofol; 11 runs from 2 animals for Deep Sevoflurane; 23 runs from 3 animals for Deep Propofol; 22 runs from 3 animals for Ketamine anaesthesia. Shading indicates 95% confidence interval.

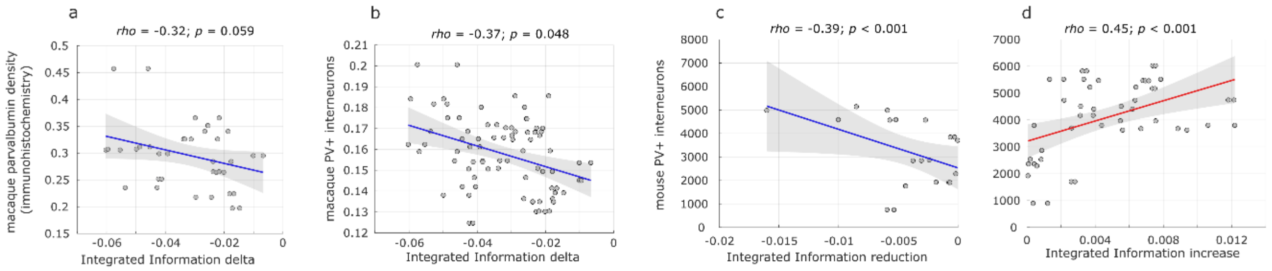

Figure S19. Validation of correlations between integrated information change and parvalbumin expression | (**a**) Negative correlation between loss of integrated information and parvalbumin protein density across n = 38 regions of the macaque cortex with available data, as obtained from immunoihistochemistry (compiled by Burt et al., 2018). Spatial correlation is quantified as Spearman's *rho,* with shading indicating 95% confidence interval. (**b**) Negative correlation between loss of integrated information, and density of transcriptomically-defined PV+ interneurons across n = 80 regions of the macaque cortex with available data. Spatial correlation is quantified as Spearman's *rho,* with shading indicating 95% confidence interval. (**c**) Negative correlation between loss of integrated information and regional prevalence of PV+ interneurons across n = 22 regions of the mouse cortex, as quantified by Kim, Yang and colleagues (Kim et al., 2017). Spatial correlation is quantified as Spearman's *rho,* with shading indicating 95% confidence interval. (**d**) Positive correlation between increase of integrated information and regional prevalence of PV+ interneurons across n = 48 regions of the mouse cortex, as quantified by Kim, Yang and colleagues (Kim et al., 2017). Spatial correlation is quantified as Spearman's *rho,* with shading indicating 95% confidence interval. The statistical significance of correlations was assessed against spatial autocorrelation-preserving null models (Methods).

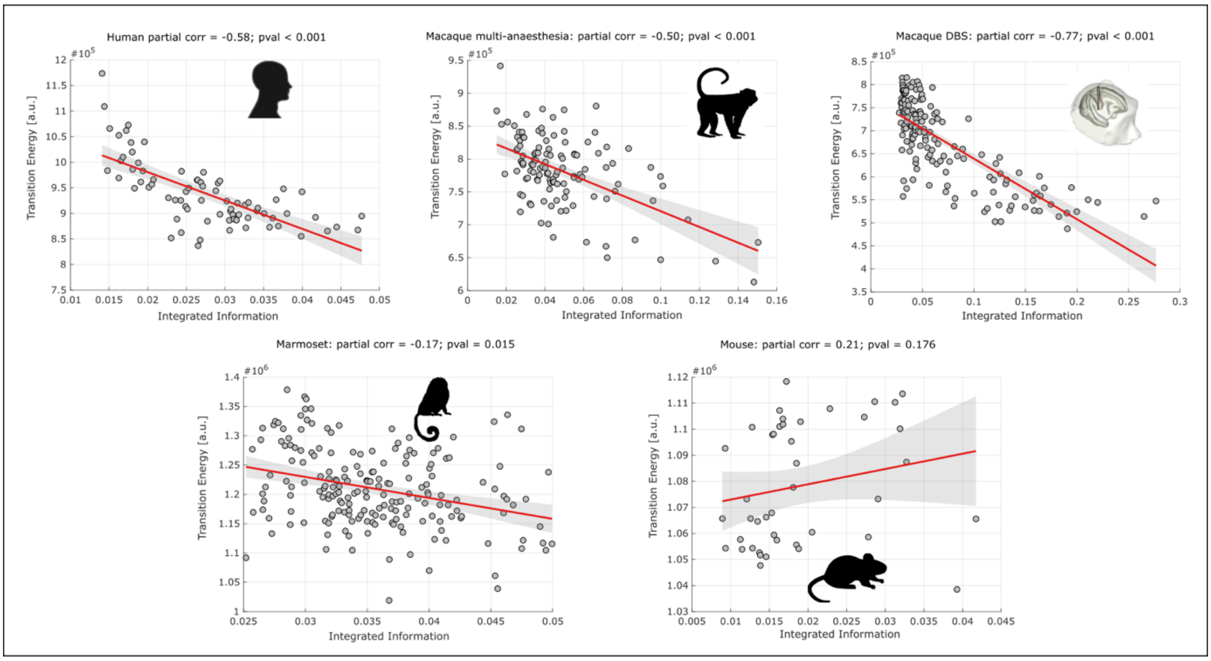

Figure S20. Partial correlation between transition energy and integrated information across all data-points, within each dataset, after partialling out the effect of mean framewise displacement | Human dataset: n = 15 human volunteers for each of 5 conditions. Macaque multi-anaesthesia dataset: n = 24 runs from 3 animals for Awake; 18 runs from 3 animals for Light Sevoflurane; 21 runs from 3 animals for Light Propofol; 11 runs from 2 animals for Deep Sevoflurane; 23 runs from 3 animals for Deep Propofol; 22 runs from 3 animals for Ketamine anaesthesia. Macaque DBS dataset: n = 36 runs from 3 animals for Awake; 28 runs from 2 animals for anaesthesia (DBS-off); 31 runs from 2 animals for low amplitude centro-median thalamic DBS; 25 runs from 2 animals for high amplitude centro-median thalamic DBS; 18 runs from 1 animal for low amplitude ventro-lateral thalamic DBS; 18 runs from 1 animal for high amplitude ventro-lateral thalamic DBS. Marmoset dataset: n = 12 runs from each of 4 animals, for each condition (Awake, Propofol, Isoflurane, Sevoflurane). Mouse dataset: n=10 for awake, n = 14 for medetomidine-isoflurane anaesthesia and n = 19 for halothane. Shading indicates 95% confidence interval (two-sided). Credits: Human head icon from pixabay.com. Mouse icon designed by CraftStarters.com. Macaque icon designed by Freepik.com. DBS illustration adapted from Tasserie et al (2022), published under CC-BY license. Marmoset icon from pixabay.com.

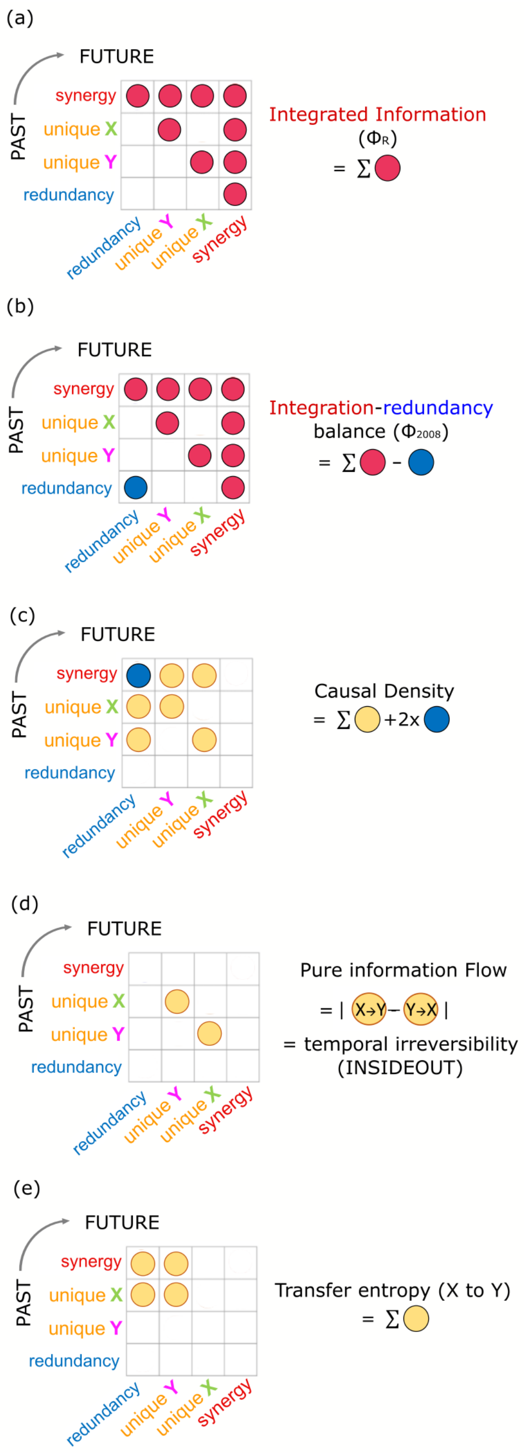

Figure S21. Information decomposition of key information-theoretic measures linked to consciousness research | (a) Revised measure of Integrated Information (Φ_R) corresponding to the whole minus the (proper) sum of the parts(Mediano et al., 2021). (b) Integration-redundancy balance, originally proposed as a measure of whole-minus-sum integrated information (Φ_{2008} from (Balduzzi and Tononi, 2008)), but using a naive sum of the parts that double-counts the persistent redundancy in the system. (c) Causal Density(Seth et al., 2011), the sum of transfer entropies from X to Y and from Y to X, which double-counts the synergy-to-redundancy atom as shown in [8]. (d) The 'pure' information flow, corresponding to the difference in magnitude between the pure transfer from X to Y, and the pure transfer from Y to X. For linear systems, (Luppi et al., 2023d) showed that this measure is mathematically equivalent to the INSIDEOUT measure of temporal irreversibility introduced in (Deco et al., 2022). (e) For completeness we also show the Transfer Entropy from X to Y, which includes the pure transfer from X to Y (information that was uniquely provided by X in the past, and is uniquely provided by Y in the future) but also includes synergistic effects and information duplication.

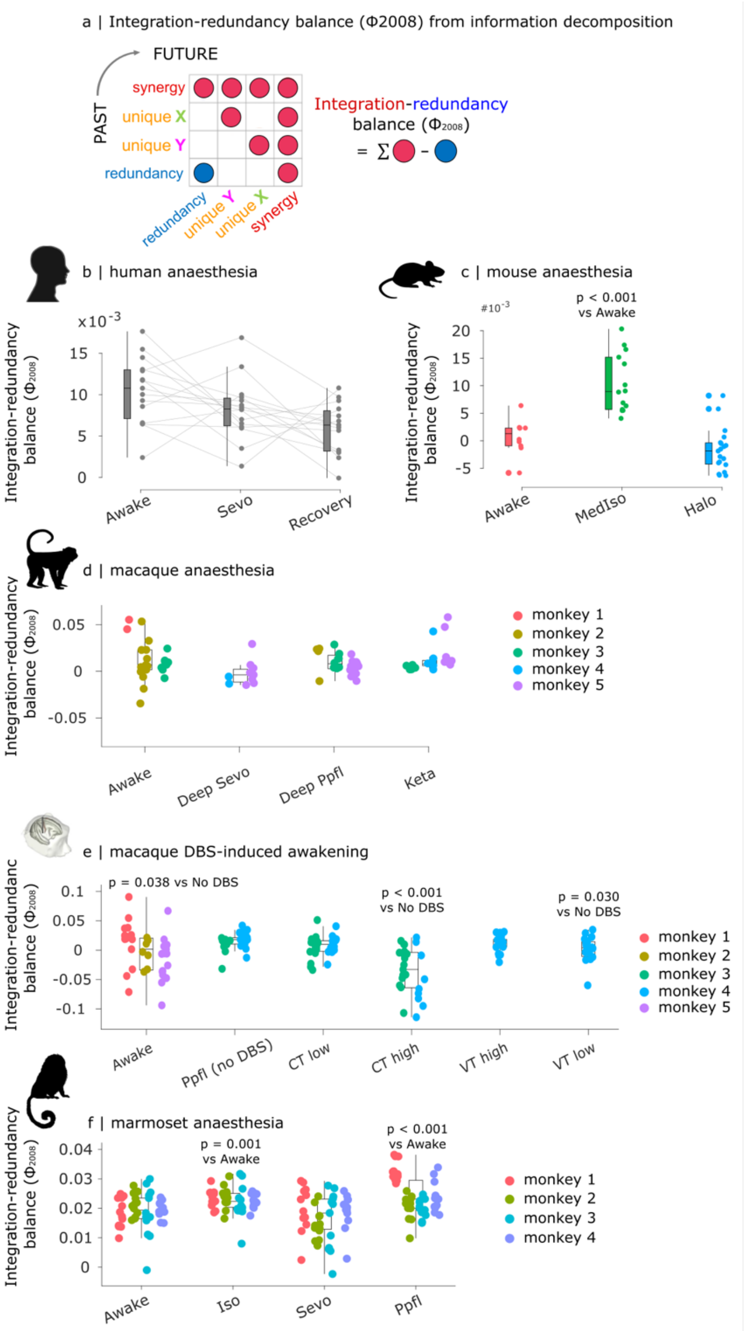

Figure S22. Φ_{2008} exhibits inconsistent results under anaesthesia | (**a**) Information-dynamic formulation of Tononi and Balduzzi's Φ_{2008}, as obtained from information decomposition, reveals that this measure quantifies the balance between integrated information and persistent redundancy (Mediano et al., 2021). (**b**) Human sevoflurane anaesthesia (Sevo) versus wakefulness (two-sided paired-samples t-test, FDR-corrected for multiple comparisons) and versus post-anaesthetic recovery (two-sided paired-samples t-test, FDR-corrected for multiple comparisons); n =15. Box plots: central line, median; box limits, upper and lower quartiles; whiskers, 1.5× interquartile range. (**c**) Mouse wakefulness (n=10) versus medetomidine-isoflurane (MedIso; n = 14) and halothane (Halo; n = 19) anaesthesia data. P-values are from two-sided independent-samples t-test, FDR-corrected for multiple comparisons against Awake condition. Box plots: central line, median; box limits, upper and lower quartiles; whiskers, 1.5× interquartile range. (**d**) Macaque wakefulness versus anaesthesia with propofol (Ppfl), sevoflurane (Sevo), and ketamine (Keta). N = 24 runs from 3 animals for Awake; 11 runs from 2 animals for Sevoflurane; 23 runs from 3 animals for Propofol; 22 runs from 3 animals for Ketamine anaesthesia. P-values are from linear mixed effects models (two-sided), FDR-corrected for multiple comparisons against Awake condition (see Methods). Box plots: central line, median; box limits, upper and lower quartiles; whiskers, 1.5× interquartile range. (**e**) Macaque DBS stimulation. N = 36 runs from 3 animals for Awake; 28 runs from 2 animals for anaesthesia (DBS-off); 31 runs from 2 animals for low amplitude centro-median thalamic DBS; 25 runs from 2 animals for high amplitude centro-

median thalamic DBS; 18 runs from 1 animal for low amplitude ventro-lateral thalamic DBS; 18 runs from 1 animal for high amplitude ventro-lateral thalamic DBS. P-values are from linear mixed effects models (two-sided), FDR-corrected for multiple comparisons against propofol anaesthesia with no DBS (see Methods). Box plots: central line, median; box limits, upper and lower quartiles; whiskers, 1.5× interquartile range. Data-points with the same colour indicate the same animal. (**f**) Marmoset wakefulness versus anaesthesia with isoflurane (Iso), sevoflurane (Sevo) and propofol (Ppfl). N = 48 runs from 4 animals for each condition. P-values are from linear mixed effects models (two-sided), FDR-corrected for multiple comparisons against Awake condition (see Methods). Box plots: central line, median; box limits, upper and lower quartiles; whiskers, 1.5× interquartile range. Data-points with the same colour indicate the same animal. See Tables S18-S22 for full statistical reporting. Credits: Human head icon from pixabay.com. Mouse icon designed by CraftStarters.com. Macaque icon designed by Freepik.com. DBS and macaque thalamus illustrations adapted from Tasserie et al (2022), published under CC-BY license. Marmoset icon from pixabay.com.

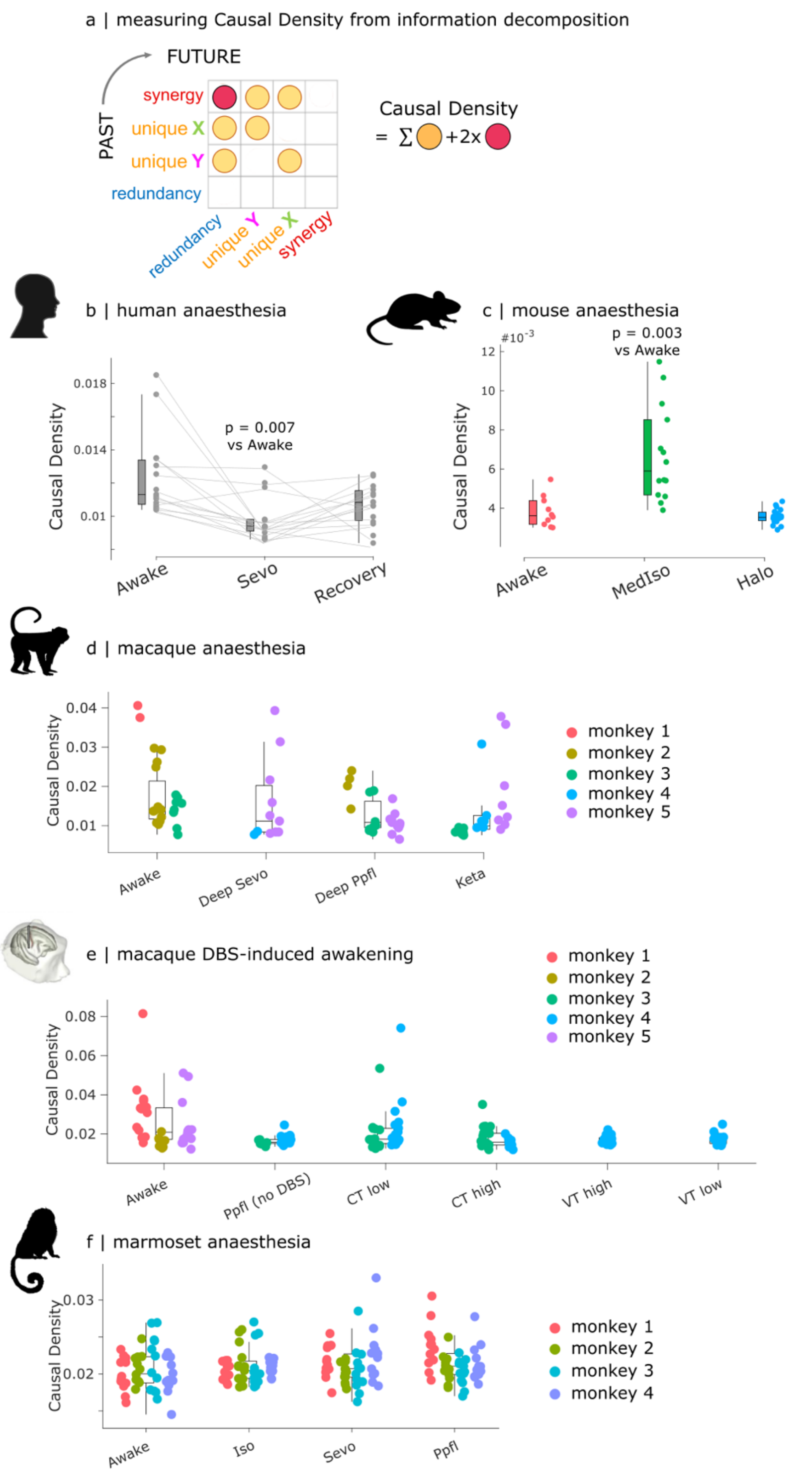

Figure S23. Inconsistent changes in Causal Density across species as a function of anaesthetic state | (**a**) Information-dynamic formulation of Causal Density, as obtained from information decomposition (Mediano et al., 2021). (**b**) Human sevoflurane anaesthesia (Sevo) versus wakefulness (two-sided paired-samples t-test, FDR-corrected for multiple comparisons) and versus post-anaesthetic recovery (two-sided paired-samples t-test, FDR-corrected for multiple comparisons); n =15. Box plots: central line, median; box limits, upper and lower quartiles; whiskers, 1.5× interquartile range. (**c**) Mouse wakefulness (n=10) versus medetomidine-isoflurane (MedIso; n = 14) and halothane (Halo; n = 19) anaesthesia data. P-values are from two-sided independent-samples t-test, FDR-corrected for multiple comparisons against Awake condition. Box plots: central line, median; box limits, upper and lower quartiles; whiskers, 1.5× interquartile range. (**d**) Macaque wakefulness versus anaesthesia with propofol (Ppfl), sevoflurane (Sevo), and ketamine (Keta). N = 24 runs from 3 animals for Awake; 11 runs from 2 animals for Sevoflurane; 23 runs from 3 animals for Propofol; 22 runs from 3 animals for Ketamine anaesthesia. P-values are

from linear mixed effects models (two-sided), FDR-corrected for multiple comparisons against Awake condition (see Methods). Box plots: central line, median; box limits, upper and lower quartiles; whiskers, 1.5× interquartile range. (**e**) Macaque DBS stimulation. N = 36 runs from 3 animals for Awake; 28 runs from 2 animals for anaesthesia (DBS-off); 31 runs from 2 animals for low amplitude centro-median thalamic DBS; 25 runs from 2 animals for high amplitude centro-median thalamic DBS; 18 runs from 1 animal for low amplitude ventro-lateral thalamic DBS; 18 runs from 1 animal for high amplitude ventro-lateral thalamic DBS. P-values are from linear mixed effects models (two-sided), FDR-corrected for multiple comparisons against propofol anaesthesia with no DBS (see Methods). Box plots: central line, median; box limits, upper and lower quartiles; whiskers, 1.5× interquartile range. Data-points with the same colour indicate the same animal. (**f**) Marmoset wakefulness versus anaesthesia with isoflurane (Iso), sevoflurane (Sevo) and propofol (Ppfl). N = 48 runs from 4 animals for each condition. P-values are from linear mixed effects models (two-sided), FDR-corrected for multiple comparisons against Awake condition (see Methods). Box plots: central line, median; box limits, upper and lower quartiles; whiskers, 1.5× interquartile range. Data-points with the same colour indicate the same animal. See Tables S23-S27 for full statistical reporting. Credits: Human head icon from pixabay.com. Mouse icon designed by CraftStarters.com. Macaque icon designed by Freepik.com. DBS and macaque thalamus illustrations adapted from Tasserie et al (2022), published under CC-BY license. Marmoset icon from pixabay.com.

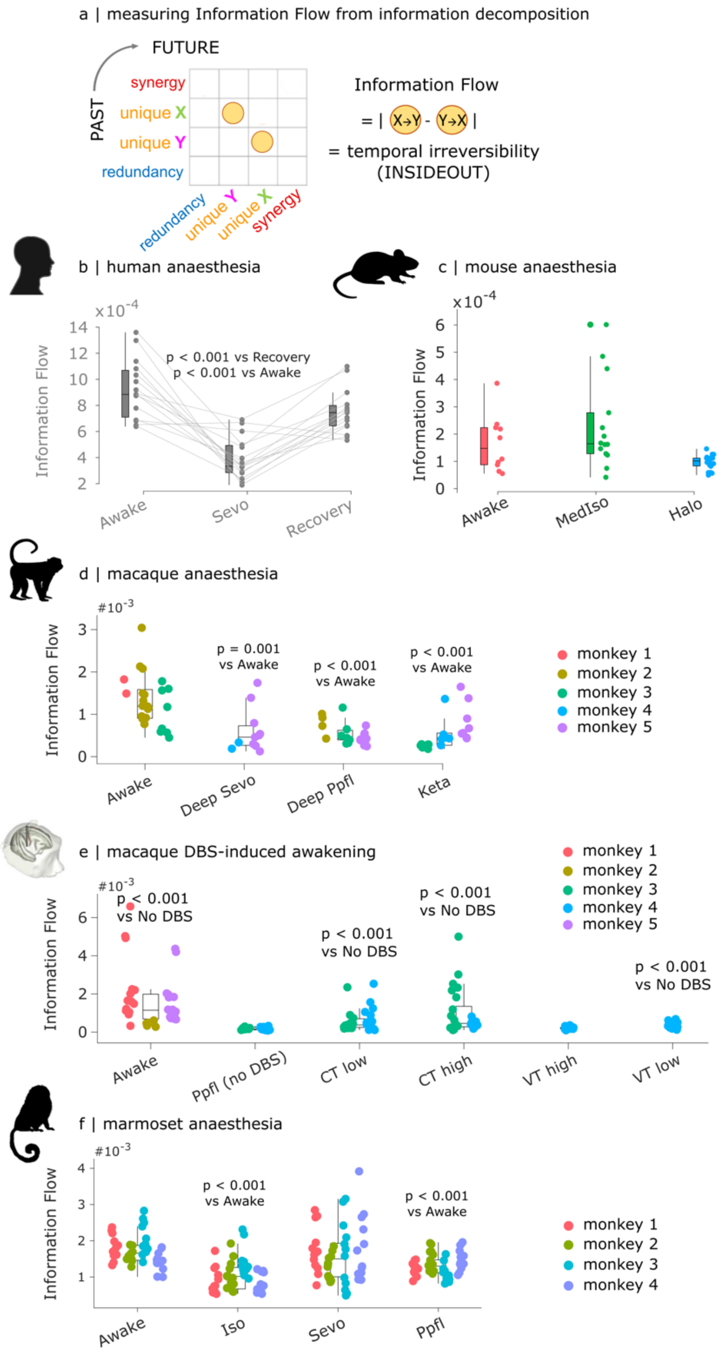

Figure S24. Changes in net information flow across species as a function of anaesthetic state | (**a**) The net information flow can be obtained from Information Decomposition of the FMRI time-series between pairs of regions, as the sum of the absolute difference between information that was in region X and is then in region Y, and the information that was in region Y and is then in region X (Luppi et al., 2023d). These two information-dynamic terms correspond to the "pure transfer" between X and Y (disregarding any synergistic phenomena, or duplication of information). Information flow is zero when the two terms are equal, i.e. X and Y are in balance, and positive otherwise, reflecting the presence of a net flow of information between the two. A single brain-wide value is then obtained by averaging across all regions. (**b**) Human sevoflurane anaesthesia (Sevo) versus wakefulness (two-sided paired-samples t-test, FDR-corrected for multiple comparisons) and versus post-anaesthetic recovery (two-sided paired-samples t-test, FDR-corrected for multiple comparisons); n =15. Box plots: central line, median; box limits, upper and lower quartiles; whiskers, 1.5× interquartile range. (**c**) Mouse wakefulness (n=10) versus medetomidine-isoflurane (MedIso; n = 14) and halothane (Halo; n = 19) anaesthesia data. P-values are from two-sided independent-samples t-test, FDR-corrected for multiple comparisons against Awake condition. Box plots: central line, median; box limits, upper and lower quartiles; whiskers, 1.5× interquartile range. (**d**) Macaque

wakefulness versus anaesthesia with propofol (Ppfl), sevoflurane (Sevo), and ketamine (Keta). N = 24 runs from 3 animals for Awake; 11 runs from 2 animals for Sevoflurane; 23 runs from 3 animals for Propofol; 22 runs from 3 animals for Ketamine anaesthesia. P-values are from linear mixed effects models (two-sided), FDR-corrected for multiple comparisons against Awake condition (see Methods). Box plots: central line, median; box limits, upper and lower quartiles; whiskers, 1.5× interquartile range. (**e**) Macaque DBS stimulation. N = 36 runs from 3 animals for Awake; 28 runs from 2 animals for anaesthesia (DBS-off); 31 runs from 2 animals for low amplitude centro-median thalamic DBS; 25 runs from 2 animals for high amplitude centro-median thalamic DBS; 18 runs from 1 animal for low amplitude ventro-lateral thalamic DBS; 18 runs from 1 animal for high amplitude ventro-lateral thalamic DBS. P-values are from linear mixed effects models (two-sided), FDR-corrected for multiple comparisons against propofol anaesthesia with no DBS (see Methods). Box plots: central line, median; box limits, upper and lower quartiles; whiskers, 1.5× interquartile range. Data-points with the same colour indicate the same animal. (**f**) Marmoset wakefulness versus anaesthesia with isoflurane (Iso), sevoflurane (Sevo) and propofol (Ppfl). N = 48 runs from 4 animals for each condition. P-values are from linear mixed effects models (two-sided), FDR-corrected for multiple comparisons against Awake condition (see Methods). Box plots: central line, median; box limits, upper and lower quartiles; whiskers, 1.5× interquartile range. Data-points with the same colour indicate the same animal. See Tables S28-S32 for full statistical reporting. Credits: Human head icon from pixabay.com. Mouse icon designed by CraftStarters.com. Macaque icon designed by Freepik.com. DBS and macaque thalamus illustrations adapted from Tasserie et al (2022), published under CC-BY license. Marmoset icon from pixabay.com.

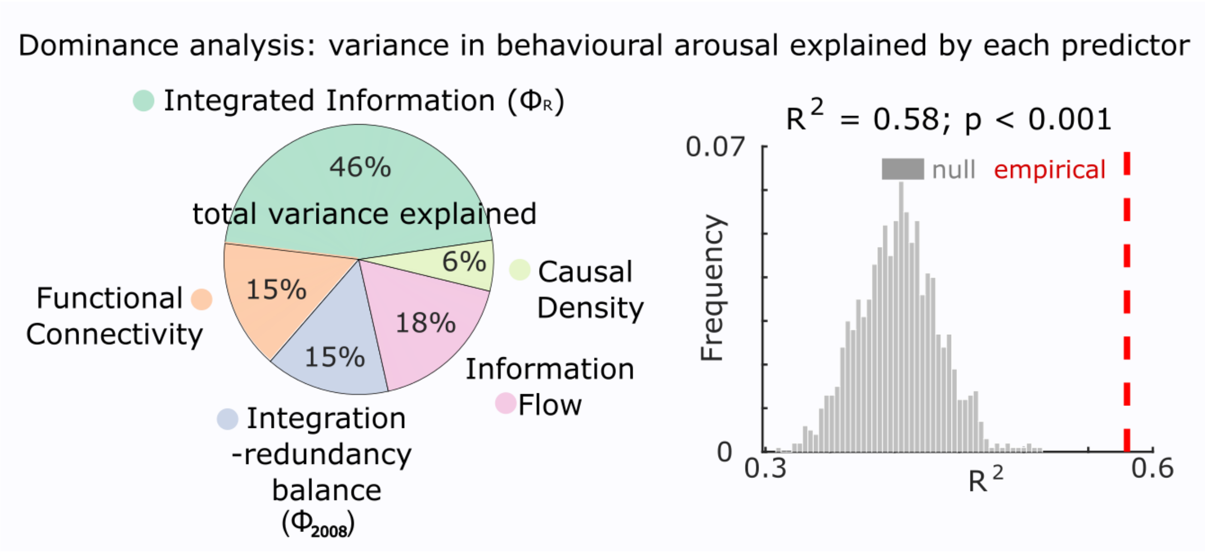

Figure S25. Dominance analysis including functional connectivity as predictor. Here, our predictors are the integrated information (Φ_R); the measure of integration-redundancy balance (Φ_{2008}); Causal Density; net information flow; and functional connectivity (see Methods for details of each). Regression target is the behavioural arousal score from each animal in the DBS macaque dataset (Tasserie et al., 2022). Percentage of relative importance is represented as a pie chart, revealing that integrated information is the predictor with highest relative importance, accounting for 46% of the total variance explained. We establish the statistical significance of the multiple linear regression model accounting for arousal score as a function of our fMRI information-dynamic measures model using a non-parametric permutation test (one-sided), by comparing the empirical variance explained against a null distribution of R^2 obtained from repeating the multiple regression with randomly reassigned arousal scores. The empirical variance explained is significantly greater than chance ($R^2 = 0.58$; $p < 0.001$).

Supplementary Tables

Parameter	Score Range	Description
Exploration of the surrounding world	0 to 2	• 0 = Total absence<br>• 1 = Small search of external clues<br>• 2 = Total investigation of the environment (e.g., head orientation to a sound)
Spontaneous movements	0 to 2	• 0 = Total absence<br>• 1 = Small torso and/or limb movement<br>• 2 = Large torso and/or limb movement
Shaking / prodding	0 to 2	• 0 = Total absence<br>• 1 = Small body movement<br>• 2 = Large body movement
Toe pinch	0 to 2	• 0 = Total absence<br>• 1 = Small reflex (weak body movement, eye blinking, or cardiac rate change)<br>• 2 = Clear reaction (strong body movement, eye blinking or opening, and cardiac rate change)
Eyes opening	0 to 2	• 0 = Total absence<br>• 1 = Small blinks or eye movements<br>• 2 = Full eye opening
Corneal reflex	0 to 1	• 0 = Absent<br>• 1 = Present

Table S1. Non-human primate behavioural arousal scale.

Gene	Full name
ADRA1A	Adrenergic receptor, α_{1A}
ADRA1D	Adrenergic receptor, α_{1D}
ADRA2A	Adrenergic receptor, α_{2A}
ADRB1	Adrenergic receptor, β_1
CALB1	Calbindin 1
CALB2	Calbindin 2 (calretinin)
CHRM1	Cholinergic receptor, muscarinic 1
CHRM2	Cholinergic receptor, muscarinic 2
CHRM4	Cholinergic receptor, muscarinic 4
CHRM5	Cholinergic receptor, muscarinic 5
CHRNA4	Nicotinic acetylcholine receptor subunit α_4
CHRNB2	Nicotinic acetylcholine receptor subunit β_2
CNR1	Cannabinoid receptor 1
DRD1	Dopamine receptor D1
DRD2	Dopamine receptor D2
DRD4	Dopamine receptor D4
GABBR2	Gamma-aminobutyric acid receptor, type B, subunit 2
GABRA1	Gamma-aminobutyric acid receptor subunit α_1
GABRA2	Gamma-aminobutyric acid receptor subunit α_2
GABRA3	Gamma-aminobutyric acid receptor subunit α_3
GABRA4	Gamma-aminobutyric acid receptor subunit α_4
GABRA5	Gamma-aminobutyric acid receptor subunit α_5
GABRB1	Gamma-aminobutyric acid receptor subunit β_1
GABRB2	Gamma-aminobutyric acid receptor subunit β_2
GABRB3	Gamma-aminobutyric acid receptor subunit β_3
GABRE	Gamma-aminobutyric acid receptor subunit ε
GABRG1	Gamma-aminobutyric acid receptor subunit γ_1
GABRG2	Gamma-aminobutyric acid receptor subunit γ_2
GABRG3	Gamma-aminobutyric acid receptor subunit γ_3
GALR1	Galanin receptor 1
GRIA1	Glutamate receptor, ionotropic, AMPA 1
GRIA2	Glutamate receptor, ionotropic, AMPA 2
GRIA3	Glutamate receptor, ionotropic, AMPA 3
GRIA4	Glutamate receptor, ionotropic, AMPA 4
GRIK1	Glutamate receptor, ionotropic, kainate 1
GRIK2	Glutamate receptor, ionotropic, kainate 2
GRIK3	Glutamate receptor, ionotropic, kainate 3
GRIK4	Glutamate receptor, ionotropic, kainate 4
GRIK5	Glutamate receptor, ionotropic, kainate 5
GRIN1	Glutamate receptor, ionotropic, NMDA 1
GRIN2A	Glutamate receptor, ionotropic, NMDA 2A
GRIN2B	Glutamate receptor, ionotropic, NMDA 2B
GRIN2C	Glutamate receptor, ionotropic, NMDA 2C
GRIN2D	Glutamate receptor, ionotropic, NMDA 2D
GRIN3A	Glutamate receptor, ionotropic, NMDA 3A
GRM1	Glutamate receptor, metabotropic 1
GRM2	Glutamate receptor, metabotropic 2
GRM3	Glutamate receptor, metabotropic 3
GRM4	Glutamate receptor, metabotropic 4
GRM5	Glutamate receptor, metabotropic 5

Table S2. List of brain-related genes included in the present study (1/2).

Gene	Full name
HCN1	Hyperpolarization-activated cyclic nucleotide-gated channel 1
HCRTR1	Hypocretin receptor 1
HDC	Histidine decarboxylase
HRH1	Histamine receptor H1
HRH2	Histamine receptor H2
HRH3	Histamine receptor H3
HTR1A	Serotonin receptor 1A
HTR2C	Serotonin receptor 2C
HTR3B	Serotonin receptor 3B
HTR4	Serotonin receptor 4
KCNK2	Potassium channel subfamily K member 2
MBP	Myelin basic protein
MCHR1	Melanin-concentrating hormone receptor 1
NPY1R	Neuropeptide Y receptor Y1
NTSR1	Neurotensin receptor 1
OPRK1	Opioid receptor, kappa 1
OPRL1	Opioid receptor-like 1
OPRM1	Opioid receptor, mu 1
OXTR	Oxytocin receptor
P2RY12	P2Y purinergic receptor 12
P2RY14	P2Y purinergic receptor 14
P2RY6	P2Y purinergic receptor 6
PLEKHB1	Pleckstrin homology domain containing B1
PVALB	Parvalbumin
SLC6A3	Dopamine transporter
SSTR2	Somatostatin receptor 2
SSTR4	Somatostatin receptor 4
STX1A	Syntaxin 1A
VIP	Vasoactive intestinal peptide
VIPR2	Vasoactive intestinal peptide receptor 2
SST	Somatostatin

Table S3. List of brain-related genes included in the present study (1/2).

Parameter	Symbol	Value
External current	I_0	0.382 nA
Excitatory scaling factor for I_0	W_E	1
Inhibitory scaling factor for I_0	W_I	0.7
Local excitatory recurrence	w_+	1.4
Excitatory synaptic coupling	J_{NMDA}	0.15 nA
Threshold for $F(I_n^{(E)})$	$I_{\text{thr}}^{(E)}$	0.403 nA
Threshold for $F(I_n^{(I)})$	$I_{\text{thr}}^{(I)}$	0.288 nA
Gain factor of $F(I_n^{(E)})$	g_E	310 nC^{-1}
Gain factor of $F(I_n^{(I)})$	g_I	615 nC^{-1}
Shape of $F(I_n^{(E)})$ around $I_{\text{thr}}^{(E)}$	d_E	0.16 s
Shape of $F(I_n^{(I)})$ around $I_{\text{thr}}^{(I)}$	d_I	0.087 s
Excitatory kinetic parameter	γ	0.641
Amplitude of uncorrelated Gaussian noise v_n	σ	0.01 nA
Time constant of NMDA	τ_{NMDA}	100 ms
Time constant of GABA	τ_{GABA_A}	10 ms
Neuromodulatory scaling factor	g_n^{NM}	1

Table S4. Dynamic Mean Field model parameters.

Species	Optimal G value
Human	1.76
Macaque	2.2
Macaque (anaesthesia)	1.9
Mouse	1.59

Table S5. G parameter for the species-specific models.

References

Afrasiabi, M., Redinbaugh, M.J., Phillips, J.M., Kambi, N.A., Mohanta, S., Raz, A., Haun, A.M., Saalmann, Y.B., 2021. Consciousness depends on integration between parietal cortex, striatum, and thalamus. Cell Syst 12, 363-373.e11. https://doi.org/10.1016/j.cels.2021.02.003

Albantakis, L., Barbosa, L., Findlay, G., Grasso, M., Haun, A.M., Marshall, W., Mayner, W.G., Zaeemzadeh, A., Boly, M., Juel, B.E., Sasai, S., Fujii, K., David, I., Hendren, J., Lang, J.P., Tononi, G., 2022. Integrated information theory (IIT) 4.0: Formulating the properties of phenomenal existence in physical terms. https://doi.org/10.48550/arXiv.2212.14787

Alexander-Bloch, A.F., Shou, H., Liu, S., Satterthwaite, T.D., Glahn, D.C., Shinohara, R.T., Vandekar, S.N., Raznahan, A., 2018. Supplementary Materials for "On testing for spatial correspondence between maps of human brain structure and function." NeuroImage 178, 540–551. https://doi.org/10.1016/j.neuroimage.2018.05.070

Alkire, M.T., McReynolds, J.R., Hahn, E.L., Trivedi, A.N., 2007. Thalamic microinjection of nicotine reverses sevoflurane-induced loss of righting reflex in the rat. Anesthesiology 107, 264–272. https://doi.org/10.1097/01.anes.0000270741.33766.24

Ay, N., 2015. Information Geometry on Complexity and Stochastic Interaction. Entropy 17, 2432–2458. https://doi.org/10.3390/e17042432

Azen, R., Budescu, D.V., 2003. The dominance analysis approach for comparing predictors in multiple regression. Psychological Methods 8, 129–148. https://doi.org/10.1037/1082-989X.8.2.129

Bakker, R., Wachtler, T., Diesmann, M., 2012. CoCoMac 2.0 and the future of tract-tracing databases. Front. Neuroinform. 6. https://doi.org/10.3389/fninf.2012.00030

Balduzzi, D., Tononi, G., 2008. Integrated information in discrete dynamical systems: Motivation and theoretical framework. PLoS Computational Biology 4, 1–18. https://doi.org/10.1371/journal.pcbi.1000091

Barbosa, L.S., Marshall, W., Albantakis, L., Tononi, G., 2021a. Mechanism Integrated Information. Entropy 23, 362. https://doi.org/10.3390/E23030362

Barbosa, L.S., Marshall, W., Albantakis, L., Tononi, G., 2021b. Mechanism Integrated Information. Entropy 23, 362. https://doi.org/10.3390/e23030362

Barbosa, L.S., Marshall, W., Streipert, S., Albantakis, L., Tononi, G., 2020. A measure for intrinsic information. Sci Rep 10, 18803. https://doi.org/10.1038/s41598-020-75943-4

Barrett, A.B., Mediano, P.A.M., 2019. The phi measure of integrated information is not well-defined for general physical systems. Journal of Consciousness Studies 26, 11–20.

Barrett, A.B., Seth, A.K., 2011. Practical Measures of Integrated Information for Time-Series Data. PLoS Comput Biol 7, 1001052. https://doi.org/10.1371/journal.pcbi.1001052

Barttfeld, P., Uhrig, L., Sitt, J.D., Sigman, M., Jarraya, B., Dehaene, S., 2015. Signature of consciousness in the dynamics of resting-state brain activity. Proceedings of the National Academy of Sciences 112, 887–892. https://doi.org/10.1073/pnas.1418031112

Bastos, A.M., Donoghue, J.A., Brincat, S.L., Mahnke, M., Yanar, J., Correa, J., Waite, A.S., Lundqvist, M., Roy, J., Brown, E.N., Miller, E.K., 2021. Neural effects of propofol-induced unconsciousness and its reversal using thalamic stimulation. eLife 10. https://doi.org/10.7554/ELIFE.60824

Behzadi Y, Restom K, Liau J, Liu TT, 2007. A component based noise correction method (CompCor) for BOLD and perfusion based fMRI. NeuroImage 37, 90–101.

Benjamini, Y., Hochberg, Y., 1995. Controlling the False Discovery Rate: A Practical and Powerful Approach to Multiple Testing. Journal of the Royal Statistical Society. Series B (Methodological) 57, 289–300.

Bjerke, I.E., Yates, S.C., Laja, A., Witter, M.P., Puchades, M.A., Bjaalie, J.G., Leergaard, T.B., 2021. Densities and numbers of calbindin and parvalbumin positive neurons

across the rat and mouse brain. iScience 24, 101906. https://doi.org/10.1016/j.isci.2020.101906
Brown, E.N., Lydic, R., Schiff, N.D., 2010. General Anesthesia, Sleep, and Coma. New England Journal of Medicine 27, 2638–50.
Burt, J.B., Demirtaş, M., Eckner, W.J., Navejar, N.M., Ji, J.L., Martin, W.J., Bernacchia, A., Anticevic, A., Murray, J.D., 2018. Hierarchy of transcriptomic specialization across human cortex captured by structural neuroimaging topography. Nature Neuroscience 21, 1251–1259. https://doi.org/10.1038/s41593-018-0195-0
Cabral, J., Kringelbach, M.L., Deco, G., 2017. Functional connectivity dynamically evolves on multiple time-scales over a static structural connectome: Models and mechanisms. NeuroImage 160, 84–96. https://doi.org/10.1016/j.neuroimage.2017.03.045
Chen, A., Sun, Yidi, Lei, Y., Li, Chao, Liao, S., Meng, J., Bai, Yiqin, Liu, Zhen, Liang, Z., Zhu, Z., Yuan, N., Yang, Hao, Wu, Z., Lin, F., Wang, K., Li, Mei, Zhang, S., Yang, M., Fei, T., Zhuang, Z., Huang, Yiming, Zhang, Y., Xu, Y., Cui, L., Zhang, R., Han, L., Sun, X., Chen, B., Li, W., Huangfu, B., Ma, K., Ma, J., Li, Z., Lin, Yikun, Wang, H., Zhong, Y., Zhang, Huifang, Yu, Q., Wang, Y., Liu, Xing, Peng, J., Liu, C., Chen, W., Pan, W., An, Y., Xia, S., Lu, Y., Wang, M., Song, X., Liu, Shuai, Wang, Z., Gong, C., Huang, X., Yuan, Y., Zhao, Y., Chai, Q., Tan, X., Liu, J., Zheng, M., Li, Shengkang, Huang, Yaling, Hong, Y., Huang, Z., Li, Min, Jin, M., Li, Yan, Zhang, Hui, Sun, S., Gao, L., Bai, Yinqi, Cheng, M., Hu, G., Liu, Shiping, Wang, B., Xiang, B., Li, Shuting, Li, H., Chen, M., Wang, S., Li, Minglong, Liu, W., Liu, Xin, Zhao, Q., Lisby, M., Wang, Jing, Fang, J., Lin, Yun, Xie, Q., Liu, Zhen, He, J., Xu, H., Huang, W., Mulder, J., Yang, Huanming, Sun, Yangang, Uhlen, M., Poo, M., Wang, Jian, Yao, J., Wei, W., Li, Yuxiang, Shen, Z., Liu, L., Liu, Zhiyong, Xu, X., Li, Chengyu, 2023. Single-cell spatial transcriptome reveals cell-type organization in the macaque cortex. Cell 186, 3726-3743.e24. https://doi.org/10.1016/j.cell.2023.06.009
Chernik, D.A., Gillings, D., Laine, H., Hendler, J., Silver, J.M., Davidson, A.B., Schwam, E.M., Siegel, J.L., 1990. Validity and reliability of the Observer's Assessment of Alertness/Sedation Scale: study with intravenous midazolam. J Clin Psychopharmacol 10, 244–251.
Cofré, R., Herzog, R., Mediano, P.A.M., Piccinini, J., Rosas, F.E., Perl, Y.S., Tagliazucchi, E., 2020. Whole-brain models to explore altered states of consciousness from the bottom up. Brain Sciences 10, 1–29. https://doi.org/10.3390/brainsci10090626
Coletta, L., Pagani, M., Whitesell, J.D., Harris, J.A., Bernhardt, B., Gozzi, A., 2020. Network structure of the mouse brain connectome with voxel resolution. Science Advances 6, 7187–7205. https://doi.org/10.1126/SCIADV.ABB7187/SUPPL_FILE/ABB7187_SM.PDF
Dean, J.G., Fields, C.W., Brito, M.A., Silverstein, B.H., Rybicki-Kler, C., Fryzel, A.M., Groenhout, T., Liu, T., Mashour, G.A., Pal, D., 2022. Inactivation of Prefrontal Cortex Attenuates Behavioral Arousal Induced by Stimulation of Basal Forebrain During Sevoflurane Anesthesia. Anesthesia & Analgesia 134, 1140. https://doi.org/10.1213/ANE.0000000000006011
Deco, G., Cruzat, J., Cabral, J., Whybrow, P.C., Logothetis, N.K., Kringelbach, M.L., 2018. Whole-Brain Multimodal Neuroimaging Model Using Serotonin Receptor Maps Explains Non-linear Functional Effects of LSD. Current Biology 28, 3065–3074. https://doi.org/10.1016/j.cub.2018.07.083
Deco, G., Kringelbach, M.L., 2014. Great expectations: Using whole-brain computational connectomics for understanding neuropsychiatric disorders. Neuron 84, 892–905. https://doi.org/10.1016/j.neuron.2014.08.034
Deco, G., Sanz Perl, Y., Bocaccio, H., Tagliazucchi, E., Kringelbach, M.L., 2022. The INSIDEOUT framework provides precise signatures of the balance of intrinsic and extrinsic dynamics in brain states. Commun Biol 5, 1–13. https://doi.org/10.1038/s42003-022-03505-7

Demirtaş, M., Burt, J.B., Helmer, M., Ji, J.L., Adkinson, B.D., Glasser, M.F., Van Essen, D.C., Sotiropoulos, S.N., Anticevic, A., Murray, J.D., 2019. Hierarchical Heterogeneity across Human Cortex Shapes Large-Scale Neural Dynamics. Neuron 101, 1181-1194.e13. https://doi.org/10.1016/j.neuron.2019.01.017
Dray, S., 2011. A New Perspective about Moran's Coefficient: Spatial Autocorrelation as a Linear Regression Problem. Moran系数的新视角：空间自相关视为线性回归问题. Geographical Analysis 43, 127–141. https://doi.org/10.1111/j.1538-4632.2011.00811.x
Edlow, B.L., Olchanyi, M., Freeman, H.J., Li, J., Maffei, C., Snider, S.B., Zöllei, L., Iglesias, J.E., Augustinack, J., Bodien, Y.G., Haynes, R.L., Greve, D.N., Diamond, B.R., Stevens, A., Giacino, J.T., Destrieux, C., van der Kouwe, A., Brown, E.N., Folkerth, R.D., Fischl, B., Kinney, H.C., 2024. Multimodal MRI reveals brainstem connections that sustain wakefulness in human consciousness. Science Translational Medicine 16, eadj4303. https://doi.org/10.1126/scitranslmed.adj4303
Fernandes, D.J., Ellegood, J., Askalan, R., Blakely, R.D., Dicicco-Bloom, E., Egan, S.E., Osborne, L.R., Powell, C.M., Raznahan, A., Robins, D.M., Salter, M.W., Sengar, A.S., Veenstra-VanderWeele, J., Henkelman, R.M., Lerch, J.P., 2017. Spatial gene expression analysis of neuroanatomical differences in mouse models. Neuroimage 163, 220–230. https://doi.org/10.1016/j.neuroimage.2017.08.065
Fischer, D.B., Boes, A.D., Demertzi, A., Evrard, H.C., Laureys, S., Edlow, B.L., Liu, H., Saper, C.B., Pascual-Leone, A., Fox, M.D., Geerling, J.C., 2016. A human brain network derived from coma-causing brainstem lesions. Neurology 87, 2427–2434. https://doi.org/10.1212/WNL.0000000000003404
Frey, S., Pandya, D.N., Chakravarty, M.M., Bailey, L., Petrides, M., Collins, D.L., 2011. An MRI based average macaque monkey stereotaxic atlas and space (MNI monkey space). Neuroimage 55, 1435–1442. https://doi.org/10.1016/j.neuroimage.2011.01.040
Friston, K.J., Harrison, L., Penny, W., 2003. Dynamic causal modelling. NeuroImage 19, 1273–1302. https://doi.org/10.1016/S1053-8119(03)00202-7
Gao, S., Proekt, A., Renier, N., Calderon, D.P., Pfaff, D.W., 2019. Activating an anterior nucleus gigantocellularis subpopulation triggers emergence from pharmacologically-induced coma in rodents. Nat Commun 10, 2897. https://doi.org/10.1038/s41467-019-10797-7
Gent, T.C., Bandarabadi, M., Herrera, C.G., Adamantidis, A.R., 2018. Thalamic dual control of sleep and wakefulness. Nat Neurosci 21, 974–984. https://doi.org/10.1038/s41593-018-0164-7
Glasser, M.F., Sotiropoulos, S.N., Wilson, A., Coalson, T.S., Fischl, B., Andersson, J.L., Xu, J., Jbabdi, S., Webster, M., Polimeni, J.R., Van Essen, D.C., Jenkinson, M., 2013. The Minimal Preprocessing Pipelines for the Human Connectome Project. Neuroimage 80, 105–124. https://doi.org/10.1016/j.neuroimage.2013.04.127
Grandjean, J., Canella, C., Anckaerts, C., Ayrancı, G., Bougacha, S., Bienert, T., Buehlmann, D., Coletta, L., Gallino, D., Gass, N., Garin, C.M., Abhay Nadkarni, N., Hübner, N., Karatas, M., Komaki, Y., Kreitz, S., Mandino, F., Mechling, A.E., Sato, C., Sauer, K., Shah, D., Strobelt, S., Takata, N., Wank, I., Wu, T., Yahata, N., Yun Yeow, L., Yee, Y., Aoki, I., Mallar Chakravarty, M., Chang, W.-T., Dhenain, M., von Elverfeldt, D., Harsan, L.-A., Hess, A., Jiang, T., Keliris, G.A., Lerch, J.P., Meyer-Lindenberg, A., Okano, H., Rudin, M., Sartorius, A., Van der Linden, A., Verhoye, M., Weber-Fahr, W., Wenderoth, N., Zerbi, V., Gozzi, A., Elverfeldt, von, der Linden, V., 2019. Common functional networks in the mouse brain revealed by multi-centre restingstate fMRI analysis NeuroImage. https://doi.org/10.1016/j.neuroimage.2019.116278
Griffith, V., 2014. A Principled Infotheoretic phi-like Measure. bioRxiv.
Gu, S., Pasqualetti, F., Cieslak, M., Telesford, Q.K., Yu, A.B., Kahn, A.E., Medaglia, J.D., Vettel, J.M., Miller, M.B., Grafton, S.T., Bassett, D.S., 2015. Controllability of

structural brain networks. Nature Communications 6, 8414. https://doi.org/10.1038/ncomms9414
Guang, J., Baker, H., Ben-Yishay Nizri, O., Firman, S., Werner-Reiss, U., Kapuller, V., Israel, Z., Bergman, H., 2021. Toward asleep DBS: cortico-basal ganglia spectral and coherence activity during interleaved propofol/ketamine sedation mimics NREM/REM sleep activity. NPJ Parkinsons Dis 7, 67. https://doi.org/10.1038/s41531-021-00211-9
Gutierrez-Barragan, D., Basson, M.A., Panzeri, S., Gozzi, A., 2019. Infraslow State Fluctuations Govern Spontaneous fMRI Network Dynamics. Current Biology 29, 2295-2306.e5. https://doi.org/10.1016/j.cub.2019.06.017
Gutierrez-Barragan, D., Singh, N.A., Alvino, F.G., Coletta, L., Rocchi, F., De Guzman, E., Galbusera, A., Uboldi, M., Panzeri, S., Gozzi, A., 2021. Unique spatiotemporal fMRI dynamics in the awake mouse brain. Current biology 32, 1–14. https://doi.org/10.1016/J.CUB.2021.12.015
Hansen, J.Y., Shafiei, G., Markello, R.D., Smart, K., Cox, S., Norgaard, M., Beliveau, V., Wu, Y., Gallezot, J.-D., Aumont, E., Servaes, S., Scala, S.G., DuBois, J.M., Wainstein, G., Bezgin, G., Funck, T., Schmitz, T.W., Spreng, R.N., Galovic, M., Koepp, M., Duncan, J.S., Coles, J.P., Fryer, T.D., Aigbirhio, F.I., McGinnity, C.J., Hammers, A., Soucy, J.-P., Baillet, S., Guimond, S., Hietala, J., Bedard, M.-A., Leyton, M., Kobayashi, E., Rosa-Neto, P., Ganz, M., Knudsen, G.M., Palomero-Gallagher, N., Shine, J.M., Carson, R.E., Tuominen, L., Dagher, A., Misic, B., 2022. Mapping neurotransmitter systems to the structural and functional organization of the human neocortex. Nature Neuroscience 25, 1569–1581.
Hartig, R., Glen, D., Jung, B., Logothetis, N.K., Paxinos, G., Garza-Villarreal, E.A., Messinger, A., Evrard, H.C., 2021. The Subcortical Atlas of the Rhesus Macaque (SARM) for neuroimaging. NeuroImage 235, 117996. https://doi.org/10.1016/j.neuroimage.2021.117996
Hata, J., Nakae, K., Tsukada, H., Woodward, A., Haga, Y., Iida, M., Uematsu, A., Seki, F., Ichinohe, N., Gong, R., Kaneko, T., Yoshimaru, D., Watakabe, A., Abe, H., Tani, T., Hamda, H.T., Gutierrez, C.E., Skibbe, H., Maeda, M., Papazian, F., Hagiya, K., Kishi, N., Ishii, S., Doya, K., Shimogori, T., Yamamori, T., Tanaka, K., Okano, H.J., Okano, H., 2023. Multi-modal brain magnetic resonance imaging database covering marmosets with a wide age range. Sci Data 10, 1–8. https://doi.org/10.1038/s41597-023-02121-2
Hawrylycz, M.J., Lein, E.S., Guillozet-Bongaarts, A.L., Shen, E.H., Ng, L., Miller, J.A., Van De Lagemaat, L.N., Smith, K.A., Ebbert, A., Riley, Z.L., Abajian, C., Beckmann, C.F., Bernard, A., Bertagnolli, D., Boe, A.F., Cartagena, P.M., Mallar Chakravarty, M., Chapin, M., Chong, J., Dalley, R.A., Daly, B.D., Dang, C., Datta, S., Dee, N., Dolbeare, T.A., Faber, V., Feng, D., Fowler, D.R., Goldy, J., Gregor, B.W., Haradon, Z., Haynor, D.R., Hohmann, J.G., Horvath, S., Howard, R.E., Jeromin, A., Jochim, J.M., Kinnunen, M., Lau, C., Lazarz, E.T., Lee, C., Lemon, T.A., Li, L., Li, Y., Morris, J.A., Overly, C.C., Parker, P.D., Parry, S.E., Reding, M., Royall, J.J., Schulkin, J., Sequeira, P.A., Slaughterbeck, C.R., Smith, S.C., Sodt, A.J., Sunkin, S.M., Swanson, B.E., Vawter, M.P., Williams, D., Wohnoutka, P., Ronald Zielke, H., Geschwind, D.H., Hof, P.R., Smith, S.M., Koch, C., Grant, S.G.N., Jones, A.R., 2012. An anatomically comprehensive atlas of the adult human brain transcriptome. Nature 489, 391–399. https://doi.org/10.1038/nature11405
Hemmings, H.C., Riegelhaupt, P.M., Kelz, M.B., Solt, K., Eckenhoff, R.G., Orser, B.A., Goldstein, P.A., 2019. Towards a Comprehensive Understanding of Anesthetic Mechanisms of Action: A Decade of Discovery. Trends in Pharmacological Sciences 40, 464–481. https://doi.org/10.1016/j.tips.2019.05.001
Herzog, R., Mediano, P.A.M., Rosas, F.E., Luppi, A.I., Perl, Y.S., Tagliazucchi, E., Kringelbach, M., Cofré, R., Deco, G., 2022. Neural mass modelling for the masses: Democratising access to whole-brain biophysical modelling with FastDMF. https://doi.org/10.1101/2022.04.11.487903

Huang, Z., Mashour, G.A., Hudetz, A.G., 2024. Propofol disrupts the functional core-matrix architecture of the thalamus in humans. Nat Commun 15, 7496. https://doi.org/10.1038/s41467-024-51837-1
Huels, E.R., Groenhout, T., Fields, C.W., Liu, T., Mashour, G.A., Pal, D., 2021. Inactivation of Prefrontal Cortex Delays Emergence From Sevoflurane Anesthesia. https://doi.org/10.3389/fnsys.2021.690717
Kim, Y., Yang, G.R., Pradhan, K., Venkataraju, K.U., Bota, M., García del Molino, L.C., Fitzgerald, G., Ram, K., He, M., Levine, J.M., Mitra, P., Huang, Z.J., Wang, X.-J., Osten, P., 2017. Brain-wide Maps Reveal Stereotyped Cell-Type-Based Cortical Architecture and Subcortical Sexual Dimorphism. Cell 171, 456-469.e22. https://doi.org/10.1016/j.cell.2017.09.020
Knox, J.E., Harris, K.D., Graddis, N., Whitesell, J.D., Zeng, H., Harris, J.A., Shea-Brown, E., Mihalas, S., 2018. High-resolution data-driven model of the mouse connectome. Network Neuroscience 3, 217–236. https://doi.org/10.1162/NETN_A_00066
Koçillari, L., Lorenz, G.M., Engel, N.M., Celotto, M., Curreli, S., Malerba, S.B., Engel, A.K., Fellin, T., Panzeri, S., 2024. Sampling bias corrections for accurate neural measures of redundant, unique, and synergistic information. https://doi.org/10.1101/2024.06.04.597303
Kötter, R., Wanke, E., 2005. Mapping brains without coordinates. Philosophical Transactions of the Royal Society B: Biological Sciences 360, 751–766. https://doi.org/10.1098/rstb.2005.1625
Kringelbach, M.L., Deco, G., 2020. Brain States and Transitions: Insights from Computational Neuroscience. Cell Reports 32, 108128. https://doi.org/10.1016/j.celrep.2020.108128
Langer, C., Ay, N., 2020. Complexity as causal information integration. Entropy 22, 1–32. https://doi.org/10.3390/e22101107
Laureys, S., Faymonville, M.E., Luxen, A., Lamy, M., Franck, G., Maquet, P., 2000. Restoration of thalamocortical connectivity after recovery from persistent vegetative state. Lancet 355, 1790–1791. https://doi.org/10.1016/s0140-6736(00)02271-6
Lein, E.S., Hawrylycz, M.J., Ao, N., Ayres, M., Bensinger, A., Bernard, A., Boe, A.F., Boguski, M.S., Brockway, K.S., Byrnes, E.J., Chen, Lin, Chen, Li, Chen, T.-M., Chi Chin, M., Chong, J., Crook, B.E., Czaplinska, A., Dang, C.N., Datta, S., Dee, N.R., Desaki, A.L., Desta, T., Diep, E., Dolbeare, T.A., Donelan, M.J., Dong, H.-W., Dougherty, J.G., Duncan, B.J., Ebbert, A.J., Eichele, G., Estin, L.K., Faber, C., Facer, B.A., Fields, R., Fischer, S.R., Fliss, T.P., Frensley, C., Gates, S.N., Glattfelder, K.J., Halverson, K.R., Hart, M.R., Hohmann, J.G., Howell, M.P., Jeung, D.P., Johnson, R.A., Karr, P.T., Kawal, R., Kidney, J.M., Knapik, R.H., Kuan, C.L., Lake, J.H., Laramee, A.R., Larsen, K.D., Lau, C., Lemon, T.A., Liang, A.J., Liu, Y., Luong, L.T., Michaels, J., Morgan, J.J., Morgan, R.J., Mortrud, M.T., Mosqueda, N.F., Ng, L.L., Ng, R., Orta, G.J., Overly, C.C., Pak, T.H., Parry, S.E., Pathak, S.D., Pearson, O.C., Puchalski, R.B., Riley, Z.L., Rockett, H.R., Rowland, S.A., Royall, J.J., Ruiz, M.J., Sarno, N.R., Schaffnit, K., Shapovalova, N.V., Sivisay, T., Slaughterbeck, C.R., Smith, S.C., Smith, K.A., Smith, B.I., Sodt, A.J., Stewart, N.N., Stumpf, K.-R., Sunkin, S.M., Sutram, M., Tam, A., Teemer, C.D., Thaller, C., Thompson, C.L., Varnam, L.R., Visel, A., Whitlock, R.M., Wohnoutka, P.E., Wolkey, C.K., Wong, V.Y., Wood, M., Yaylaoglu, M.B., Young, R.C., Youngstrom, B.L., Feng Yuan, X., Zhang, B., Zwingman, T.A., Jones, A.R., 2007. Genome-wide atlas of gene expression in the adult mouse brain. Nature 445, 168–176. https://doi.org/10.1038/nature05453
Li, J., Curley, W.H., Guerin, B., Dougherty, D.D., Dalca, A.V., Fischl, B., Horn, A., Edlow, B.L., 2021. Mapping the subcortical connectivity of the human default mode network. NeuroImage 245, 118758. https://doi.org/10.1016/j.neuroimage.2021.118758
Liu, C., Ye, F.Q., Yen, C.C.-C., Newman, J.D., Glen, D., Leopold, D.A., Silva, A.C., 2018. A digital 3D atlas of the marmoset brain based on multi-modal MRI. Neuroimage 169, 106–116. https://doi.org/10.1016/j.neuroimage.2017.12.004

Liu, J., Lee, H.J., Weitz, A.J., Fang, Z., Lin, P., Choy, M., Fisher, R., Pinskiy, V., Tolpygo, A., Mitra, P., Schiff, N., Lee, J.H., n.d. Frequency-selective control of cortical and subcortical networks by central thalamus. eLife 4, e09215. https://doi.org/10.7554/eLife.09215

Luppi, A.I., Cabral, J., Cofre, R., Mediano, P.A.M., Rosas, F.E., Qureshi, A.Y., Kuceyeski, A., Tagliazucchi, E., Raimondo, F., Deco, G., Shine, J.M., Kringelbach, M.L., Orio, P., Ching, S., Sanz-Perl, Y., Diringer, M.N., Stevens, R.D., Sitt, J.D., 2023a. Computational modelling in disorders of consciousness: closing the gap towards personalised models for restoring consciousness. Neuroimage 120162. https://doi.org/10.1016/j.neuroimage.2023.120162

Luppi, A.I., Craig, M.M., Pappas, I., Finoia, P., Williams, G.B., Allanson, J., Pickard, J.D., Owen, A.M., Naci, L., Menon, D.K., Stamatakis, E.A., 2019. Consciousness-specific dynamic interactions of brain integration and functional diversity. Nature Communications 10. https://doi.org/10.1038/s41467-019-12658-9

Luppi, A.I., Hansen, J.Y., Adapa, R., Carhart-Harris, R.L., Roseman, L., Timmermann, C., Golkowski, D., Ranft, A., Ilg, R., Jordan, D., Bonhomme, V., Vanhaudenhuyse, A., Demertzi, A., Jaquet, O., Bahri, M.A., Alnagger, N.L.N., Cardone, P., Peattie, A.R.D., Manktelow, A.E., de Araujo, D.B., Sensi, S.L., Owen, A.M., Naci, L., Menon, D.K., Misic, B., Stamatakis, E.A., 2023b. In vivo mapping of pharmacologically induced functional reorganization onto the human brain's neurotransmitter landscape. Science Advances 9, eadf8332. https://doi.org/10.1126/sciadv.adf8332

Luppi, A.I., Liu, Z.-Q., Hansen, J.Y., Cofre, R., Kuzmin, E., Froudist-Walsh, S., Palomero-Gallagher, N., Misic, B., 2024a. Benchmarking macaque brain gene expression for horizontal and vertical translation. https://doi.org/10.1101/2024.08.18.608440

Luppi, A.I., Mediano, P.A.M., Rosas, F.E., Allanson, J., Pickard, J.D., Williams, G.B., Craig, M.M., Finoia, P., Peattie, A.R.D., Coppola, P., Menon, D.K., Bor, D., Stamatakis, E.A., 2023c. Reduced emergent character of neural dynamics in patients with a disrupted connectome. Neuroimage 269, 119926. https://doi.org/10.1016/j.neuroimage.2023.119926

Luppi, A.I., Mediano, P.A.M., Rosas, F.E., Allanson, J., Pickard, J.D., Williams, G.B., Craig, M.M., Finoia, P., Peattie, A.R.D., Coppola, P., Owen, A.M., Naci, L., Menon, D.K., Bor, D., Stamatakis, E.A., 2022. Whole-brain modelling identifies distinct but convergent paths to unconsciousness in anaesthesia and disorders of consciousness. Communications biology 5, 384. https://doi.org/10.1038/S42003-022-03330-Y

Luppi, A.I., Rosas, F.E., Deco, G., Kringelbach, M.L., Mediano, P.A.M., 2023d. Information decomposition reveals hidden high-order contributions to temporal irreversibility. https://doi.org/10.48550/arXiv.2308.05664

Luppi, A.I., Stamatakis, E.A., 2021. Combining network topology and information theory to construct representative brain networks. Network Neuroscience 5, 96–124. https://doi.org/10.1162/netn_a_00170

Luppi, A.I., Uhrig, L., Tasserie, J., Signorelli, C.M., Stamatakis, E.A., Destexhe, A., Jarraya, B., Cofre, R., 2024b. Local orchestration of distributed functional patterns supporting loss and restoration of consciousness in the primate brain. Nat Commun 15, 2171. https://doi.org/10.1038/s41467-024-46382-w

Markello, R.D., Arnatkevičiūtė, A., Poline, J.B., Fulcher, B.D., Fornito, A., Misic, B., 2021. Standardizing workflows in imaging transcriptomics with the Abagen toolbox. eLife 10. https://doi.org/10.7554/ELIFE.72129

Markello, R.D., Misic, B., 2021. Comparing spatial null models for brain maps. NeuroImage 236, 118052. https://doi.org/10.1016/j.neuroimage.2021.118052

Marshall, W., Grasso, M., Mayner, W.G.P., Zaeemzadeh, A., Barbosa, L.S., Chastain, E., Findlay, G., Sasai, S., Albantakis, L., Tononi, G., 2023. System Integrated Information. Entropy (Basel) 25, 334. https://doi.org/10.3390/e25020334

Mashour, G.A., 2024. Anesthesia and the neurobiology of consciousness. Neuron. https://doi.org/10.1016/j.neuron.2024.03.002

Mashour, G.A., Pal, D., Brown, E.N., 2022. Prefrontal cortex as a key node in arousal circuitry. Trends Neurosci S0166-2236(22)00144–8. https://doi.org/10.1016/j.tins.2022.07.002

McCafferty, C., Gruenbaum, B.F., Tung, R., Li, J.-J., Zheng, X., Salvino, P., Vincent, P., Kratochvil, Z., Ryu, J.H., Khalaf, A., Swift, K., Akbari, R., Islam, W., Antwi, P., Johnson, E.A., Vitkovskiy, P., Sampognaro, J., Freedman, I.G., Kundishora, A., Depaulis, A., David, F., Crunelli, V., Sanganahalli, B.G., Herman, P., Hyder, F., Blumenfeld, H., 2023. Decreased but diverse activity of cortical and thalamic neurons in consciousness-impairing rodent absence seizures. Nat Commun 14, 117. https://doi.org/10.1038/s41467-022-35535-4

Mediano, P.A.M., Rosas, F.E., Bor, D., Seth, A.K., Barrett, A.B., 2022. The strength of weak integrated information theory. Trends in Cognitive Sciences 26, 646–655. https://doi.org/10.1016/j.tics.2022.04.008

Mediano, P.A.M., Rosas, F.E., Luppi, A.I., Carhart-Harris, R.L., Bor, D., Seth, A.K., Barrett, A.B., 2025. Toward a unified taxonomy of information dynamics via Integrated Information Decomposition. Proceedings of the National Academy of Sciences 122, e2423297122. https://doi.org/10.1073/pnas.2423297122

Mediano, P.A.M., Rosas, F.E., Luppi, A.I., Carhart-Harris, R.L., Bor, D., Seth, A.K., Barrett, A.B., 2021. Towards an extended taxonomy of information dynamics via Integrated Information Decomposition. arXiv. https://doi.org/10.48550/arXiv.2109.13186

Mhuircheartaigh, R.N., Rosenorn-Lanng, D., Wise, R., Jbabdi, S., Rogers, R., Tracey, I., 2010. Cortical and subcortical connectivity changes during decreasing levels of consciousness in humans: a functional magnetic resonance imaging study using propofol. The Journal of neuroscience : the official journal of the Society for Neuroscience 30, 9095–9102. https://doi.org/10.1523/JNEUROSCI.5516-09.2010

Michael Milham, A.P., Ai, L., Koo, B., Zhou, Y., Margulies, D.S., Schroeder Correspondence, C.E., Milham, M.P., Xu, T., line Amiez, C., Balezeau, F., Baxter, M.G., Blezer, E.L., Brochier, T., Chen, A., Croxson, P.L., Damatac, C.G., Dehaene, S., Everling, S., Fair, D.A., Fleysher, L., Freiwald, W., Froudist-Walsh, S., Griffiths, T.D., Guedj, C., Hadj-Bouziane, F., Ben Hamed, S., Harel, N., Hiba, B., Jarraya, B., Jung, B., Kastner, S., Christiaan Klink, P., Chai Kwok, S., Laland, K.N., Leopold, D.A., Mok, K., Morrison, J.H., Nacef, J., Nagy, J., Ortiz Rios, M., Petkov, C.I., Pinsk, M., Poirier, C., Procyk, E., Rajimehr, R., 2018. An Open Resource for Non-human Primate Imaging. Neuron 100, 61-74.e2. https://doi.org/10.1016/j.neuron.2018.08.039

Morais, P.L.A.G., Rubio-Garrido, P., Lima, R.M. de, Córdoba-Claros, A., Nascimento, E.S. de, Cavalcanti, J.S., Clascá, F., 2023. The arousal-related “central thalamus” stimulation site simultaneously innervates multiple high-level frontal and parietal areas. J. Neurosci. https://doi.org/10.1523/JNEUROSCI.1216-23.2023

Moruzzi, G., Magoun, H.W., 1949. Brain stem reticular formation and activation of the EEG. Electroencephalography and Clinical Neurophysiology 1, 455–473. https://doi.org/10.1016/0013-4694(49)90219-9

Müller, E.J., Munn, B., Hearne, L.J., Smith, J.B., Fulcher, B., Arnatkevičiūtė, A., Lurie, D.J., Cocchi, L., Shine, J.M., 2020. Core and matrix thalamic sub-populations relate to spatio-temporal cortical connectivity gradients. Neuroimage 222, 117224. https://doi.org/10.1016/j.neuroimage.2020.117224

Müller, F., Holze, F., Dolder, P., Ley, L., Vizeli, P., Soltermann, A., Liechti, M.E., Borgwardt, S., 2021. MDMA-induced changes in within-network connectivity contradict the specificity of these alterations for the effects of serotonergic hallucinogens. Neuropsychopharmacology 46, 545–553. https://doi.org/10.1038/s41386-020-00906-2

Muta, K., Hata, J., Kawaguchi, N., Haga, Y., Yoshimaru, D., Hagiya, K., Kaneko, T., Miyabe-Nishiwaki, T., Komaki, Y., Seki, F., Okano, H.J., Okano, H., 2023. Effect of sedatives or anesthetics on the measurement of resting brain function in common marmosets. Cerebral Cortex 33, 5148–5162. https://doi.org/10.1093/cercor/bhac406

ND Schiff, J.G., K. Kalmar, JD Victor, K. Baker, M. Gerber, 2007. Behavioural improvements with thalamic stimulation after severe traumatic brain injury. Nature 448, 600–603.
Oizumi, M., Albantakis, L., Tononi, G., 2014. From the Phenomenology to the Mechanisms of Consciousness: Integrated Information Theory 3.0. PLoS Computational Biology 10, e1003588. https://doi.org/10.1371/journal.pcbi.1003588
Oizumi, M., Amari, S.I., Yanagawa, T., Fujii, N., Tsuchiya, N., 2016a. Measuring Integrated Information from the Decoding Perspective. PLoS Computational Biology 12, e1004654. https://doi.org/10.1371/journal.pcbi.1004654
Oizumi, M., Tsuchiya, N., Amari, S.I., 2016b. Unified framework for information integration based on information geometry. Proceedings of the National Academy of Sciences of the United States of America 113, 14817–14822. https://doi.org/10.1073/pnas.1603583113
Pal, D., Dean, J.G., Liu, T., Li, D., Watson, C.J., Hudetz, A.G., Mashour, G.A., 2018. Differential Role of Prefrontal and Parietal Cortices in Controlling Level of Consciousness. Current Biology 28, 2145-2152.e5. https://doi.org/10.1016/j.cub.2018.05.025
Paxinos, G., Petrides, M., Evrard, H.C., 2008. The rhesus monkey brain in stereotaxic coordinates. Academic press.
Power, J.D., Barnes, K.A., Snyder, A.Z., Schlaggar, B.L., Petersen, S.E., 2012. Spurious but systematic correlations in functional connectivity MRI networks arise from subject motion. NeuroImage 59, 2142–2154. https://doi.org/10.1016/j.neuroimage.2011.10.018
Ranft, A., Golkowski, D., Kiel, T., Riedl, V., Kohl, P., Rohrer, G., Pientka, J., Berger, S., Thul, A., Maurer, M., Preibisch, C., Zimmer, C., Mashour, G.A., Kochs, E.F., Jordan, D., Ilg, R., 2016a. Neural Correlates of Sevoflurane-induced Unconsciousness Identified by Simultaneous Functional Magnetic Resonance Imaging and Electroencephalography. Anesthesiology 125, 861–872. https://doi.org/10.1097/ALN.0000000000001322
Ranft, A., Golkowski, D., Kiel, T., Riedl, V., Kohl, P., Rohrer, G., Pientka, J., Berger, S., Thul, A., Maurer, M., Preibisch, C., Zimmer, C., Mashour, G.A., Kochs, E.F., Jordan, D., Ilg, R., 2016b. Neural Correlates of Sevoflurane-induced Unconsciousness Identified by Simultaneous Functional Magnetic Resonance Imaging and Electroencephalography. Anesthesiology 125, 861–872. https://doi.org/10.1097/ALN.0000000000001322
Redinbaugh, M.J., Afrasiabi, M., Phillips, J.M., Kambi, N.A., Mohanta, S., Raz, A., Saalmann, Y.B., 2022. Thalamic deep brain stimulation paradigm to reduce consciousness: Cortico-striatal dynamics implicated in mechanisms of consciousness. PLoS Comput Biol 18, e1010294. https://doi.org/10.1371/journal.pcbi.1010294
Redinbaugh, M.J., Phillips, J.M., Kambi, N.A., Mohanta, S., Andryk, S., Dooley, G.L., Afrasiabi, M., Raz, A., Saalmann, Y.B., 2020. Thalamus modulates consciousness via layer-specific control of cortex. Neuron 106, 66-75.e12.
Saleem, K.S., Logothetis, N.K., 2012. A Combined MRI and Histology Atlas of the Rhesus Monkey Brain in Stereotaxic Coordinates. Academic Press.
Schiff, N.D., 2008. Central thalamic contributions to arousal regulation and neurological disorders of consciousness. Ann N Y Acad Sci 1129, 105–118. https://doi.org/10.1196/annals.1417.029
Seth, A.K., Barrett, A.B., Barnett, L., 2011. Causal density and integrated information as measures of conscious level. Philosophical Transactions of the Royal Society A: Mathematical, Physical and Engineering Sciences 369, 3748–3767. https://doi.org/10.1098/rsta.2011.0079
Shen, K., Bezgin, G., Schirner, M., Ritter, P., Everling, S., McIntosh, A.R., 2019. A macaque connectome for large-scale network simulations in TheVirtualBrain. Scientific Data 6. https://doi.org/10.1038/s41597-019-0129-z

Shine, J.M., 2020. The thalamus integrates the macrosystems of the brain to facilitate complex, adaptive brain network dynamics. Progress in Neurobiology 101951. https://doi.org/10.1016/j.pneurobio.2020.101951

Shine, J.M., Lewis, L.D., Garrett, D.D., Hwang, K., 2023. The impact of the human thalamus on brain-wide information processing. Nat Rev Neurosci 24, 416–430. https://doi.org/10.1038/s41583-023-00701-0

Shine, J.M., Müller, E.J., Munn, B., Cabral, J., Moran, R.J., Breakspear, M., 2021. Computational models link cellular mechanisms of neuromodulation to large-scale neural dynamics. Nature Neuroscience 24, 1–12. https://doi.org/10.1038/s41593-021-00824-6

Signorelli, C.M., Uhrig, L., Kringelbach, M., Jarraya, B., Deco, G., 2020. Hierarchical disruption in the cortex of anesthetized monkeys as a new signature of consciousness loss. NeuroImage 117618. https://doi.org/10.1016/j.neuroimage.2020.117618

Sirmpilatze, N., Klink, P.C., 2020. RheMAP: Non-linear warps between common rhesus macaque brain templates. https://doi.org/10.5281/zenodo.3668510

Solt, K., Van Dort, C.J., Chemali, J.J., Taylor, N.E., Kenny, J.D., Brown, E.N., 2014. Electrical stimulation of the ventral Tegmental area induces reanimation from general anesthesia. Anesthesiology 121, 311–319. https://doi.org/10.1097/ALN.0000000000000117

Spindler, L.R.B., Luppi, A.I., Adapa, R.M., Craig, M.M., Coppola, P., Peattie, A.R.D., Manktelow, A.E., Finoia, P., Sahakian, B.J., Williams, G.B., Allanson, J., Pickard, J.D., Menon, D.K., Stamatakis, E.A., 2021. Dopaminergic brainstem disconnection is common to pharmacological and pathological consciousness perturbation. Proceedings of the National Academy of Sciences of the United States of America 118, e2026289118. https://doi.org/10.1073/pnas.2026289118

Sporns, O., Kötter, R., 2004. Motifs in Brain Networks. PLOS Biology 2, e369. https://doi.org/10.1371/journal.pbio.0020369

Staunton, H., 2008. Arousal by stimulation of deep-brain nuclei. Nature 452, E1; discussion E1-2. https://doi.org/10.1038/nature06574

Steriade, M., Glenn, L.L., 1982. Neocortical and caudate projections of intralaminar thalamic neurons and their synaptic excitation from midbrain reticular core. Journal of Neurophysiology 48, 352–371. https://doi.org/10.1152/jn.1982.48.2.352

Tasserie, J., Grigis, A., Uhrig, L., Dupont, M., Amadon, A., Jarraya, B., 2020. Pypreclin: An automatic pipeline for macaque functional MRI preprocessing. NeuroImage 207. https://doi.org/10.1016/j.neuroimage.2019.116353

Tasserie, J., Uhrig, L., Sitt, J.D., Manasova, D., Dupont, M., Dehaene, S., Jarraya, B., 2022. Deep brain stimulation of the thalamus restores signatures of consciousness in a nonhuman primate model. Sci. Adv 8, 5547. https://doi.org/10.1126/SCIADV.ABL5547

Taylor, N.E., Van Dort, C.J., Kenny, J.D., Pei, J., Guidera, J.A., Vlasov, K.Y., Lee, J.T., Boyden, E.S., Brown, E.N., Solt, K., 2016. Optogenetic activation of Dopamine neurons in the ventral tegmental area induces reanimation from general anesthesia. Proceedings of the National Academy of Sciences of the United States of America 113, 12826–12831. https://doi.org/10.1073/pnas.1614340113

Tegmark, M., 2016. Improved Measures of Integrated Information. PLoS Computational Biology 12. https://doi.org/10.1371/journal.pcbi.1005123

Tian, Y., Margulies, D., Breakspear, M., Zalesky, A., 2020. Topographic organization of the human subcortex unveiled with functional connectivity gradients. Nature Neuroscience 23, 1421–1432. https://doi.org/10.1101/2020.01.13.903542

Toker, D., Sommer, F.T., 2019. Information integration in large brain networks. PLOS Computational Biology 15, e1006807. https://doi.org/10.1371/journal.pcbi.1006807

Tononi, G., Edelman, G.M., 1998. Consciousness and complexity. Science 282, 1846–1851. https://doi.org/10.1126/science.282.5395.1846

Tononi, G., Sporns, O., Edelman, G.M., 1994. A measure for brain complexity: relating functional segregation and integration in the nervous system. Proceedings of the National Academy of Sciences 91, 5033–5037. https://doi.org/10.1073/pnas.91.11.5033

Uhrig, L., Dehaene, S., Jarraya, B., 2014. A Hierarchy of Responses to Auditory Regularities in the Macaque Brain. J. Neurosci. 34, 1127–1132. https://doi.org/10.1523/JNEUROSCI.3165-13.2014

Uhrig, L., Janssen, D., Dehaene, S., Jarraya, B., 2016. Cerebral responses to local and global auditory novelty under general anesthesia. Neuroimage 141, 326–340. https://doi.org/10.1016/j.neuroimage.2016.08.004

Uhrig, L., Sitt, J.D., Jacob, A., Tasserie, J., Barttfeld, P., Dupont, M., Dehaene, S., Jarraya, B., 2018. Resting-state Dynamics as a Cortical Signature of Anesthesia in Monkeys. Anesthesiology 129, 942–958. https://doi.org/10.1097/ALN.0000000000002336

Vanduffel, W., Fize, D., Mandeville, J.B., Nelissen, K., Van Hecke, P., Rosen, B.R., Tootell, R.B., Orban, G.A., 2001. Visual motion processing investigated using contrast agent-enhanced fMRI in awake behaving monkeys. Neuron 32, 565–577. https://doi.org/10.1016/s0896-6273(01)00502-5

Váša, F., Mišić, B., 2022. Null models in network neuroscience. Nat Rev Neurosci 23, 493–504. https://doi.org/10.1038/s41583-022-00601-9

Vincent, J.L., Patel, G.H., Fox, M.D., Snyder, A.Z., Baker, J.T., Van Essen, D.C., Zempel, J.M., Snyder, L.H., Corbetta, M., Raichle, M.E., 2007. Intrinsic functional architecture in the anaesthetized monkey brain. Nature 447, 83–86. https://doi.org/10.1038/nature05758

Vincent, K.F., Zhang, E.R., Cho, A.J., Kato-Miyabe, R., Mallari, O.G., Moody, O.A., Obert, D.P., Park, G.H., Solt, K., 2024. Electrical stimulation of the ventral tegmental area restores consciousness from sevoflurane-, dexmedetomidine-, and fentanyl-induced unconsciousness in rats. Brain Stimul 17, 687–697. https://doi.org/10.1016/j.brs.2024.05.012

Vos de Wael, R., Benkarim, O., Paquola, C., Lariviere, S., Royer, J., Tavakol, S., Xu, T., Hong, S.J., Langs, G., Valk, S., Misic, B., Milham, M., Margulies, D., Smallwood, J., Bernhardt, B.C., 2020. BrainSpace: a toolbox for the analysis of macroscale gradients in neuroimaging and connectomics datasets. Communications Biology 3. https://doi.org/10.1038/s42003-020-0794-7

Wagner, H.H., Dray, S., 2015. Generating spatially constrained null models for irregularly spaced data using Moran spectral randomization methods. Methods in Ecology and Evolution 6, 1169–1178. https://doi.org/10.1111/2041-210X.12407

Wang, P., Kong, R., Kong, X., Liégeois, R., Orban, C., Deco, G., Van Den Heuvel, M.P., Yeo, B.T.T., 2019. Inversion of a large-scale circuit model reveals a cortical hierarchy in the dynamic resting human brain. Science Advances 5, 1–12. https://doi.org/10.1126/sciadv.aat7854

Wang, Q., Ding, S.-L., Li, Y., Royall, J., Feng, D., Lesnar, P., Graddis, N., Naeemi, M., Facer, B., Ho, A., Dolbeare, T., Blanchard, B., Dee, N., Wakeman, W., Hirokawa, K.E., Szafer, A., Sunkin, S.M., Oh, S.W., Bernard, A., Phillips, J.W., Hawrylycz, M., Koch, C., Zeng, H., Harris, J.A., Ng, L., 2020. The Allen Mouse Brain Common Coordinate Framework: A 3D Reference Atlas. Cell 181, 936-953.e20. https://doi.org/10.1016/j.cell.2020.04.007

Whitesell, J.D., Liska, A., Coletta, L., Hirokawa, K.E., Bohn, P., Williford, A., Groblewski, P.A., Graddis, N., Kuan, L., Knox, J.E., Ho, A., Wakeman, W., Nicovich, P.R., Nguyen, T.N., van Velthoven, C.T.J., Garren, E., Fong, O., Naeemi, M., Henry, A.M., Dee, N., Smith, K.A., Levi, B., Feng, D., Ng, L., Tasic, B., Zeng, H., Mihalas, S., Gozzi, A., Harris, J.A., 2021. Regional, Layer, and Cell-Type-Specific Connectivity of the Mouse Default Mode Network. Neuron 109, 545-559.e8. https://doi.org/10.1016/J.NEURON.2020.11.011

Whitfield-Gabrieli, S., Nieto-Castanon, A., 2012. Conn: A Functional Connectivity Toolbox for Correlated and Anticorrelated Brain Networks. Brain Connectivity 2, 125–141. https://doi.org/10.1089/brain.2012.0073
Xu, J., Galardi, M.M., Pok, B., Patel, K.K., Zhao, C.W., Andrews, J.P., Singla, S., McCafferty, C.P., Feng, L., Musonza, E.T., Kundishora, A.J., Gummadavelli, A., Gerrard, J.L., Laubach, M., Schiff, N.D., Blumenfeld, H., 2020. Thalamic Stimulation Improves Postictal Cortical Arousal and Behavior. J Neurosci 40, 7343–7354. https://doi.org/10.1523/JNEUROSCI.1370-20.2020
Yee, Y., Fernandes, D.J., French, L., Ellegood, J., Cahill, L.S., Vousden, D.A., Spencer Noakes, L., Scholz, J., van Eede, M.C., Nieman, B.J., Sled, J.G., Lerch, J.P., 2018. Structural covariance of brain region volumes is associated with both structural connectivity and transcriptomic similarity. NeuroImage 179, 357–372. https://doi.org/10.1016/j.neuroimage.2018.05.028
Yeh, F.-C., Panesar, S., Fernandes, D., Meola, A., Yoshino, M., Fernandez-Miranda, J.C., Vettel, J.M., Verstynen, T., 2018. Population-averaged atlas of the macroscale human structural connectome and its network topology. NeuroImage 178, 57–68. https://doi.org/10.1016/j.neuroimage.2018.05.027
Yeh, F.-C., Wedeen, V.J., Tseng, W.-Y.I., 2011. Estimation of fiber orientation and spin density distribution by diffusion deconvolution. NeuroImage 55, 1054–1062. https://doi.org/10.1016/J.NEUROIMAGE.2010.11.087
Zalesky, A., Fornito, A., Bullmore, E.T., 2010. Network-based statistic: Identifying differences in brain networks. NeuroImage 53, 1197–1207. https://doi.org/10.1016/j.neuroimage.2010.06.041
Zarkali, A., Luppi, A.I., Stamatakis, E.A., Reeves, S., McColgan, P., Leyland, L.-A., Lees, A.J., Weil, R.S., 2022. Changes in dynamic transitions between integrated and segregated states underlie visual hallucinations in Parkinson’s disease. Commun Biol 5, 1–15. https://doi.org/10.1038/s42003-022-03903-x
