# Supplementary material for: Convergent transcriptomic and connectomic controllers of information integration and its anaesthetic breakdown across mammalian brains
Source: Nat Hum Behav. 2026 Jan 28;10(4):777–802. doi: 10.1038/s41562-025-02381-5 (PMC13121024; doi:10.1038/s41562-025-02381-5)
Supplement: Supplementary file 2 — Reporting Summary [file 41562_2025_2381_MOESM2_ESM.pdf]

nature portfolio

Corresponding author(s): ANDREA LUPPI

Last updated by author(s): Oct 16, 2025

Reporting Summary

Nature Portfolio wishes to improve the reproducibility of the work that we publish. This form provides structure for consistency and transparency in reporting. For further information on Nature Portfolio policies, see our Editorial Policies and the Editorial Policy Checklist.

Statistics

For all statistical analyses, confirm that the following items are present in the figure legend, table legend, main text, or Methods section.

n/a	Confirmed	
☐	☒	The exact sample size (*n*) for each experimental group/condition, given as a discrete number and unit of measurement
☐	☒	A statement on whether measurements were taken from distinct samples or whether the same sample was measured repeatedly
☐	☒	The statistical test(s) used AND whether they are one- or two-sided<br>*Only common tests should be described solely by name; describe more complex techniques in the Methods section.*
☐	☒	A description of all covariates tested
☐	☒	A description of any assumptions or corrections, such as tests of normality and adjustment for multiple comparisons
☐	☒	A full description of the statistical parameters including central tendency (e.g. means) or other basic estimates (e.g. regression coefficient) AND variation (e.g. standard deviation) or associated estimates of uncertainty (e.g. confidence intervals)
☐	☒	For null hypothesis testing, the test statistic (e.g. *F*, *t*, *r*) with confidence intervals, effect sizes, degrees of freedom and *P* value noted<br>*Give P values as exact values whenever suitable.*
☒	☐	For Bayesian analysis, information on the choice of priors and Markov chain Monte Carlo settings
☒	☐	For hierarchical and complex designs, identification of the appropriate level for tests and full reporting of outcomes
☐	☒	Estimates of effect sizes (e.g. Cohen's *d*, Pearson's *r*), indicating how they were calculated

Our web collection on statistics for biologists contains articles on many of the points above.

Software and code

Policy information about availability of computer code

Data collection	The Python processing for PreClinical data pipeline, Pypreclin version 1.0.1, is freely available at https://github.com/neurospin/pypreclin. FMRIB Software Library (FSL) is freely available online (http://www.fmrib.ox.ac.uk/fsl/; version accessed February 4, 2018).
Data analysis	Analysis was performed in MTALAB version 2019a and 2024b, and Python 3.11. The CONN toolbox version 17f is freely available at http://www.nitrc.org/projects/conn/. DSI Studio (version 2022) is freely available at https://dsi-studio.labsolver.org/. The abagen toolbox (version 0.1.4) is available at https://github.com/rmarkello/abagen. The RheMap toolbox(version 1.4) is available at https://doi.org/10.5281/zenodo.3668510. The BrainSpace toolbox for generation of Moran spectral surrogates is available at https://brainspace.readthedocs.io/en/latest/. The Python toolbox for Dominance Analysis is freely available at https://github.com/dominance-analysis/dominance-analysis. The Brain Connectivity Toolbox is available online at https://sites.google.com/site/bctnet/. The JIDT toolbox is available at https://github.com/jlizier/jidt. The FastDMF code for whole-brain modelling at https://www.gitlab.com/concog/fastdmf. MATLAB/Octave and Python code to compute measures of Integrated Information Decomposition of timeseries with the Gaussian MMI solver is freely available at https://github.com/Imperial-MIND-lab/integrated-info-decomp.

For manuscripts utilizing custom algorithms or software that are central to the research but not yet described in published literature, software must be made available to editors and reviewers. We strongly encourage code deposition in a community repository (e.g. GitHub). See the Nature Portfolio guidelines for submitting code & software for further information.

Data

Policy information about availability of data

All manuscripts must include a data availability statement. This statement should provide the following information, where applicable:

- Accession codes, unique identifiers, or web links for publicly available datasets
- A description of any restrictions on data availability
- For clinical datasets or third party data, please ensure that the statement adheres to our policy

For the human sevoflurane dataset, data are available from author D.G. through academic collaboration. For the macaque Multi-anaesthesia dataset, raw data are available for access from author B.J. through academic collaboration. For the macaque DBS dataset, raw data are available for access from author B.J. through academic collaboration. For the mouse dataset, data are available from author A.G. The marmoset fMRI data are available from author K.M. through academic collaboration. The HCP DWI data in SRC format are available online (http://brain.labsolver.org/diffusion-mri-data/hcp-dmri-data). The macaque structural connectome is available on Zenodo at https://doi.org/10.5281/zenodo.1471588. The CoCoMac database is also available online at http://cocomac.g-node.org/main/index.php?. Preprocessed macaque dMRI data in DSI Studio format are available on Zendo (DOI: 10.5281/zenodo.6321168). The mouse connectome is available from author A.G. The marmoset structural connectivity data are available online at https://doi.org/10.24475/bminds.mri.thj.4624. Human gene expression data 66 are available from the Allen Human Brain Atlas at http://human.brain-map.org/static/download. Mouse gene expression data 67 are available at https://mouse.brain-map.org/. Macaque cortical gene expression data from 68 are available at https://macaque.digital-brain.cn/spatial-omics. The dataset is provided by Brain Science Data Center, Chinese Academy of Sciences (https://braindatacenter.cn/). The macaque gene expression data resampled to the Regional Mapping atlas are available at https://github.com/netneurolab/luppi-genes-receptors-macaque. Mouse regional PV+ neuron count data from Kim, Yang, et al (2017) are available at http://mouse.brainarchitecture.org/cellcounts/ost/. Macaque parvalbumin density data from immunohistochemistry for several regions of the macaque cortex are available from the Supplementary Materials of Burt et al (2018) 90. Immunohistochemically-derived measurements of the relative prevalence of calbindin-positive and parvalbumin-positive neurons in different thalamic nuclei are available from the Supplementary Material of from Bjerke et al 85. Source Data are provided with this article.

Research involving human participants, their data, or biological material

Policy information about studies with human participants or human data. See also policy information about sex, gender (identity/presentation), and sexual orientation and race, ethnicity and racism.

Reporting on sex and gender	Each dataset had been previously collected. The human sevoflurane dataset only included male participants. The design is within-subjects, and our focus was not on comparing groups or inter-individual differences but rather on comparing states of anaesthesia. The HCP dataset includes both men and women.
Reporting on race, ethnicity, or other socially relevant groupings	No grouping by race, ethnicity, or socieconomic status was performed.
Population characteristics	See Life sciences reporting.
Recruitment	Data acquisition took place between June and December 2013. Participants approaches the research team to seek participation. healthy adult men were recruited through campus notices and personal contact, and compensated for their participation in the study. Further exclusion criteria were the following: physical status other than American Society of Anesthesiologists physical status I, chronic intake of medication or drugs, hardness of hearing or deafness, absence of fluency in German, known or suspected disposition to malignant hyperthermia, acute hepatic porphyria, history of halothane hepatitis, obesity with a body mass index more than 30 kg/m2, gastrointestinal disorders with a disposition for gastroesophageal regurgitation, known or suspected difficult airway, and presence of metal implants.<br><br>HCP dataset: Detailed information about the recruitment, acquisition and imaging is provided in the dedicated HCP publications.
Ethics oversight	All HCP scanning protocols were approved by the local Institutional Review Board at Washington University in St. Louis. Sevoflurane dataset: The ethics committee of the medical school of the Technische Universitat Munchen (Munchen, Germany) approved the current study.

Note that full information on the approval of the study protocol must also be provided in the manuscript.

Field-specific reporting

Please select the one below that is the best fit for your research. If you are not sure, read the appropriate sections before making your selection.

☒ Life sciences ☐ Behavioural & social sciences ☐ Ecological, evolutionary & environmental sciences

For a reference copy of the document with all sections, see nature.com/documents/nr-reporting-summary-flat.pdf

Life sciences study design

All studies must disclose on these points even when the disclosure is negative.

Sample size	This study used previously collected data. No power analysis was performed prior to data collection, but the sample sizes are within the range reported in the literature, and each of the datasets included here has been individually published before: Ranft 2016 Anesthesiology (Human); Uhrig 2018 Anesthesiology (Macaque multi-anaesthesia); Tasserie 2022 Science Advances (macaque DBS); Muta 2023 Cerebral Cortex (marmoset); Gutierrez-Barragan 2021 Current Biology (mouse).<br><br>Human sevoflurane dataset: n=20 participants were recruited; each contributed data for awake, recovery, and 3 different anaesthesia levels<br><br>Macaque datasets: Five rhesus macaques were included for analyses (Macaca mulatta, one male, monkey J, and four females, monkey A, K, Ki, and R, 5-8 kg, 8-12 yr of age). For the DBS dataset, details were provided in (Tasserie et al., 2022). Five male rhesus macaques (Macaca mulatta, 9 to 17 years and 7.5 to 9.1 kg) were included, three for the awake (non-DBS) experiments (monkeys B, J, and Y) and two for the DBS experiments (monkeys N and T). No statistical methods were used to determine sample size, but these sample sizes are in line with similar studies in the field, due to the ethical and practical challenges of neuroscience research with nonhuman primates. Each animal contributed multiple scans. For the Multi-anaesthesia dataset, after data exclusion (see below), N=24 runs from 3 animals for Awake; 11 runs from 2 animals for Sevoflurane; 23 runs from 3 animals for Propofol; 22 runs from 3 animals for Ketamine anaesthesia, for the Multi-anaesthesia dataset. For the DBS dataset, after data ezclusions (see below) N=36 runs from 3 animals for Awake; 28 runs from 2 animals for anaesthesia (DBS-off); 31 runs from 2 animals for low amplitude centro-median thalamic DBS; 25 runs from 2 animals for high amplitude centro-median thalamic DBS; 18 runs from 1 animal for low amplitude ventro-lateral thalamic DBS; 18 runs from 1 animal for high amplitude ventro-lateral thalamic DBS.<br><br>Marmoset dataset: Three male and one female healthy common marmosets (C. jacchus) between 3 and 6 years of age were included. All marmosets were examined 12 times to collect functional MRI data in all conditions.<br><br>Mouse: N=10 for awake condition; N=19 for halothane; N=14 for med-sio anaesthesia.
Data exclusions	Sevoflurane dataset: A total of 16 volunteers completed the full protocol and were included in our analyses; one participant was excluded due to high motion, leaving N=15 for analysis.<br><br>Macaque: See "Noise and artifact removal" section below, for quality control criteria used to exclude individual trials from analysis. Exclusion was done prior to analysis.<br><br>Marmoset: no exclusions<br>Mouse: no exclusions
Replication	We replicated our results 5 times in 4 different mammalian species (human, two macaque datasets; marmoset; mouse), and with different anaesthetics (human: sevoflurane at 3 different doses; macaque: 3 anaesthetics; marmoset: 3 anaesthetics; mouse: 2 anaesthetics).. We also replicated results with two different measures of parvalbumin density. Thalamic results in the mouse were replicated between transcriptomics and immunohistochemistry.<br><br>For the human data, each volunteer contributed to 3 different anaesthesia levels. For the Multi-anaesthesia dataset, after data exclusion (see below), N=24 runs from 3 animals for Awake; 11 runs from 2 animals for Sevoflurane; 23 runs from 3 animals for Propofol; 22 runs from 3 animals for Ketamine anaesthesia, for the Multi-anaesthesia dataset. For the DBS dataset, after data exclusions (see below) N=36 runs from 3 animals for Awake; 28 runs from 2 animals for anaesthesia (DBS-off); 31 runs from 2 animals for low amplitude centro-median thalamic DBS; 25 runs from 2 animals for high amplitude centro-median thalamic DBS; 18 runs from 1 animal for low amplitude ventro-lateral thalamic DBS; 18 runs from 1 animal for high amplitude ventro-lateral thalamic DBS. For the marmoset data, each animal contributed 12 scans for each of the 3 anaesthetics.
Randomization	Human sevoflurane data, marmoset data: every individual was included in every condition, so no allocation was required.<br><br>Macaque datasets: Three monkeys were used for each condition: awake state (monkeys A, K, and J), ketamine (monkeys K, R and Ki), propofol (monkeys K, R, and J), sevoflurane (monkeys Ki, R, and J).<br>For the multi-anaesthesia dataset, the acquisitions were performed over 5 years. Whenever possible, animals were scanned in different anesthesia (propofol, sevoflurane, ketamine) conditions.<br>Monkey R: moderate propofol, deep propofol, moderate sevoflurane, deep sevoflurane, ketamine<br>Monkey K: moderate propofol, deep propofol, ketamine<br>Monkey K could not be scanned under moderate sevoflurane and deep sevoflurane anesthesia, because this monkey had health issues not related with this study.<br>Monkey Ki: moderate sevoflurane, deep sevoflurane, ketamine<br>Monkey J: moderate propofol, deep propofol, moderate sevoflurane, deep sevoflurane<br>For the awake condition, monkeys need to have a headpost an be trained for awake fMRI studies. 2 monkeys of the multi-anaesthesia dataset could be scanned in the awake condition. Monkey K: and monkey J. The third monkey of this group was monkey A.<br><br>Regarding the DBS dataset, animals and experimental conditions (different location and stimulation for resting state) were randomly chosen using a Matlab function.<br><br>For the macaque and marmoset datasets, the design involves taking multiple samples from the same animals in different conditions, and we controlled for this with linear mixed effects modelling.<br><br>Mouse dataset: each individual only took part in one condition.. Animals were of the same sex (all male), age, and bred in identical conditions in the same vivarium. Independent-samples tests were used to account for different identities.

Blinding	No blinding is possible, since anaesthetised state is tested by means of behavioural responsiveness.

Reporting for specific materials, systems and methods

We require information from authors about some types of materials, experimental systems and methods used in many studies. Here, indicate whether each material, system or method listed is relevant to your study. If you are not sure if a list item applies to your research, read the appropriate section before selecting a response.

Materials & experimental systems

n/a	Involved in the study
☒	☐ Antibodies
☒	☐ Eukaryotic cell lines
☒	☐ Palaeontology and archaeology
☐	☒ Animals and other organisms
☒	☐ Clinical data
☒	☐ Dual use research of concern
☒	☐ Plants

Methods

n/a	Involved in the study
☒	☐ ChIP-seq
☒	☐ Flow cytometry
☐	☒ MRI-based neuroimaging

Animals and other research organisms

Policy information about studies involving animals; ARRIVE guidelines recommended for reporting animal research, and Sex and Gender in Research

Laboratory animals	Macaque Multi-anaesthesia dataset: Macaca mulatta (one male and four females, 8-12 yr of age).<br>Macaque DBS dataset: Macaca mulatta (five males, 9 to 17 years).<br><br>Marmoset dataset: Three male and one female healthy common marmosets (C. jacchus) between 3 and 6 years of age were included. All marmosets were examined 8 times to collect functional MRI data in all conditions.<br><br>Mouse: N=10 for awake condition; N=19 for halothane; N=14 for med-sio anaesthesia.
Wild animals	No wild animals used.
Reporting on sex	For the macaque anaesthesia dataset, five rhesus macaques were included for analyses (Macaca mulatta, one male, monkey J, and four females, monkey A, K, Ki, and R, 5-8 kg, 8-12 yr of age).<br><br>For the macaque DBS dataset, five male rhesus macaques (Macaca mulatta, 9 to 17 years and 7.5 to 9.1 kg) were included, three for the awake (non-DBS) experiments (monkeys B, J, and Y) and two for the DBS experiments (monkeys N and T).<br><br>For the marmoset dataset, three male and one female animals were included.<br><br>Mouse dataset: Adult (< 6 months old) male C57BL/6J mice were used throughout the study.<br><br>Sex was not considered in this study. Because of the small sample sizes, the sex balance per group could not be secured.<br>Only males were included in the DBS dataset in order to avoid the menstrual cycle and hormone variations.
Field-collected samples	None
Ethics oversight	Human sevoflurane: The ethics committee of the medical school of the Technische Universität München (München, Germany) approved the current study.<br><br>Macaque: All procedures are in agreement with the European Convention for the Protection of Vertebrate Animals used for Experimental and Other Scientific Purposes (Directive 2010/63/EU) and the National Institutes of Health's Guide for the Care and Use of Laboratory Animals. Animal studies were approved by the institutional Ethical Committee (Commissariat à l'Energie atomique et aux Énergies alternatives; Fontenay aux Roses, France; protocols CETEA \#10-003 and 12-086). All procedures are in agreement with 2010/63/UE, 86-406, 12-086 and 16-040.<br><br>Marmoset: This study was approved by the Animal Experiment Committees at the RIKEN Center for Brain Science (CBS) and was conducted per the guidelines for Conducting Animal Experiments of RIKEN CBS.<br><br>Mouse: In vivo experiments were conducted in accordance with the Italian law (DL 26/214, EU 63/2010, Ministero della Sanita, Roma) and with the National Institute of Health recommendations for the care and use of laboratory animals 29. The animal research protocols for this study were reviewed and approved by the Italian Ministry of Health and the animal care committee of Istituto Italiano di Tecnologia (IIT).

Note that full information on the approval of the study protocol must also be provided in the manuscript.

Plants

Seed stocks	N/A
Novel plant genotypes	N/A
Authentication	N/A

Magnetic resonance imaging

Experimental design

Design type

resting-state for all datasets

Design specifications

Human sevoflurane: : five scanning sessions: awake, 2 vol%, 3 vol% burst-suppression, and recovery. 350 volumes acquired for each scan.

Macaque multi-anaesthesia: For the anaesthesia dataset, a total of 157 functional magnetic imaging runs were acquired (Uhrig et al., 2018): Awake, 31 runs (monkey A, 4 runs; monkey J, 18 runs; monkey K, 9 runs), Ketamine, 25 runs (monkey K, 8 runs; monkey Ki, 7 runs; monkey R, 10 runs), Light Propofol, 25 runs (monkey J, 2 runs; monkey K, 10 runs; monkey R, 12 runs), Deep Propofol, 31 runs (monkey J, 9 runs; monkey K, 10 runs; monkey R, 12 runs), Light Sevoflurane, 25 runs (monkey J, 5 runs; monkey Ki, 10 runs; monkey R, 10 runs), Deep Sevoflurane anaesthesia, 20 runs (monkey J, 2 runs; monkey Ki, 8 runs; monkey R, 11 runs). For details, check the supplementary tables for (Barttfeld et al., 2015; Uhrig et al., 2018; Signorelli et al., 2021) (http://links.ww.com/ALN/B756).

Macaque DBS: For the DBS dataset, a total of 199 Resting State functional MRI runs were acquired: Awake 47 runs (monkey B: 18 runs; monkey J: 13 runs; monkey Y: 16 runs), anaesthesia (DBS-off) 38 runs (monkey N: 16 runs,; monkey T: 22 runs), low amplitude centro-median thalamic DBS 36 runs (monkey N: 18 runs; monkey T: 18 runs), low amplitude ventro-lateral thalamic DBS 20 runs (monkey T), high amplitude centro-median thalamic DBS 38 runs (monkey N: 17 runs; monkey T: 21 runs), and high amplitude ventro-lateral thalamic DBS 20 runs (monkey T: 20 runs).

Marmoset: Functional imaging was performed 12 times per animal, per condition. After awake data were firstly collected, and sedate/anesthetic data were done in a random order for sedate/anesthetic condition with an interval of 1 month between each examination in each individual. scan time = 310s.

Mouse: Mice under halothane anesthesia (n = 19) were scanned for a total of 1600 time points, total acquisition time of 32 minutes. Awake and medetomidine-isoflurane rsfMRI scans were acquired for a total time of 32 minutes.

Behavioral performance measures

Loss of behavioural responsiveness was used to determine depth of anaesthesia.
Sevoflurane dataset: loss of consciousness was judged by the loss of responsiveness
(LOR) to the repeatedly spoken command "squeeze my hand" two consecutive times.

For the macaque datasets, We used a preclinical behavioural scale adapted from Uhrig et al66 to assess the arousal levels of the monkeys. This scale, based on the Human Observers Assessment of Alertness and Sedation Scale113 and previously utilised in non-human primate (NHP) research114, was used consistently across all experimental conditions, in both datasets.
The assessment encompassed six criteria as follows:

- exploration of the surrounding world, from 0 to 2:
0 = total absence,
1 = small search of external clue,
2 = total investigation of the environment (such as head orientation to a sound);
- spontaneous movements, from 0 to 2:
0 = total absence,
1 = small torso and/or limb movement,
2= large torso and/or limb movement
- shaking / prodding, from 0 to 2:
0= total absence,
1= small body movement,
2 = large body movement;
- toe pinch, from 0 to 2:
0 = total absence,
1 = small reflex (weak body movement or eye blinking or cardiac rate change),
2 = clear reaction (strong body movement and eye blinking or eye opening and cardiac rate change);

- eyes opening, from 0 to 2:
0 = total absence,
1 = small blinks or eye movements,
2 = full eye opening;
- corneal reflex, from 0 to 1:
0 = absent,
1 = present.

Acquisition

Imaging type(s)

Functional and anatomical for all datasets

Field strength

3T for human; 9.4 T for marmoset; 7.0 for mouse; 3T for macaque.

Sequence & imaging parameters

Human sevoflurane: Data acquisition was carried out on a 3-Tesla magnetic resonance imaging scanner (Achieva Quasar Dual 3.0T 16CH, The Netherlands) with an eight-channel, phased-array head coil. The data were collected using a gradient echo planar imaging sequence (echo time = 30 ms, repetition time (TR) = 1.838 s, flip angle = 75°, field of view = 220 × 220 mm2, matrix = 72 × 72, 32 slices, slice thickness = 3 mm, and 1 mm interslice gap; 700-s acquisition time, resulting in 350 functional volumes). The anatomical scan was acquired before the functional scan using a T1-weighted MPRAGE sequence with 240 × 240 × 170 voxels (1×1×1 mm voxel size) covering the whole brain.

Macaque: For the awake condition, monkeys were implanted with a magnetic resonance compatible head post and trained to sit in the sphinx position in a primate chair (Uhrig, Dehaene and Jarraya, 2014)). For the awake scanning sessions, monkeys sat inside the dark magnetic resonance imaging scanner without any task and the eye position was monitored at 120 Hz (Iscan Inc., USA). The eye-tracking was performed to make sure that the monkeys were awake during the whole scanning session and not sleeping. The eye movements were not regressed out from rfMRI data. For the anesthesia sessions, animals were positioned in a sphinx position, mechanically ventilated, and their physiologic parameters were monitored. No eye-tracking was performed in anesthetic conditions. For the anesthesia dataset, before each scanning session, a contrast agent, monocrystalline iron oxide nanoparticle (Feraheme, AMAG Pharmaceuticals, USA; 10 mg/kg, intravenous), was injected into the monkey's saphenous vein (Vanduffel et al., 2001). Monkeys were scanned at rest on a 3-Tesla horizontal scanner (Siemens Tim Trio, Germany) with a single transmit-receive surface coil customized to monkeys. Each functional scan consisted of gradient-echo planar whole-brain images (repetition time = 2,400 ms; echo time = 20 ms; 1.5-mm3 voxel size; 500 brain volumes per run).

For the DBS dataset, monkeys were scanned at rest on a 3-Tesla horizontal scanner (Siemens, Prisma Fit, Erlanger Germany) with a customized eight-channel phased- array surface coil (KU Leuven, Belgium). The parameters of the functional MRI sequences were: echo planar imaging (EPI), TR = 1250 ms, echo time (TE) = 14.20 ms, 1.25-mm isotropic voxel size and 500 brain volumes per run.

Marmoset: An ultra-high field MRI system with a static magnetic field strength of 9.4 T (Bruker BioSpin, Ettlingen, Germany), a custom-made 8-channel receiver coil for the marmoset head (Takashima Seisakusho Co., Ltd, Tokyo, Japan), and a 154 mm inner diameter transmitter coil (Bruker BioSpin, Ettlingen, Germany) were used to collect structural and functional data. Structural data and T2-weighted images were imaged using rapid acquisition with relaxation enhancement (RARE) sequence with the following conditions and parameters: time repetition (TR)=4331 ms, time echo (TE) = 15.0 ms, FOV = 42.0 × 28.0 × 36.0 mm, matrix size = 120 × 80 voxels, resolution = 0.35 × 0.35 mm, slice thickness = 0.7 mm, number of slices = 52, scan time = 1 min and 26 s, RARE factor = 4. Functional images were captured using a gradient
recalled echo-planar imaging (EPI) sequence with the following conditions and parameters: TR = 2,000 ms, TE = 16.0 mm, FOV = 42.0 × 28.0 × 36.0,mm matrix size = 60 × 40 voxels, resolution = 0.7 × 0.7 mm, slice thickness = 0.7 mm, number of slices = 52, repetition = 155, scan time = 310s.

Mouse: All scans were acquired at the IIT laboratory in Rovereto (Italy) on a 7.0 Tesla MRI scanner (Bruker Biospin, Ettlingen) with a BGA-9 gradient set, a 72 mm birdcage transmit coil, and a four-channel (awake, halothane) or three-channel (medetomidine-isoflurane) solenoid
receive coil. Awake and medetomidine-isoflurane rsfMRI scans were acquired using a single-shot echo planar imaging (EPI) sequence with the following parameters: TR/TE=1000/15 ms, flip angle=60 degrees, matrix=100 x 100, FOV=2.3 x 2.3 cm, 18 coronal slices (voxel-size 230 x 230 x 600 mm), slice thickness=600 mm and 1920 time points, for a total time of 32 minutes. Mice under halothane anesthesia (n = 19) were scanned with a TR/TE=1200/15ms, flip angle=60 degrees, matrix=100 x 100, 24 coronal slices (voxel-size 200 x 200 x 500 mm), for a total of 1600 time points, total acquisition time of 32 minutes.

Area of acquisition

Whole brain for all datasets.

Diffusion MRI ☒ Used ☐ Not used

Parameters

Macaque: Anatomical (structural) connectivity data were derived from the recent macaque connectome of (Shen et al., 2019), which combines diffusion MRI tractrography with axonal tract-tracing studies, representing the most complete representation of the macaque connectome available to date. Structural (i.e., anatomical) connectivity data are expressed as a matrix in which the 82 cortical regions of interest are displayed in x-axis and y-axis. Each cell of the matrix represents the strength of the anatomical connection between any pair of cortical areas.

Human: The dMRI data were from the HCP dataset. The spatial resolution was 1.25 mm isotropic. TR=5500ms, TE=89.50ms. The b-

values were 1000, 2000, and 3000 s/mm2. The total number of diffusion sampling directions was 90, 90, and 90 for each of the shells in addition to 6 b0 images.

Preprocessing

Preprocessing software	Human: Preprocessing of the functional MRI data for both datasets followed the same standard workflow as in our previous studies, and was implemented in the CONN toolbox (http://www.nitrc.org/projects/conn), version 17f [81].<br><br>Macaque: Images were preprocessed using Pypreclin (Python preclinical pipeline) (Tasserie et al., Neuroimage 2020).<br><br>Marmoset: SPM (Wellcome Trust Center for Neuroimaging, London, UK).<br><br>Mouse: AFNI, FSL, ANTs
Normalization	Human: Direct normalisation to MNI space (nonlinear) using the segmented grey matter image from each volunteer's high-resolution T1-weighted image, together with an a priori grey matter template.<br><br>Macaque: Functional images were reoriented, realigned, and rigidly coregistered to the anatomical template of the monkey Montreal Neurologic Institute (Montreal, Canada) space with the use of Python programming language and Oxford Centre Functional Magnetic Resonance Imaging of the Brain Software Library software (United Kingdom, http://www.fmrib.ox.ac.uk/fsl/; accessed February 4, 2018) (Uhrig, Dehaene and Jarraya, 2014)).<br><br>Mouse: spatially registered (ANTs registration suite) to an in-house mouse brain template with a spatial resolution of 0.23 x 0.23 x 0.6mm3.<br><br>Marmoset: The voxels were spatially standardized<br>by normalization, which aligns the voxels to the standard brain image to correct for structural differences between individuals.. functional data were parcellated into 70 regions in the cerebral cortex, corresponding to regions of the marmoset MBM atlas
Normalization template	Human: Schaefer-100 parcellation in MNI-152 volumetric template, 2x2x2mm isotropic resolution.<br><br>Macaque: Data were parcellated according to the Regional Map parcellation (Kötter and Wanke, 2005). This parcellation comprises 82 cortical ROIs (41 per hemisphere; Supplementary Table 4).<br><br>Marmoset: functional data were parcellated into 70 regions in the cerebral cortex, corresponding to regions of the marmoset MBM atlas<br><br>Mouse: data were parcellated into 162 cortical and subcortical symmetric regions from the Allen Mouse Brain Atlas (CCFv3).
Noise and artifact removal	Human: Denoising followed the anatomical CompCor (aCompCor) method of removing cardiac and motion artifacts, by regressing out of each individual's functional data the first 5 principal components corresponding to white matter signal, and the first 5 components corresponding to cerebrospinal fluid signal, as well as six subject-specific realignment parameters (three translations and three rotations) and their first- order temporal derivatives, and nuisance regressors identified by the software ART 82. The subject-specific denoised BOLD signal time-series were linearly detrended and band-pass filtered between 0.008 and 0.09 Hz to eliminate both low-frequency drift effects and high-frequency noise.<br><br>Macaque:<br>Voxel time series were filtered with low-pass (0.05-Hz cutoff) and high-pass (0.0025-Hz cutoff) filters and a zero-phase fast-Fourier notch filter (0.03 Hz) to remove an artifactual pure frequency present in all the data (Barttfeld et al., 2015; Uhrig et al., 2018). Furthermore, an extra quality control (QC) cleaning procedure was performed to ensure the quality of the data after time-series extraction72. This quality control procedure is based on trial-by-trial visual inspection by an expert neuroimager (C.M.S.), and it is the same as was previously implemented in Signorelli et al72. Its adoption ensures that we employ consistent criteria across our two datasets, by adopting the more stringent of the two. We plotted the time series of each region, as well as the static functional connectivity matrix (FC), the dynamic connectivity (dFC) and a Fourier analysis to detect unconventional spikes of activity. For each dataset, visual inspection was first used to become familiar with the characteristics of the entire dataset: how the amplitude spectrum, timeseries, FC and dynamic FC look. Subsequently, each trial was inspected again with particular focus on two main types of potential artefacts. The first one may correspond to issues with the acquisition and is given by stereotyped sinusoidal oscillatory patterns without variation. The second one may correspond to a head or other movement not corrected properly by our preprocessing procedure. This last artefact can be sometimes recognized by bursts or peaks of activity. Sinusoidal activity generates artificially high functional correlation and peak of frequencies in the Amplitude spectrum plot. Uncorrected movements generate peaks of activity with high functional correlation and sections of high functional correlations in the dynamical FC matrix. If we observed any of these anomalies we rejected the trial, opting to adopt a conservative policy. See Figures S17-S19 for examples of artifact-free and rejected trials.<br><br>As a result, for the Multi-Anaesthesia data set a total of 119 runs are analysed in subsequent sections (the same as used in Signorelli et al. 72): awake state 24 runs, ketamine anaesthesia 22 runs, light propofol anaesthesia 21 runs, deep propofol anaesthesia 23 runs, light sevoflurane anaesthesia 18 runs, deep sevoflurane anaesthesia 11 runs. For the DBS data set, a total of 156 runs are analysed in subsequent sections: awake state 36 runs, Off condition (propofol anaesthesia without stimulation) 28 runs, low-amplitude CT stimulation 31 runs, low-amplitude VT stimulation 18 runs, high-amplitude CT stimulation 25 runs, high-amplitude VT stimulation 18 runs.<br><br>Marmoset: Estimation and correction of geometric distortions induced by magnetic susceptibility were performed with the

top-up tool of the FMRIB Software Library (FSL) software (FMRIB, Oxford, UK) because all cross-sections were imaged with a single excitation in EPI. Slice timing correction was performed to correct for signal acquisition timing discrepancies in each section. Realignment was applied to compensate for head movements caused by body movements. The deviations in 6 directions were obtained: x (left/right), y (front/back), z (up/down), pitch (rotational direction of nodding and looking up), roll (rotational direction of moving the ear closer to the shoulder), and yaw (rotational direction of looking left/right). For each measurement time point (TR), the deviation from the reference time point, and the first functional brain image, was determined; and the image was moved and rotated by the rigid body model based on this deviation. The method of finding the parameters of the linear transformation was used to minimize the difference between the first functional brain image and the affine transformation of the series of functional brain images to be corrected, by calculating convergence using the method of least squares.
After correcting the spatial scale error between the structural and functional images with co-registration, segmentation was performed to provide information on the tissue to which each voxel belongs in terms of brain tissue classification. The voxels were spatially standardized
by normalization, which aligns the voxels to the standard brain image to correct for structural differences between individuals. Smoothing was applied to suppress excessive voxel value fluctuations within individuals and apply normal probability field theory. Functional data were
smoothed using spatial convolution with a Gaussian kernel of 2 voxels (7 mm). Then, physiological noise was denoised using ordinary least squares regression with cerebrospinal fluid pulsation, heart rate, and respiratory artifacts as regressors. Temporal band pass filtering was performed by frequency filtering (0.01–0.1 Hz) using the fMRI denoising pipeline of CONN.

Mouse: the first 2 minutes of the time series were removed to account for thermal gradient equilibration. RsfMRI timeseries were then time despiked (3dDespike, AFNI), motion corrected (MCFLIRT, FSL), skull stripped (FAST, FSL) and spatially registered (ANTs registration suite) to an in-house mouse brain template with a spatial resolution of 0.23 x 0.23 x 0.6mm3. Denoising involved the regression of 25 nuisance parameters. These were: average cerebral spinal fluid signal plus 24 motion parameters determined from the 3 translation and rotation parameters estimated during motion correction, their temporal derivatives and corresponding squared regressors. No global signal regression was employed. In-scanner head motion was quantified via calculations of frame-wise displacement (FD). Average FD levels in awake conditions were comparable to those obtained in anesthetized animals (halothane) under artificial ventilation (p = 0.13, Student t test) 29. To rule out a contribution of residual head-motion, we further introduced frame-wise fMRI scrubbing (FD > 0.075 mm). The resulting time series were band-pass filtered (0.01-0.1 Hz band) and then spatially smoothed with a Gaussian kernel of 0.5 mm full width at half maximum. Finally, the timeseries were trimmed to ensure that the same number of timepoints were included for all animals, resulting in 1414 volumes per animal.

Volume censoring

Human: the artifact rejection tool (ART), implemented in the CONN toolbox, was used to identify and regress out outlying volumes, as part of the CompCor denoising procedure described above. The default CONN settings of 5 global signal z-values and 0.9mm were used.

Mouse: To rule out a contribution of residual head-motion, we further introduced frame-wise fMRI scrubbing (FD > 0.075 mm).

Statistical modeling & inference

Model type and settings

We used correlation against an autocorrelation-preserving null distribution to test the spatial association between regional change in integrated information and gene expression maps. For marmoset and macaque we used linear mixed-effects modelling. For human we used repeated-measures t-tests. For mouse we used between-subjects t-tests.

Effect(s) tested

We tested whether integrated information was different between awake/recovery and anaesthetised conditions. We also tested whether its regional change was spatially associated with gene expression maps. We also used partial correlation and linear mixed-modelling, using motion (mean framewise displacement) as covariate of no interest.

Specify type of analysis: ☐ Whole brain ☐ ROI-based ☒ Both

Anatomical location(s)

Human:M Schaefer fucntional atlas; Macaque: Regional Mapping atlas. Mouse: Allen atlas; Marmoset: MBM atlas.

Statistic type for inference

(See Eklund et al. 2016)

Spatial correlation with autocorrelation-preserving nulls. Effect sizes are provided as Hedge's measure of standardised difference g.

Correction

correction for multiple comparisons against the same condition was carried out using the False Discovery Rate procedure

Models & analysis

n/a	Involved in the study
☐	☒ Functional and/or effective connectivity
☐	☒ Graph analysis
☒	☐ Multivariate modeling or predictive analysis

Functional and/or effective connectivity

We used Integrated Information obtained from Information Decomposition (see Methods) between each pair of regions.

Graph analysis

We used network-based generative (biophysical) models, and we also used network control energy. Both

Graph analysis

were based on the structural connectome.
